# Pan-KRAS inhibitor disables oncogenic signalling and tumour growth

**DOI:** 10.1038/s41586-023-06123-3

**Published:** 2023-05-31

**Authors:** Dongsung Kim, Lorenz Herdeis, Dorothea Rudolph, Yulei Zhao, Jark Böttcher, Alberto Vides, Carlos I. Ayala-Santos, Yasin Pourfarjam, Antonio Cuevas-Navarro, Jenny Y. Xue, Andreas Mantoulidis, Joachim Bröker, Tobias Wunberg, Otmar Schaaf, Johannes Popow, Bernhard Wolkerstorfer, Katrin Gabriele Kropatsch, Rui Qu, Elisa de Stanchina, Ben Sang, Chuanchuan Li, Darryl B. McConnell, Norbert Kraut, Piro Lito

**Affiliations:** 1 Human Oncology and Pathogenesis Program, Memorial Sloan Kettering Cancer Center, New York, NY USA; 2 Department of Medicine, Memorial Sloan Kettering Cancer Center, New York, NY USA; 3 Boehringer Ingelheim, Vienna, Austria; 4 Antitumor Assessment Core Facility, Memorial Sloan Kettering Cancer Center, New York, NY USA; 5 Department of Medicine, Weill Cornell Medical College, New York, NY USA

**Keywords:** Targeted therapies, Drug discovery, Cell signalling

## Abstract

KRAS is one of the most commonly mutated proteins in cancer, and efforts to directly inhibit its function have been continuing for decades. The most successful of these has been the development of covalent allele-specific inhibitors that trap KRAS G12C in its inactive conformation and suppress tumour growth in patients^1–7^. Whether inactive-state selective inhibition can be used to therapeutically target non-G12C KRAS mutants remains under investigation. Here we report the discovery and characterization of a non-covalent inhibitor that binds preferentially and with high affinity to the inactive state of KRAS while sparing NRAS and HRAS. Although limited to only a few amino acids, the evolutionary divergence in the GTPase domain of RAS isoforms was sufficient to impart orthosteric and allosteric constraints for KRAS selectivity. The inhibitor blocked nucleotide exchange to prevent the activation of wild-type KRAS and a broad range of KRAS mutants, including G12A/C/D/F/V/S, G13C/D, V14I, L19F, Q22K, D33E, Q61H, K117N and A146V/T. Inhibition of downstream signalling and proliferation was restricted to cancer cells harbouring mutant KRAS, and drug treatment suppressed KRAS mutant tumour growth in mice, without having a detrimental effect on animal weight. Our study suggests that most KRAS oncoproteins cycle between an active state and an inactive state in cancer cells and are dependent on nucleotide exchange for activation. Pan-KRAS inhibitors, such as the one described here, have broad therapeutic implications and merit clinical investigation in patients with KRAS-driven cancers.

## Abstract (web summary)

A non-covalent inhibitor that binds preferentially to the inactive state of KRAS while sparing NRAS and HRAS is reported, indicating that most KRAS oncoproteins cycle between an active state and an inactive state in cancer cells.

## Main

KRAS mutations are among the most frequent gain-of-function alterations found in patients with cancer and their therapeutic targeting has long been a key objective in precision oncology^8–12^. The KRAS GTPase cycles between an active (GTP-bound) and an inactive (GDP-bound) state. GTP hydrolysis is enhanced by GTPase-activating proteins^13^, whereas GDP to GTP exchange is enhanced by guanine nucleotide exchange factors^14^. Allele-specific inhibitors, which bind covalently to KRAS G12C and trap it in an inactive state^2,15^, have demonstrated clinical benefits in patients with lung cancer^5–7^. These drugs, however, require a reactive cysteine residue for inhibition and cannot be used against non-G12C mutants, which constitute most KRAS alterations in cancer. As such, efforts to identify therapeutics that enable broad inhibition of KRAS mutants are continuing. Moreover, the most prevalent of non-G12C KRAS mutants found in cancer are thought to be deficient in GAP-assisted GTP hydrolysis^16–21^ and exist in a non-excitable or constitutively active state. As such, it is not clear whether the inactive-state selective trapping mechanism afforded by covalent G12Ci (refs. ^2,22,23^), will work against non-G12C mutants.

**Fig. 1:**
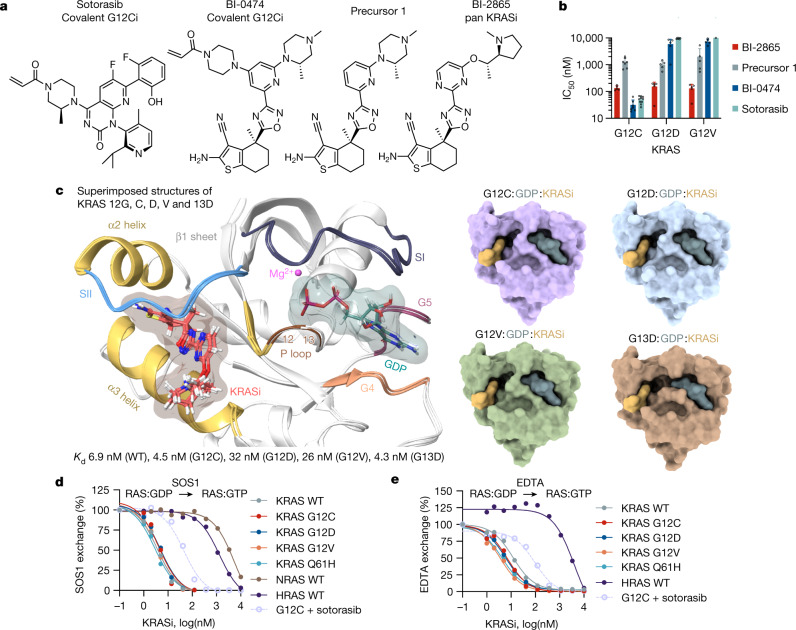
Identification of a non-covalent inhibitor that inactivates common cancer-causing KRAS mutants. **a**, Chemical structures of the indicated compounds. **b**, Isogenic BaF3 cells expressing the indicated KRAS mutants were treated for 5 days in the absence of IL3 (oncogene dependent growth) to determine the effect on proliferation (mean ± s.e.m., *n* = 5, unless otherwise indicated, *n* denotes biological replicates). **c**, Cocrystal structures of drug-bound WT, G12C, G12D, G12V and G13D mutant KRAS. **d**,**e**, The effect of KRASi treatment on nucleotide exchange stimulated either by SOS1 (**d**) or EDTA (**e**). The reaction of KRAS G12C with the covalent inhibitor sotorasib is shown for comparison. A representative of two independent repeats is shown in **d** and **e**. Source data

**Extended Data Fig. 1:**
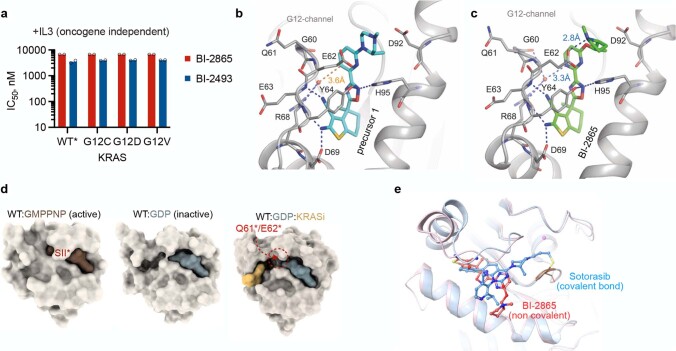
Rational design of a non-covalent pan KRAS inhibitor. **a**, Isogenic BaF3 cells engineered to express the indicated KRAS variants were treated as shown for 72 h to determine the IC_50_ (n = 2, mean and each replicate). IL3 stimulation was used to induce oncogene independent growth. The effect of treatment in the absence of IL3 is shown in Fig. 1b. *: endogenous KRAS WT. **b**,**c**, Co-crystal structures of precursor 1 (b) or BI-2865 (c) bound to KRAS. **d**, Space filling models of KRAS variants in an apo- or drug-bound conformation. S: switch; *: active state conformation. **e**, Superimposition of co-crystal structures of KRAS G12C in complex with the covalent inhibitor sotorasib or the pan KRAS inhibitor BI-2865. Source data

To address these issues, we set out to develop small molecule pan-KRAS inhibitors that do not discriminate between KRAS mutants. We began by removing the covalent warhead from the G12C-selective inhibitor BI-0474 (Fig. 1a) and applied structure-based design optimizations to obtain a potent non-covalent inhibitory activity. The latter was assayed by determining the effect on the proliferation of isogenic BaF3 cells engineered to express G12C, G12D or G12V mutant KRAS (Fig. 1b and Extended Data Fig. 1a; a broader analysis across other KRAS mutants is shown below). Although the removal of the covalent warhead (precursor 1) greatly reduced the potency of G12C inhibition, we were surprised to find that the warhead-free scaffold indiscriminately inhibited proliferation driven by the noted KRAS variants, albeit with a low potency (half-maximum inhibitory concentration (IC_50_) ≥ 1 µM). The crystal structure of precursor 1 in complex with wild-type (WT) KRAS (Extended Data Fig. 1b) showed that the piperazine moiety was suboptimally positioned between the carboxylates of E62 in the Switch II motif and D92 in the α3 helix of KRAS. Furthermore, we observed a highly coordinated water molecule near the C5 atom of the pyridine ring (3.6 Å). We thus proposed that the potency of precursor 1 could be enhanced by improving its interaction with E62 and by introducing an acceptor functionality at C5. Extensive optimization led to BI-2865, a derivative with a prolinol substituent and a pyrimidine linker, which enabled a direct ionic interaction with E62, as well as a water-mediated hydrogen bond network with the side chain of R68 and the main chain carbonyl of Q61 (Extended Data Fig. 1c and below). As a result, BI-2865 inhibited the proliferation of isogenic G12C, G12D or G12V mutant KRAS expressing BaF3 cells more potently than precursor 1 with a mean IC_50_ of roughly 140 nM (Fig. 1b). In KRAS G12C-expressing BaF3 cells, the effect of BI-2865, a compound lacking the Michael acceptor needed for covalent bonding, was comparable to that of covalent KRAS G12C inhibitors BI-0474 and sotorasib. Little if any antiproliferative effect was observed when BaF3 cells were treated in the presence of IL3, a condition that stimulates oncogene-independent growth in this system (Extended Data Fig. 1a). Encouraged by these observations we decided to study further the effects of BI-2865 and its related compounds, referring to them as pan-KRAS inhibitors or KRASi.

We established high-resolution cocrystal structures (1.0–1.1 Å) of the pan-KRASi (BI-2865) in complex with several KRAS variants (Fig. 1c, Extended Data Fig. 1d and Extended Data Table 1). These showed a conserved binding pocket across variants (Fig. 1c and Extended Data Fig. 1c) defined by the α2 and α3 helices, the distal portion of the β1 sheet and the distal portion of the Switch II motif in KRAS (Fig. 1c). A part of the KRASi pocket overlapped with that occupied by covalent G12C inhibitors, such as sotorasib and adagrasib, with the notable difference that the pan-KRASi engaged the pocket without relying on a covalent tether and did not extend in the P-loop channel near the G12 residue (Extended Data Fig. 1c,e).

**Extended Data Fig. 2:**
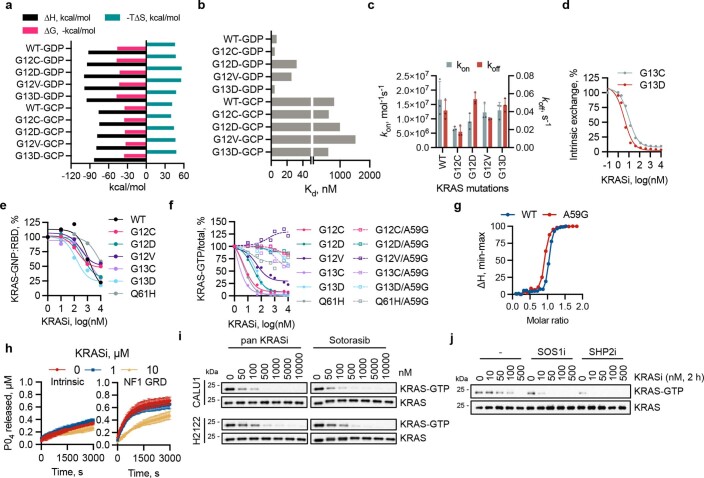
Biochemical and cellular effects of the KRAS inhibitor. **a**,**b**, KRAS variants were loaded with GDP or GCP and subjected to isothermal titration calorimetry (ITC) with the KRASi. **c**, The association (on) and dissociation (off) rate constants of drug binding to GDP-loaded KRAS variants (mean ± s.e.m., n = 3). **d**, Effect of KRASi treatment on nucleotide exchange under intrinsic conditions. **e**, KRAS variants were loaded with the non-hydrolyzable GTP analogue GMPPNP (GNP) and reacted with a GST-tagged RBD domain of CRAF. The mixtures were analyzed by GST pull-down and immunoblotting to determine the ability of the drug to displace effectors from the active state of KRAS. Pull-down reactions were analyzed by immunoblotting and quantified by densitometry. **f**, Extracts from HEK293 cells expressing the indicated variants treated with the KRASi for 2 h were subjected to RBD pull down and immunoblotting. The levels of total and GTP-bound KRAS were quantified by densitometry. **g**, The indicated KRAS variants were loaded with GDP and subjected to ITC in the presence of increasing concentrations of the KRASi. **h**, Effect of KRASi treatment on GTP hydrolysis by KRAS under intrinsic conditions or in the presence of the GAP related domain (GRD) of NF1 (mean ± s.e.m., n = 3). **i**, The indicated KRAS G12C mutant cell lines were treated for 2 h and their extracts were subjected to RBD pull-down and immunoblotting to determine the level of active KRAS. **j**, H2122 cells were treated as shown and analyzed to determine the effect on KRAS activation. A representative of two independent repeats is shown in a, b, d-f, i and j. Source data

The inhibitor bound to the GDP-loaded state of WT, G12C, G12D, G12V and G13D KRAS with high affinity (dissociation constant (*K*_d_), 10–40 nM; Fig. 1c), as determined by isothermal titration calorimetry (ITC) (Extended Data Fig. 2a,b). By comparison, the affinity was 60–140 times lower for KRAS variants loaded with the GTP analogue GCP. Selectivity for the inactive state is probably conferred by the conformation of the Switch II motif (including residues Q61 and E62), which partly occupies the drug pocket in the active state conformation of KRAS (Extended Data Fig. 1d). Surface plasmon resonance studies showed reversible drug-binding kinetics with a dissociation rate (*k*_off_) ranging between 0.015 and 0.05 s^−1^ across KRAS variants (Extended Data Fig. 2c).

Drug-bound, GDP-loaded KRAS variants had diminished activation by nucleotide exchange, either when the latter was stimulated by the nucleotide exchange factor SOS1 (Fig. 1d) or by EDTA (Fig. 1e), as well as under intrinsic conditions (Extended Data Fig. 2d). To determine whether low-affinity binding to the active state also inhibited KRAS function, we tested the ability of the inhibitor to displace the RAS-binding domain (RBD) of CRAF from purified GMPPNP-loaded KRAS variants (Extended Data Fig. 2e). The drug prevented effector binding to active KRAS only at high concentrations (mean IC_50_ roughly 5.5 µM). By contrast, the inhibitory effect of inactive state binding on nucleotide exchange had an IC_50_ of roughly 5 nM. To determine the propensity of the pan-KRASi to target the active state of KRAS mutants in cells, we relied on the transition state mutation A59G, which impairs GTP hydrolysis^2,24^. Introducing the A59G mutation alongside G12, G13 or Q61 KRAS mutants led to a near complete loss of target inhibition (Extended Data Fig. 2f). The affinity of the A59G mutant for BI-2865 was similar to that of WT KRAS (Extended Data Fig. 2g). In addition, low-affinity drug binding to the active state did not result in enhanced KRAS-GTP hydrolysis (Extended Data Fig. 2h). Together, the data indicate that the drug’s inhibitory activity in cells relies predominantly on targeting the inactive or GDP-bound state of KRAS.

**Fig. 2:**
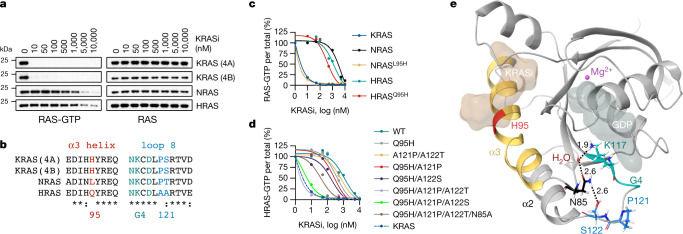
Limited evolutionary divergence confers selectivity for KRAS. **a**, RASless mouse embryonic fibroblasts expressing the indicated RAS isoforms were treated for 2 h. Cell extracts were subjected to RBD pull down and immunoblotting to determine the amount of active RAS. **b**, Sequence alignment of the G domain of RAS isoforms. **c**,**d**, Effect of isoform mimetic substitutions on RAS inhibition: RAS-GTP (**c**) and HRAS-GTP (**d**). HEK293 cells expressing the indicated mutants were treated for 2 h to determine the effect on RAS activation by using RBD pull down, immunoblotting and densitometry. **e**, Cocrystal structure of drug-bound KRAS showing H bonds between S122, N85 and K117 (black dotted lines, distance in Å). A representative of two independent experiments is shown in **a**, **c** and **d**. Source data

**Extended Data Fig. 3:**
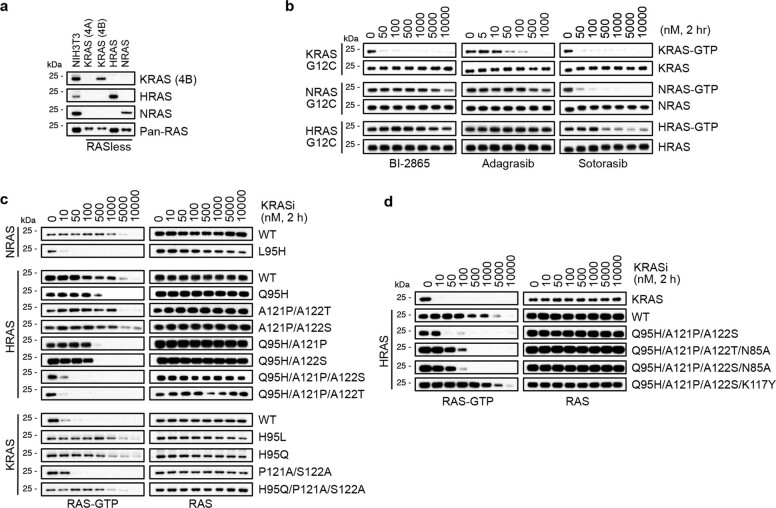
Orthosteric and allosteric effects determine KRAS selectivity. **a**, Extracts from RASless MEFs expressing the indicated RAS isoforms were evaluated by immunoblotting. **b**, HEK293 cells expressing the indicated G12C mutants were treated for 2 h. Cell extracts were subjected to RBD-pull down and immunoblotting to determine the level of active RAS. **c**,**d**, Effect of isoform mimetic substitutions on RAS inhibition. HEK293 cells expressing the indicated mutants were treated as shown for 2 h. Cell extracts were subjected to RBD-pull down and immunoblotting to determine the level of active RAS. The effect of H95 mutants is shown in c and the effects of P121, S122, N85 and K117 mutants are shown in c and d. A representative of two independent repeats is shown in a, b, c and d. Source data

The ability of the inhibitor to suppress nucleotide exchange by HRAS or NRAS was several orders of magnitude less than that for KRAS (Fig. 1d,e). To test whether the inhibitor was selective for KRAS in cells, we used ‘RASless’ murine embryonic fibroblasts (MEFs)^25^ engineered to express only a single RAS variant (Extended Data Fig. 3a). The drug inhibited the activation of KRAS splice variants 4A and 4B with an IC_50_ of less than 10 nM (Fig. 2a). By comparison the IC_50_ for NRAS and HRAS ranged from 5 to 10 µM. The pan-KRAS inhibitor suppressed the cellular activation of KRAS with a similar potency to that observed for the covalent KRAS G12C inhibitor sotorasib (Extended Data Figs. 2i and 3b). Together, the data argue that the drug is a pan-KRAS, inactive state selective inhibitor.

Evolutionary divergence in the GTPase (G) domain of RAS isoforms is minimal, with only few non-homologous amino acids present. As such, the ability of the drug to inhibit only a single RAS isoform was not expected a priori. To determine the basis for KRAS selectivity we interrogated amino acid differences between HRAS, NRAS and KRAS (Fig. 2b). Most notable was the substitution of H95 in the α3 helix of KRAS with L and Q in NRAS and HRAS, respectively. KRAS mimetic substitution at the 95th residue in NRAS (that is, L95H) rendered NRAS almost as sensitive as KRAS to the effect of the drug (Fig. 2c). By comparison, the Q95H substitution in HRAS had only a partial effect, suggesting that this isoform has further selectivity constraints.

Residues 121 and 122 distinguish HRAS from NRAS and KRAS (Fig. 2b, AA, PT and PS, respectively). Given the divergent effects that KRAS mimetic substitutions at the 95th residue had in NRAS and HRAS, we next tested the possibility that residues 121 and 122 allosterically regulate selective inhibition. As shown in Fig. 2d and Extended Data Fig. 3d, KRAS mimetic substitutions at these positions (that is, A121P and A122S (A121P/A122S)) did not have a sensitizing effect on HRAS. Similarly, the KRAS mimetic substitutions Q95H and A121P (Q95H/A121P) or Q95H and A122S (Q95H/A122S) led to only weak sensitization. By contrast, the triple substitution in HRAS (that is, Q95H, A121P  and A122S (Q95H/A121P/A122S)) lead to a similar magnitude of inhibition to that observed in KRAS (Fig. 2d, green line versus rest). As expected, reciprocal H/NRAS mimetic substitutions in KRAS led to attenuated inhibition (Extended Data Fig. 3c). On closer inspection, the cocrystal structure of drug-bound KRAS-GDP suggested contacts between P121, S122 and N85 (Fig. 2e), a residue residing just proximal to the α3 helix and adjacent to the K117 residue in the G4 motif (see below for why proximity to G4 residues was thought to be important). We thus hypothesized that the allosteric effect of P121 and S122 is modulated at least in part by N85. Indeed, introducing the N85A mutation attenuated the effect of the KRASi on HRAS Q95H/A121P/A122S (Fig. 2d, brown versus green line and Extended Data Fig. 3d). The data therefore indicate that three residues with evolutionary divergence in the G domains of RAS isoforms also impose selectivity constraints on KRAS inhibition. The latter is because of a combination of orthosteric effects by H95 in the α3 helix and allosteric (or indirect) effects by P121 and S122 in loop 8.

**Fig. 3:**
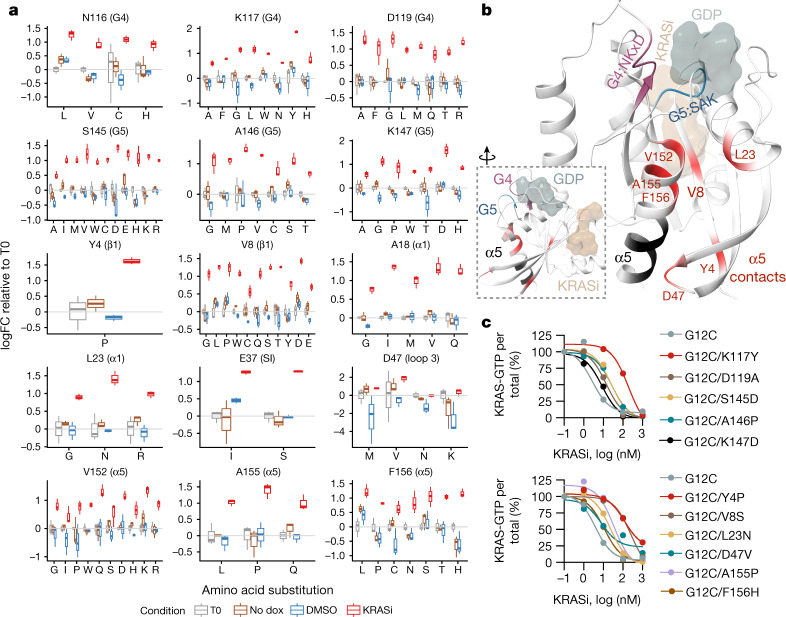
Diverse allosteric effects on inactive state selective KRAS inhibition. **a**, H358 cells were infected with a dox-inducible saturation mutagenesis library based on a KRAS G12C backbone and treated with either DMSO or KRASi for 2 weeks. The selection of mutations in amino acids far from drug-binding interface are shown (median, interquartile range and Tukey whiskers, *n* = 3). FC, fold change; T0, time 0. **b**, Residues from a were mapped in the cocrystal structure of KRAS G12C with the KRAS inhibitor. Residues involved in α5 contacts that were not identified in the screen are shown in black. Inset, 90° rotation. **c**, HEK293 cells expressing KRAS with a single G12C mutation or double mutants involving substitutions in the G4 and G5 motifs (top) or α5 helix contacts (bottom) were treated as shown for 2 h. Cell extracts were subjected to RBD pull down, immunoblotting and densitometry to determine the effect on KRAS-GTP concentrations. A representative of two independent repeats is shown in **c**. Source data

**Extended Data Fig. 4:**
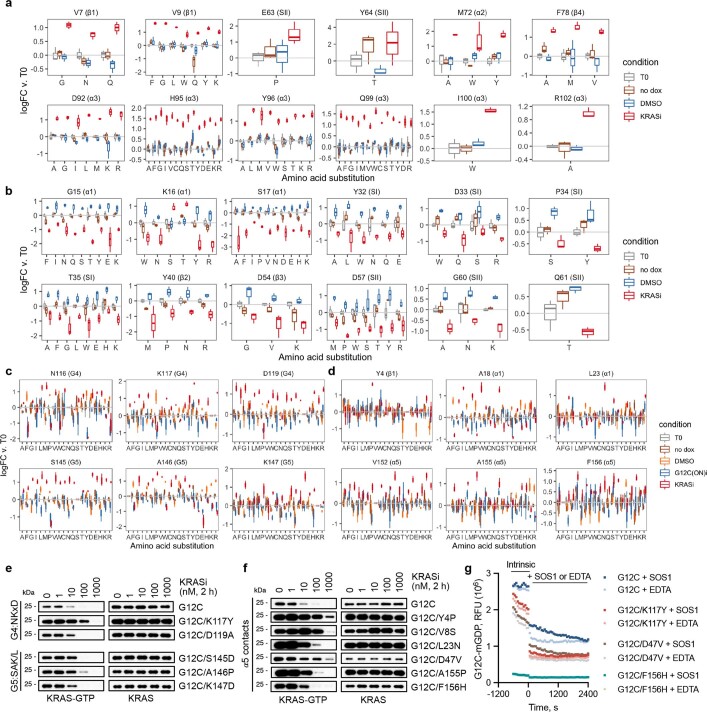
Saturation mutagenesis screen reveals residues that modulate inhibition. **a**,**b**, The effect of substitutions in amino acids involved in the drug binding interface (a) or in the RAS-GDP:SOS1 interface (b) are shown (mean ± 95%CI, n = 3). Only variants with a log FC (KRASi/DMSO) > 1 and FDR < 0.05 are included. All boxplots denote median, interquartile range and Tukey whiskers. **c**,**d**, As in a, but the cells were treated either with the inactive state selective pan KRASi or with an active state selective KRAS G12C inhibitor. The effect of substitutions in amino acids comprising the G4 and G5 motifs or substitutions in amino acids involved in contacts with the α5 helix are shown in c and d, respectively (mean ± 95%CI, n = 3). **e**,**f**, Cell extracts from HEK293 cells expressing the indicated KRAS variants were subjected to RBD-pulldown and immunoblotting to determine the effect on KRAS-GTP. **g**, The indicated KRAS mutants were loaded with mant-GDP and subjected to nucleotide exchange in the absence or the presence of the catalytic subunit of SOS1 or EDTA. A representative of two independent repeats is shown in e, f and g. Source data

We next sought to determine in an unbiased manner the presence of other amino acids that allosterically modulate inactive state selective KRAS inhibition. To this end, KRAS mutant cells were infected with a doxycycline (dox)-inducible complementary DNA library, encoding for all possible amino acid substitutions in the KRAS G12C backbone, followed by treatment with either dimethylsulfoxide (DMSO) or KRASi for 2 weeks in the presence or absence of dox. Variants with statistically significant changes are shown in Fig. 3a and Extended Data Fig. 4. As expected from the findings above and previous studies with covalent G12C inhibitors^26,27^, mutations in residues defining the drug-binding pocket (Extended Data Fig. 4a) led to resistance and were positively selected by treatment. By comparison, mutations in residues at the interface of RAS-GDP and the catalytic subunit of SOS1 were negatively selected by treatment (Extended Data Fig. 4b). The latter suggests that blockade of nucleotide exchange enhances the effect of treatment and is consistent with the inhibitory mechanism of the pan-KRASi (when considering that enhanced nucleotide exchange would diminish the proportion of GDP-bound KRAS—the conformation preferred by the drug). Indeed, pharmacologic inactivation of nucleotide exchange by SOS1 or SHP2 inhibitors led to a more pronounced suppression of KRAS-GTP concentrations by the KRAS inhibitor (Extended Data Fig. 2j).

Mutations in several amino acids residing away from the drug-binding interface were also enriched with treatment (Fig. 3a,b). These included alterations in the G4 (NKxD) and G5 (SAK/L) motifs of the GTPase, as well as several residues that formed contacts with the α5 helix in the cocrystal structure of drug-bound KRAS G12C (hereafter referred to as α5 contacts). A similar saturation mutagenesis screen with an inhibitor targeting the GTP-bound state of KRAS G12C (ref. ^28^) did not identify these variants as being positively selected by treatment (Extended Data Fig. 4c,d), suggesting that they are specifically involved in modulating the inactive state selective inhibition of mutant KRAS. Introducing G4, G5 and α5 (G4/G5/α5) contact mutations alongside G12C led to diminished inhibition of KRAS-GTP concentrations after 2 h of drug treatment (Extended Data Fig. 4e,f and Fig. 3c). The G4/G5/α5 double mutants also had a higher baseline KRAS activation than the G12C single mutant (Extended Data Fig. 4e,f: compare lane 1 across rows). Three representative double mutants, G12C/K117Y, G12C/D47V and G12C/F156H, had a greater ability to undergo nucleotide exchange than KRAS G12C, either in the absence (intrinsic conditions) or the presence of SOS1 or EDTA (Extended Data Fig. 4g). The latter is in agreement with studies showing that naturally occurring mutations in G4 or G5 residues enhance intrinsic or SOS1-mediated nucleotide exchange^29^. Together, the data indicate that the allosteric effect of G4/G5/α5 residues on KRAS inhibition is mediated, at least in part, by modulating the dynamics of the KRAS nucleotide cycle. In agreement, the K117Y mutation reversed the effect that KRAS mimetic substitutions in HRAS had on KRASi treatment (Extended Data Fig. 3d). The latter supports the importance of the P121/S122-N85-K117 interaction network on selective KRAS inhibition.

**Fig. 4:**
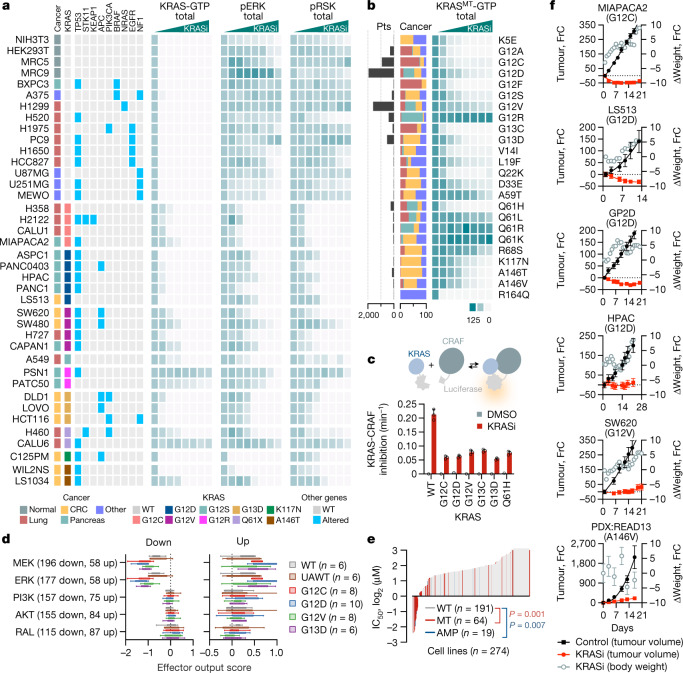
Selective inhibition of oncogenic signalling and KRAS-driven tumour growth. **a**, Thirty-nine cell lines were treated for 2 h to determine the effect on KRAS activation and downstream signalling. **b**, HEK293 cells expressing the indicated KRAS mutants were treated as shown and analysed to determine the effect on KRAS activation. The abundance (Pts) and distribution of mutations across cancer types (Cancer, %) are shown. **c**, A split luciferase assay was used to determine the rate constant for the inhibition of the KRAS–CRAF interaction by treatment in live cells (mean ± s.e.m., *n* = 3). **d**, Effect of KRASi treatment on the transcriptional output by key RAS effector pathways (median, interquartile range and Tukey whiskers). The number of effector-dependent genes used to calculate the output score is shown in parentheses. **e**, Profiling of IC_50_s in a panel of 274 cell lines. **f**, Mice bearing xenograft models were treated to determine the effect on tumour growth and animal weight (mean ± s.e.m., *n* = 5). FrC, fractional change (%). Source data

**Extended Data Fig. 5:**
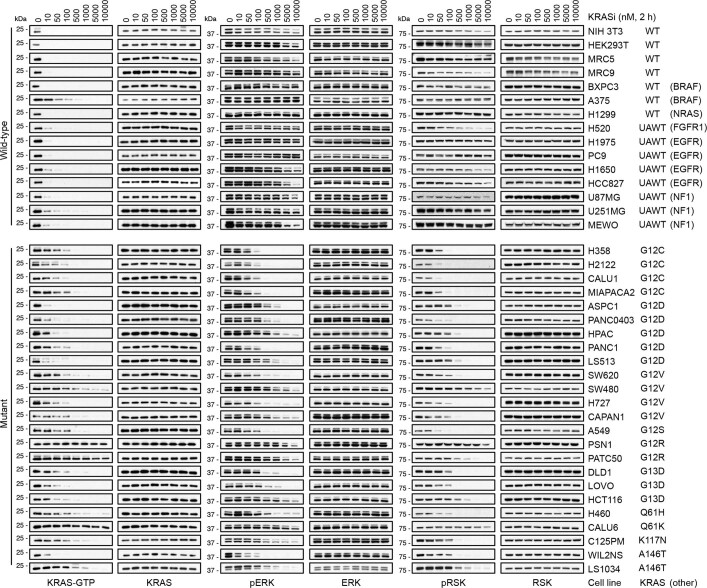
Selective inhibition of oncogenic signaling driven by various KRAS mutants. Extracts from cell lines treated with the KRAS inhibitor were subjected to RBD pull-down and immunoblotting to determine the effect on KRAS and ERK signaling activation. WT: wild-type, UAWT: upstream activated wild-type. Co-occurring alterations in select other genes are shown. The data in Fig. 4a are densitometric quantifications of the immunoblots shown here.

**Extended Data Fig. 6:**
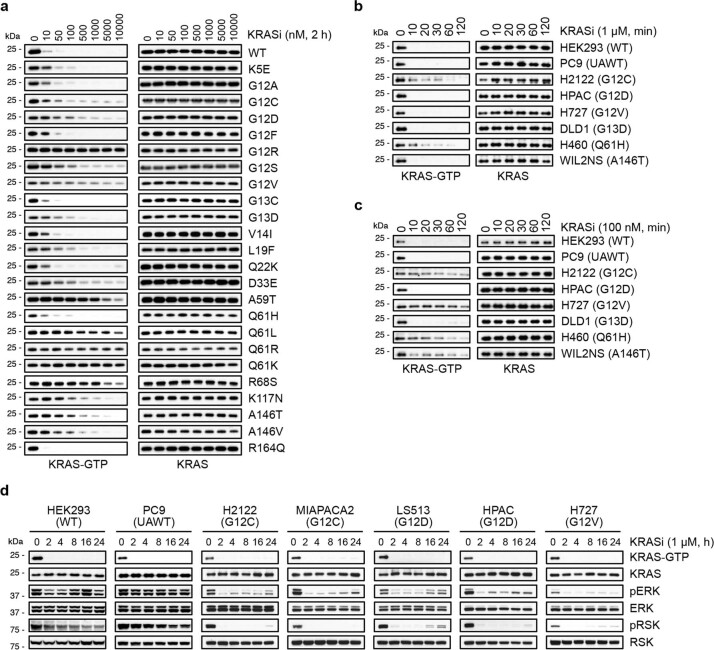
Rapid and sustained inactivation of common KRAS mutants. **a**, HEK293 cells expressing the indicated KRAS variants were treated as shown and analyzed to determine the effect on KRAS activation. **b**–**d**, The indicated cell lines were treated over time to determine the effect on KRAS activation (b–d) and ERK signaling intermediates (d). The cells in b and c were treated in serum-free medium, whereas those in a and d were treated in complete medium. A representative of two independent repeats is shown in a–d.

We next evaluated the ability of the pan-KRASi to suppress KRAS activation and downstream signalling in a panel of 39 cell lines (Fig. 4a), originating from lung, colorectal or pancreatic cancers. Seven cell lines harboured WT KRAS (WT group). Eight cell lines had alterations that activate upstream signalling (upstream activated WT (UAWT) group). Twenty-four cell lines had KRAS mutations (G12C: *n* = 4, G12D: *n* = 5, G12V: *n* = 4, G12S: *n* = 1, G12R: *n* = 2, G13D: *n* = 3, Q61X: *n* = 2, K117N: *n* = 1, A146T: *n* = 2). As expected from the biochemical data, the drug inhibited KRAS activation in both WT and mutant models (Extended Data Fig. 5 and Fig. 4a). The inhibitor also inactivated 18 out of the 24 most common KRAS mutants found in cancer, when the latter were individually expressed in human embryonic kidney 293 (HEK293) cells (Fig. 4b and Extended Data Fig. 6a).

Considering that the KRASi binds to WT and mutant KRAS with a similar affinity (*K*_d_ in Fig. 1c), the rate of inhibition may be used to compare the relative propensities of KRAS variants to undergo nucleotide cycling in cells. A split luciferase biosensor^30^ was used to measure the interaction of KRAS with full length CRAF in live cells and to determine the effect of drug treatment over time (Fig. 4c). The drug disrupted the KRAS–CRAF complex with a rate constant of roughly 0.2 min^−1^ for WT KRAS and an average rate constant of 0.05 ± 0.01 min^−1^ for KRAS mutants (G12C/D/V, G13C/D and Q61H). In agreement, target inhibition was less potent in KRAS mutant models (Fig. 4a, mean IC_50_ of roughly 1 nM for WT and roughly 12 nM for mutant models and Extended Data Fig. 6b–d). Together, the data indicate that, rather than being fixed in an active state, common KRAS mutants undergo nucleotide cycling in cancer cells (a property that is necessary for the drug to access the GDP-bound conformation). The data also argue that KRAS mutants cycle with slower kinetics than WT KRAS.

**Extended Data Fig. 7:**
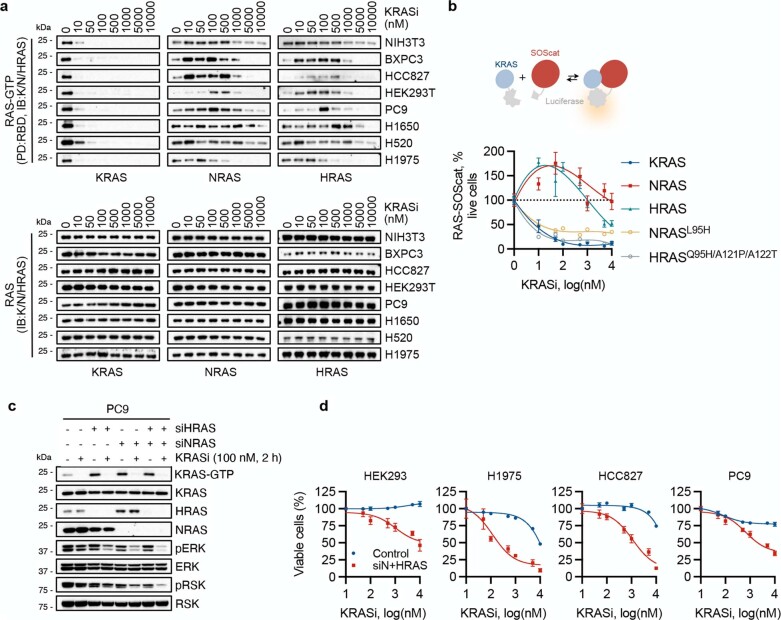
Drug induced cellular activation of HRAS and NRAS. **a**, The indicated KRAS WT cells were treated for 2 h to determine the effect on RAS isoform activation. A representative of two independent repeats is shown. **b**, A split luciferase construct was used to determine the effect of treatment (2 h) on the interaction between RAS variants and the catalytic subunit of SOS1 (SOScat, mean ± s.e.m., n = 3). **c**, The cells were transfected with *HRAS-* and/or *NRAS-*specific siRNA pools and treated as shown. Extracts were analyzed to determine the level of KRAS activation and downstream signaling inhibition. A representative of two independent repeats is shown. **d**, The indicated *KRAS* WT models were transfected with *NRAS*- and *HRAS*-specific siRNA pools, followed by treatment with the KRASi for 72 h. The effect on cell viability was determined by the ATP-glow assay (mean ± s.e.m., n = 3). Source data

KRASi treatment inhibited ERK and RSK phosphorylation predominantly in KRAS mutant models (mean IC_50_ roughly 150 nM for pERK and roughly 70 nM for pRSK), with only a small effect in WT or UAWT models (mean IC_50_ > 10 µM for either intermediate, Fig. 4a and Extended Data Fig. 5). KRASi treatment in the latter models led to an induction of N/HRAS-GTP amounts, an effect that was attenuated at higher drug concentrations (Extended Data Fig. 7a). Pan-KRASi treatment induced the formation of a complex between the catalytic subunit of SOS1 (SOScat) and H/NRAS, while displacing SOScat from KRAS (Extended Data Fig. 7b). Furthermore, the KRASi was able to inhibit ERK signalling and proliferation in KRAS WT cells with small-interfering RNA-mediated down-regulation of N/HRAS (Extended Data Fig. 7c,d). The data thus indicate that the lack of downstream signalling inhibition in KRAS WT models is probably due to compensation by HRAS and NRAS. They also argue for a tight balance and cross-regulation between RAS isoforms with the potential to confer a wide therapeutic index in patients.

**Extended Data Fig. 8:**
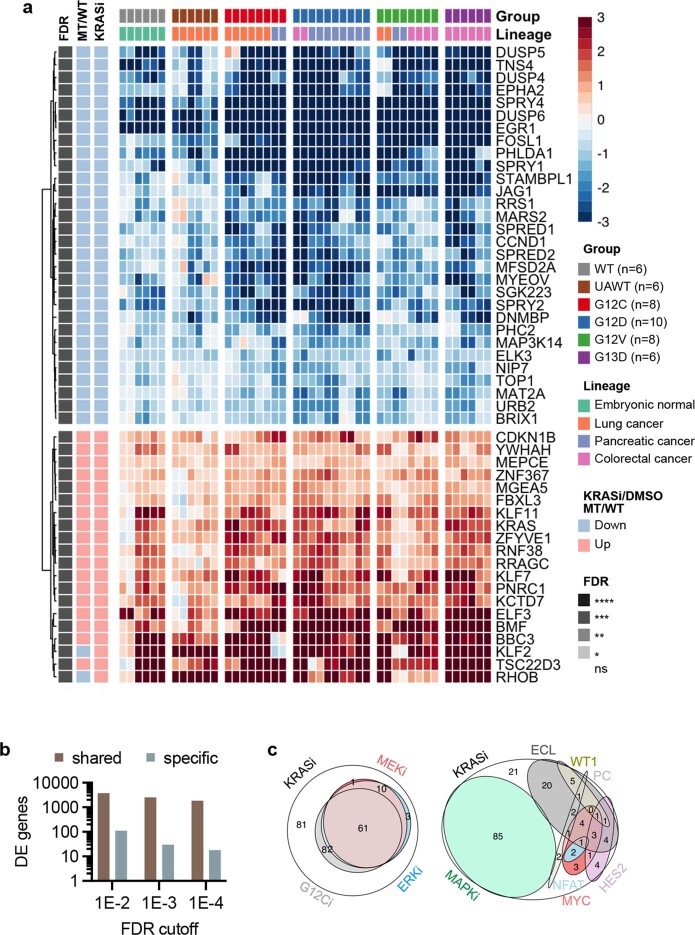
Effect of KRASi treatment on transcriptional output. **a**, Differential expression analysis in models with wild-type or mutant KRAS treated for 2 h with the KRASi. The heat map shows the scaled logFC relative to DMSO. Row annotations show the expression trend in the KRASi/DMSO or MT/WT comparisons. The models are grouped as shown. **b**, The number of differentially expressed (DE) genes shared across groups as compared to those specific for a particular group. **c**, Gene-set enrichment analysis of transcripts down-regulated by treatment. The MAPK-dependent signatures were established experimentally by treating cells with the inhibitors shown (see Methods). The other signatures are from MSigDB. ECL: extracellular ligand; PC: polycomb transcription factors. Source data

To evaluate the effect on downstream signalling more broadly, and to identify transcripts and pathways immediately deregulated following KRAS inactivation, we carried out RNA sequencing (RNA-seq) in 22 models treated with either DMSO or KRASi for 2 h (Extended Data Fig. 8a). Most differentially expressed genes during KRASi treatment were shared among KRAS mutant models, and only a small subset showed a pattern specific for a single mutant (Extended Data Fig. 8b). We next evaluated the effect of KRASi treatment on key RAS effector pathways by relying on experimentally derived transcriptional output signatures (Methods). As shown in Fig. 4d, KRASi treatment suppressed MEK/ERKi down-regulated genes, while inducing MEK/ERKi up-regulated genes. This effect was, again, more pronounced in cells with mutant as compared to WT KRAS (false discovery rate (FDR) <0.001 for down-regulated and FDR < 0.02 for up-regulated genes). KRASi treatment had a very small, if any, effect on PI3K, AKT and/or RAL signalling output in the models examined here (Fig. 4d). The mitogen-activated protein kinase (MAPK)-independent portion of the KRAS transcriptional output overlapped with signalling driven by extracellular ligands (Extended Data Fig. 8c).

**Extended Data Fig. 9:**
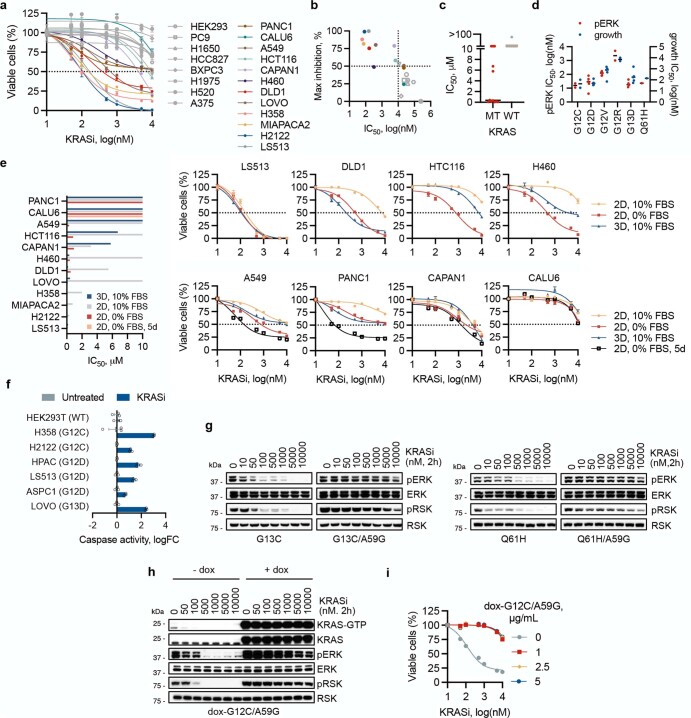
Antiproliferative effect of the KRAS inhibitor. **a**–**c**, The indicated cells were grown in 3D culture and treated with the KRASi for 72 h and the effect on cell viability (A) was determined using the ATP-glow assay (mean ± s.e.m., n = 4). The effect on maximal inhibition as a function of IC_50_ (b) and the difference in IC_50_ between KRAS MT and WT models (c) are shown. **d**, Correlation between ERK and growth inhibition in cell lines with distinct KRAS mutations. **e**, Antiproliferative effect of treatment under the cell culture conditions shown (mean ± s.e.m., n = 4). **f**, The indicated models were treated for 24 h followed by measurement of caspase activation (mean ± s.e.m., n = 3). **g**, Extracts from HEK293 cells expressing the indicated variants were analyzed to determine the effect of treatment on ERK signaling activation. **h**,**i**, H358 cells with dox-inducible expression of KRAS G12C/A59G were analyzed as shown to determine the effect on KRAS signaling (h) or proliferation (i, mean ± s.e.m., n = 3). A representative of two independent repeats is shown in g and h. Source data

The ability of the KRASi to suppress MAPK output more potently in KRAS mutant models correlated, on average, with a more potent antiproliferative effect in a panel of 274 cancer cell lines (Fig. 4e and Extended Data Fig. 9a–c). KRAS amplified cell lines also had on average a lower IC_50_, as compared to models with WT KRAS (Fig. 4e, dedicated manuscript in preparation). The antiproliferative effect of treatment varied in KRAS mutant models, a finding that is in agreement with previous reports suggesting that only some KRAS mutant tumours depend on KRAS for their growth^31,32^. Models harbouring a KRAS G12R or a KRAS Q61L/K/R mutation had little inhibition by the KRASi (Fig. 4a,b and Extended Data Fig. 9d). Furthermore, serum-deprivation enhanced the potency of inhibition (Extended Data Fig. 9e), an observation that is again consistent with the inactive state selective drug trapping mechanism (considering that serum withdrawal diminishes growth factor-driven nucleotide exchange). Drug treatment led to an increase in caspase activation in models harbouring a KRAS mutation but not in cells with WT KRAS (Extended Data Fig. 9f). In agreement with the data above, the inhibitory effect of the pan-KRASi on signalling and proliferation was attenuated in A59G double mutants (Extended Data Fig. 9g–i).

**Extended Data Fig. 10:**
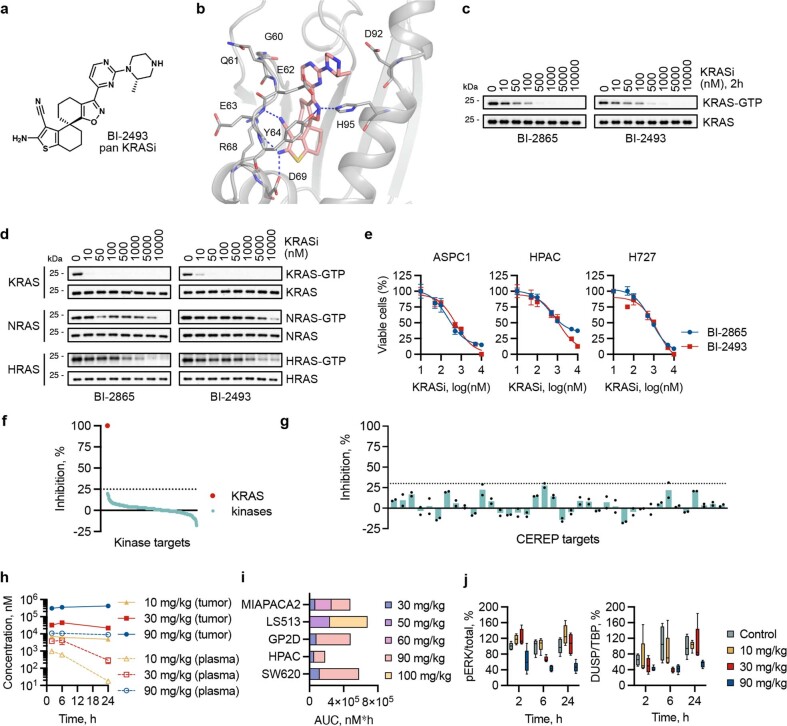
Characterization of the *in vivo* pan KRAS inhibitor BI-2493. **a**, Chemical structure of BI-2493. **b**, Co-crystal structure of KRAS G13D with BI-2493. **c**,**d**, CALU1 cells (c) or RASless MEFs expressing the indicated RAS isoforms (d) were treated as shown and analyzed by RBD pulldown and immunoblotting. A representative of two independent repeats is shown. **e**, The indicated cancer cell lines were treated as shown for 72 h. Viable cells were determined by ATP-glow (mean ± s.e.m., n = 3). **f**,**g**, Effect of KRASi-treatment (1 µM) across a panel of 404 kinases (f) or 38 targets commonly used in safety profiling (g). KRAS was not part of the assay and included only as a reference. **h**–**j**, GP2D (h, i, j) or other (i) xenograft bearing mice were treated with BI-2493 (10–90 mg/kg, p.o. twice a day). Plasma (h, i) or tumors (h, j) from treated mice were used to determine the concentration of BI-2493 or the effect on inhibition of the noted ERK signaling intermediates. h: mean ± s.e.m (n = 4), j: median, 95% confidence interval and range are shown (n = 4). Source data

BI-2493 is a structural analogue of BI-2865 that was optimized for in vivo administration. The two pan-KRASi had a similar binding mode to mutant KRAS (Extended Data Fig. 10a,b) as well as similar inhibitory properties in RASless MEFs and cancer cell lines (Extended Data Fig. 10c–e). BI-2493 was highly selective for KRAS and did not cause more than 30% inhibition in a panel of 404 kinases or 38 targets commonly used in safety profiling (Extended Data Fig. 10f,g, respectively). Last, the inhibitor attenuated tumour growth in mice bearing KRAS G12C, G12D, G12V and A146V mutant models (Fig. 4f), without causing apparent toxicity to the mice (at least as determined by monitoring animal weight). The antitumor effects were associated with favourable pharmacokinetic properties, as evidenced by the amount of drug exposure in both plasma and tumour (Extended Data Fig. 10h,i), as well as a concordant inhibition of ERK phosphorylation and *DUSP6* messenger RNA expression in tumour models (Extended Data Fig. 10j).

Here we report the discovery of a pan-KRAS inhibitor that inactivates common KRAS oncoproteins without needing to be covalently anchored to a specific mutant amino acid (Supplementary Discussion). Selectivity for KRAS was conferred through direct and/or indirect constraints imposed by three G domain residues that show evolutionary divergence between RAS isoforms. The pan-KRAS inhibitor works by preferentially targeting the inactive state of KRAS to prevent its reactivation by nucleotide exchange. In cells with mutant KRAS, this led to suppressed downstream signalling and cancer cell growth, suggesting that common KRAS mutants found in cancer depend on nucleotide exchange for activation. In cells with WT KRAS, drug treatment led to an increase in the activation of other RAS isoforms, which limit the antiproliferative effect of treatment (Extended Data Fig. 7a). The cellular effects of the pan-KRAS inhibitor argue that susceptibility to inactive state selective inhibition is not a unique property of KRAS G12C but one that can be applied to broadly target KRAS mutants. The latter include G12A/C/D/F/V/S, G13C/D, V14I, L19F, Q22K, D33E, Q61H, K117N and A146V/T, which together comprise most of the KRAS mutants found in cancer.

Our study serves as a blueprint for the development of more KRAS directed therapeutics, including small molecule inhibitors of GTP-bound KRAS and proteolysis targeting chimeras. Pan-KRAS inhibitors, such as the one described here, merit clinical testing in patients as they stand to affect the clinical outcomes of patients with KRAS-driven cancers, including those with lung, colorectal and pancreatic cancer as well as further less-frequent cancer types. Selective inhibition of KRAS, while sparing HRAS and NRAS, a property that differentiates our inhibitor from other emerging drugs, is likely to produce a wide therapeutic index in the clinic.

## Methods

### Cell culture and reagents

The cell lines used in the study were maintained in DMEM medium supplemented with 10% FBS, penicillin, streptomycin and 2 mM l-glutamine. Cells were obtained from ATCC and tested negative for mycoplasma. Sotorasib was purchased from Selleckem.

### Immunoblotting

Cells were gathered and lysed with NP40 lysis buffer (50 mM Tris pH 7.5, 1% NP40, 150 mM NaCl, 10% glycerol and 1 mM EDTA) containing protease (Pierce Protease Inhibitor Mini Tablets, Thermo Fisher Scientific no. 88665) and phosphatase (Pierce Phosphatase Inhibitor Mini Tablets, Thermo Fisher Scientific no. 88667) inhibitors on ice for 10 min. After that, the lysates were centrifuged at 16,000*g* for 10 min before protein concentration was quantified by the BCA assay (Thermo Fisher Scientific). The proteins were resolved on 4–12% SDS–PAGE gels (Thermo Fisher Scientific) in 1× MOPS buffer (Thermo Fisher Scientific) at 90–120 constant voltage (V) and transferred to nitrocellulose membranes (GE Healthcare) with 1× Tris-Glycine Buffer (BioRad) at 100 V for 1 h. Membranes were blocked in 5% non-fat milk for 1 h and then probed with primary antibodies overnight at 4 °C and visualized using horseradish peroxidase (HRP)-conjugated secondary antibodies and extracellular ligands (Thermo Fisher Scientific). Primary antibodies used to detect NRAS (sc-519) or HRAS (18295-1-AP) were obtained from Santa Cruz Biotechnology or Proteintech, respectively. Those used for the detection of phospho-ERK (9101), ERK (4696), phospho-RSK(Thr359) (8753), RSK (9355), β-actin (4970) and HA (3724) were obtained from Cell Signaling Technology. Antibodies used to detect KRAS (WH0003845M1) were obtained from Millipore Sigma and the antibody detecting CRAF (610152) was purchased from BD Bioscience. Immunoblots were quantified using ImageJ.

### RAS activation assay

RAS activity was detected using the active Ras pull-down and detection kit (Thermo Fisher Scientific). Briefly, GST–RAF1 RBD and glutathione agarose resin were mixed with whole-cell lysates and incubated on a rotator for 1 h at 4 °C, followed by three washes and elution with 2× SDS–PAGE loading buffer. The samples were then analysed by SDS–PAGE and western blotting with a KRAS-specific antibody (2F2, Sigma). When epitope-tagged KRAS, NRAS and/or HRAS variants were exogenously expressed, an epitope-specific antibody enabled specific determination of these variants in their GTP-bound conformation.

### Protein expression and purification

Each RAS gene (K, N or HRAS) was cloned into pET28a vector with an N-terminal His6 tag. SOScat (566–1049 amino acids (aa)) was cloned into pGEX-4T-1 vector with a N-terminal GST tag. Each gene was expressed in *Escherichia coli* BL21 cells, cultured in Terrific Broth media overnight and induced with 0.5 mM isopropyl-β-d-thiogalactosid at 18 °C. The cells were lysed in binding buffer (50 mM Tris-HCl pH 7.5, 0.25 M NaCl, 10% glycerol, 10 mM imidazole, 1 mM benzamidine, 1 mM phenylmethylsulfonyl fluoride, 5 mM β-mercaptoenthanol) and the extracts were subjected to affinity purification by using nickel-nitrilotriacetic acid (Gold Bio) or a glutathione column (GE healthcare). His-tagged proteins were eluted in 250 mM imidazole and GST-tagged proteins were eluted in 25 mM reduced glutathione (pH 8.8). Eluted fractions were subjected to a second round of purification by size-exclusion chromatography by using a Sephacryl 200 size-exclusion column (Cytiva) in a buffer containing 25 mm Tris-HCl, pH 7.5, 150 mm NaCl, 1 mm dithiothreitol and 5% glycerol.

### Nucleotide exchange

Nucleotide exchange was measured through exchange of GDP to GTP-DY-647P1 by using a Homogeneous Time Resolved Fluorescence assay (Ex/Em: 337/665; 620) in PHERAstar (BMG Labtech). GST-tagged RAS was mixed with α-GST-Tb antibody (1.5× solution) and a 10 µl sample was delivered to reaction wells. The inhibitors were tested in ten different concentrations with threefold serial dilution from 10 μM, and were delivered to reaction wells using an acoustic dispenser (Echo, Labcyte). The RAS/GST-Tb antibody and inhibitor mixture was pre-incubated for 1 h at room temperature before reaction. Then, 5 µl of GTP-DY-647P1 (final 0.15 µM) and SOScat (564–1049 aa) were added to reaction well to initiate the exchange reaction. In each reaction, 10–30 nM of RAS and 5–150 nM of SOScat were used.

### ITC

Calorimetric experiments of the pan-KRASi (BI-2865) were performed on a MicroCal PEAQ-ITC instrument (Malvern Panalytical Ltd). Protein solutions were buffer exchanged by dialysis into buffer containing 20 mM HEPES pH 7.6, 130 mM sodium chloride, 2 mM magnesium chloride and 0.5 mM TCEP. All measurements were carried out at 23 °C. Titrand and titrator concentrations were adjusted to 3% DMSO. The cell was loaded with protein solutions in the range of 0 to 40 μM. All injections were performed using an initial injection of 0.5 μl followed by 19 injections of 2 μl of compound in the range of 100–500 µM. The data were analysed with the MicroCal PEAQ-ITC analysis software package (v.1.1.0.1262). The first data point was excluded from the analysis. Thermodynamic parameters were calculated by the following formula: Δ*G* = Δ*H* − *T*Δ*S* = −*RT*ln*K*_d_, where Δ*G*, Δ*H* and Δ*S* are the changes in free energy, enthalpy and entropy of binding, respectively, *T* is the temperature, and *R* is the universal gas constant (Supplementary Fig. 2).

### Surface plasmon resonance

Surface plasmon resonance experiments were performed on Biacore 8K instruments (Cytiva). Streptavidin (Prospec) was immobilized at 25 °C on CM5 Chips (Cytiva) using 10 mM HBS-P+ buffer (pH 7.4) (Cytiva). The surface was activated using 400 mM 1-ethyl-3-(3-dimethylaminopropyl)-carbodiimide and 100 mM N-hydroxysuccinimide (Cytiva) (contact time 420 s, flow rate 10 ml min^−1^). Streptavidin was diluted to a final concentration of 1 mg ml^−1^ in 10 mM sodium acetate (pH 5.0) and injected for 600 s. The surface was subsequently deactivated by injecting 1 M ethanolamine for 420 s and conditioned by injecting 50 mM NaOH and 1 M NaCl. Dilution of the biotinylated target proteins and streptavidin coupling was performed using running buffer without DMSO. The target proteins were prepared at 0.1 mg ml^−1^ and coupled to a density between 200 and 800 response units. All interaction experiments were performed at 25 °C in running buffer (20 mM Tris(hydroxymethyl)aminomethane, 150 mM potassium chloride, 2 mM magnesium chloride, 2 mM Tris(2-carboxyethyl)phosphine hydrochloride, 0.005% Tween20, 40 μM Guanosine 5′-diphosphate, pH 8.0, 1% DMSO). The compounds were diluted in running buffer and injected over the immobilized target proteins (concentration range for KRAS mutants, 6.25–1,000 nM). Sensorgrams from reference surfaces and blank injections were subtracted from the raw data before data analysis using Biacore Insight software. Affinity and binding kinetic parameters were determined by using a 1/1 interaction model, with a term for mass transport included (Supplementary Fig. 3).

### Protein preparation and crystallization

KRAS WT, G12C, G12D, G12V, G13D and respective biotinylated versions (for each, amino acids 1–169 of UniProt sequence P01116) were cloned, expressed and purified as previously described^33^. Crystals of BI-2865 in complex with the variants above were obtained by cocrystallization. Protein solutions of KRAS WT (42 mg ml^−1^), G12C (38 mg ml^−1^), G12D (40 mg ml^−1^), G12V (48 mg ml^−1^), G13D (41 mg ml^−1^) in 20 mM Tris; 150 mM NaCl; 2 mM TCEP; 2 mM MgCl_2_; pH 7.5 were incubated with 2 mM BI-2865 and 4% DMSO. Crystals were obtained using the hanging drop method, by mixing 1 µl of protein solution with 1 µl of reservoir solution (0.2 mM MgCl_2_, 15–27% PEG 2000, 100 mM sodium acetate pH 4.4) at 4 °C. Plate-like crystals appeared overnight and were flash frozen in liquid nitrogen using 25–30% ethylene glycol in the reservoir as a cryoprotectant. Crystals belonged to same space group P2_1_2_1_2_1_ containing one monomer in the asymmetric unit. Diffraction data was collected at X06SA beamline of the Swiss Light Source (Paul Scherrer Institute). Images were processed with autoPROC^34^ and all structures were solved by molecular replacement using a previously solved structure. Model building and refinement was performed with standard protocols using CCP4, COOT, autoBUSTER v.2.11.2 (http://www.globalphasing.com) and Phenix^35,36^. Data collection and refinement statistics are shown in Extended Data Table 1. The Fo–Fc electron density maps, obtained by a simulated annealing protocol, of the respective structures are shown in Supplementary Fig. 1.

### Cell viability assay and cell proliferation assays

#### Individual cancer cell lines

The cells were seeded in 96-well plates at 2,000 cells per well in triplicates (at the minimum) and treated with the indicated concentrations of BI-2865. After 72 h, cell viability was assayed by CellTiter-Glo Luminescent Cell Viability Assay (Promega). The background value (media without cells) was subtracted from the raw data and fold change was calculated relative to time zero.

#### Isogenic BaF3 cells

In brief, BaF3 cells were transduced with a virus derived from plasmids expressing KRAS G12C, G12D or G12V mutants (pMSCV-KRAS-PGK-Puro-IRES-GFP). The transduction efficacy was monitored by fluorescence-activated cell sorting. Cells were selected in puromycin (1 µg ml^−1^) and Il-3 (10 ng ml^−1^) for 1–2 weeks or until control cells were dead. This was followed by withdrawal of Il-3 and several passages in the absence of Il-3. Integration of the exogenous KRAS was confirmed by sequencing. To determine the effect of drug treatment on proliferation, 1,500 cells were plated in 384-well plates in 60 µl of Roswell Park Memorial Institute medium (10% FCS) and kept overnight at 37 °C. Cell viability was determined as above.

#### High-throughput screen of the 274 cell line panel

This was performed at Horizon Discovery. Briefly, the cells were seeded in 25 μl of growth media in black 384-well tissue culture plates at the density defined for the respective cell line and plates were placed at 37 °C, 5% CO_2_ for 24 h before treatment. At the time of treatment, a set of assay plates (which did not receive treatment) were collected and ATP concentrations were measured by using CellTiter-Glo v.2.0 (Promega) and luminescence reading on an Envision plate reader (Perkin Elmer). BI-2493 (a structurally similar analogue of BI-2865), was transferred to assay plates using an Echo acoustic liquid handling system. Assay plates were incubated with the compound for 5 days and were then analysed by using CellTiter-Glo. All data points were collected by means of automated processes and were subject to quality control and analysed using Horizon’s proprietary software. Horizon uses growth inhibition as a measure of cell growth. The growth inhibition percentages were calculated by applying the following test and equation: if *T* < *V*_0_ then 100 (1 − (*T* − *V*_0_)/*V*_0_) and if *T* ≥ *V*_0_ then 100 (1 − (*T* − *V*_0_)/(*V* − *V*_0_)), where *T* is the signal measure for a test article, *V* is the untreated or vehicle-treated control measure and *V*_0_ is the untreated or vehicle-treated control measure at time zero (colloquially referred to as T0 plates). This formula was derived from the Growth Inhibition (GI) calculation used in the National Cancer Institute’s NCI-60 high-throughput screen.

### Saturation mutagenesis

To generate the saturation mutagenesis library, the DNA sequence of KRAS G12C (codon 2–188) was mutated to encode for all possible amino acids. The DNA sequence was codon-optimized for expression in human cells. The library DNA was then subcloned into the pLIX_403 lentiviral expression vector (Addgene, no. 41395). Lentivirus was produced by transfecting the library DNA along with packaging (psPAX2) and envelope (pMD2.G) plasmid into HEK293T cells. The virus was collected at 48 h after transfection, aliquoted and snap-frozen in liquid nitrogen and stored at −80 °C. The saturation mutagenesis screen was performed in the NCI-H358 cell line (ATCC, CRL-5807). The cells were transduced with the lentivirus library at a multiplicity of infection of 0.5 with polybrene (Millipore) at 0.8 µg ml^−1^. After selection with puromycin (2 µg ml^−1^), live cells were collected and separated into three equal fractions (4 million cells per fraction). One fraction was pelleted and frozen at −80 °C (day 0). Another fraction was propagated in cell culture in the presence of doxycycline (1 µg ml^−1^) and DMSO. The third fraction was propagated in the presence of dox and the pan-KRASi (BI-2865, 10 µM). The cells were passaged when reaching confluence. The media was refreshed every 3 days. The cells were collected and pelleted after 14 days of treatment. The screen was performed in biological triplicates. Genomic DNA from pelleted cells was extracted with the DNeasy blood and tissue kit (Qiagen) and used as template to amplify the mutagenesis library. The following primers were used: forward 5′-tttagtgaaccgtcagatcgcctgg-3′ and reverse 5′-gaaagctgaaccgggatcccgtca-3′. The PCR products were purified using agarose gel electrophoresis and the QIAquick Gel Extraction kit (Qiagen). Purified PCR products were subjected to Nextera reactions according to the Illumina Nextera XT protocol. The samples were indexed and purified with the Agencourt AMPure XP kit before being subjected to HiSeq analysis at 2 × 150 bp. Count files were generated by using the ORFcall software (Broad Institute) and aligned to the KRAS G12C reference sequence. The raw read counts of each treatment groups were analysed using edgeR to determine the log_2_ fold change between the reads at day 14 relative to day 0.

### RNA-seq

HEK293 (WT), MRC5 (WT), MRC9 (WT), PC9 (UAWT), HCC827 (UAWT), H1650 (UAWT), H358 (G12C), H2122 (G12C), CALU1 (G12C), MIAPACA2 (G12C), LS513 (G12D), HPAC (G12D), ASPC1 (G12D), PANC1 (G12D), PANC0403 (G12D), H727 (G12V), CAPAN1 (G12V), SW620 (G12V), SW480 (G12V), LOVO (G13D), DLD1 (G13D) and HCT116 (G13D) cells were treated with the KRAS inhibitor (BI-2865, 5 µM) or DMSO for 2 h in biological duplicates for each condition. RNA was extracted using RNeasy Mini Kit (Qiagen catalogue no. 74104) according to the manufacturer’s instructions. After RiboGreen quantification and quality control by Agilent BioAnalyzer, 500 ng of total RNA per sample underwent polyA selection and TruSeq library preparation according to instructions provided by Illumina (TruSeq Stranded mRNA LT Kit, catalogue no. RS-122-2102), with eight cycles of PCR. Samples were barcoded and run on HiSeq 4000 in a 50/50 bp paired end run, with an average of 30 million paired reads per sample. Ribosomal reads represented less than 0.5% of the total reads generated. The sequencing output files from different lanes were concatenated, aligned to GRCH38 using HISAT2 and transcripts were counted using HTSeq in Python. The count data matrix was then processed by using limma and edgeR in R/Bioconductor, as described. Briefly, the data were filtered by removing transcripts that were not detected in all replicates. Size factor normalization was carried out and differential expression analysis was carried out contrasting each time point to the untreated condition. The count data were transformed to log_2_ counts per million followed by an estimation of the mean-variance relationship. The data for each gene was used to fit a linear model and to compute various statistical parameters for a given set of contrasts. Correction for multiple hypothesis testing was carried out using the FDR method. Differential expression genes were considered those with absolute scaled log_2_ fold change of equal or greater than 2.56 (or more than three standard deviations from the mean) and an adjusted *P* value of less than 0.05. The heat map in Extended Data Fig. 8a shows the top 50 differential expression genes following KRASi treatment while blocking for cell line of origin (that is, shared output). The annotation rows show the trend (up- or down-regulation) in the effect of KRASi over DMSO or in the effect of KRASi in mutant trait over WT models.

#### Effector output score

The output score for the main RAS effector pathways, that is, RAF/MEK/ERK, PI3K/AKT and RAL, H358 (KRAS G12C) mutant cells were treated with DMSO or inhibitors targeting MEK (trametinib, 25 nM), ERK (SCH984, 500 nM), PI3K (BYL719, 1 µM), AKT (MK2206, 1 µM) or RAL (BQU57, 10 µM) for 4 h. RNA extracted from cells subjected to these treatments was sequenced and analysed as described above. Differential expression genes in drug versus DMSO comparisons were those with an absolute scaled logFC of greater than 2.56 and a FDR < 0.05. These genes were then used to determine the effect of KRASi treatment on RAS effector signalling pathways. The output score determined by the average scaled logFC in the KRASi versus DMSO comparison for MEK, ERK, PI3K, AKT or RAL dependent genes. The up- and down-regulated effector output scores were calculated from genes that were, respectively, down- or up-regulated by inhibitors targeting the intermediates and the numbers of genes used to calculate the average score are shown in Fig. 4d. Statistical significance in effector output score in the KRASi versus DMSO comparison were established either by FGSEA or edgeR/camera in R. Statistical significance in the effect of KRASi in mutant trait versus WT models was established using edgeR/roast, also in R.

### Mouse studies

These were carried out as described^37–40^. Mice were were housed according to the internal institutional and Austrian governmental and European Union guidelines (Austrian Animal Protection Laws, ETS-123) at Boehringer Ingelheim or according to the Institutional Animal Care and Use Committee guidelines at MSKCC. All animal studies were approved by the internal ethics and the local governmental committee. To establish cell line-derived xenograft models, 7–8-week-old female NMRI nude (BomTac:NMRI-Foxn1nu) mice with a bodyweight of 20 g from Taconic were engrafted subcutaneously with 5 million (LS513, GP2d, HPAC, SW620) or 10 million cells (MIAPACA2), respectively, suspended in growth factor reduced, phenol red-free Matrigel (Corning) (LS513, GP2d, HPAC, SW620) or in PBS/5% FBS (SW620). Mice were group-housed under pathogen-free and controlled environmental conditions (21 ± 1.5 °C temperature, 55 ± 10% humidity and a 12 h light–dark cycle). Once tumours reached roughly 200 mm^3^ volume, mice were randomized on the basis of tumour size (*n* = 7–8 mice per treatment arm) and treated with drug or vehicle control (0.5% Natrosol/5% HPβCD). The inhibitor used for in vivo studies was a structurally similar analogue of BI-2865 dosed at 90 mg per kg twice daily (BI-2493). Treatment was administered by oral gavage using an application volume of 10 ml per kg and the average tumour diameter (two perpendicular axes of the tumour were measured) was measured in control and treated groups using a calliper in a non-blinded manner by a research technician, who was not aware of the objectives of the study. Data analysis was done by Prism (GraphPad Software). The pan-KRAS inhibitors described here (GDP-KRAS inhibitors) are available as part of a collaborative programme through Boehringer Ingelheim’s open innovation portal opnMe.com: https://opnme.com/collaborate-now/GDP-KRAS-inhibitor-bi-2493.

### Reporting summary

Further information on research design is available in the Nature Portfolio Reporting Summary linked to this article.

## Online content

Any methods, additional references, Nature Portfolio reporting summaries, source data, extended data, supplementary information, acknowledgements, peer review information; details of author contributions and competing interests; and statements of data and code availability are available at 10.1038/s41586-023-06123-3.

### Supplementary information

Supplementary Information This file contains Supplementary Discussion, Synthetic Methods, Figs. 1–4 and Refs.

Reporting Summary

Supplementary Data 1 Raw and processed data from the saturation mutagenesis experiment.

Supplementary Data 2 Raw and processed data from the saturation mutagenesis experiment.

### Source data

Source Data Fig. 1

Source Data Fig. 2

Source Data Fig. 3

Source Data Fig. 4

Source Data Extended Data Fig. 1

Source Data Extended Data Fig. 2

Source Data Extended Data Fig. 3

Source Data Extended Data Fig. 4

Source Data Extended Data Fig. 7

Source Data Extended Data Fig. 8

Source Data Extended Data Fig. 9

Source Data Extended Data Fig. 10

## Figures and Tables

**Extended Data Table 1:**
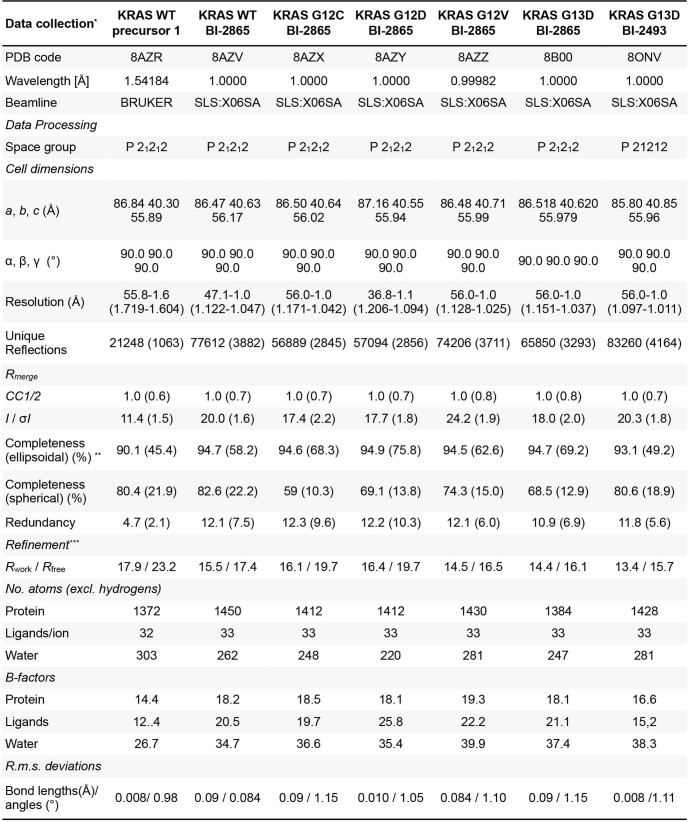
Crystallographic data collection and refinement statistics

* Values in parentheses are for highest-resolution shell.

** Data were scaled anisotropically with Staraniso.

*** Value output by Phenix

## Data Availability

Atomic coordinates and structure factors for the cocrystal structures have been deposited in the Protein Data Bank with accession codes 8AZR, 8AZV, 8AZX, 8AZY, 8AZZ and 8B00. Raw data from sequencing analyses can be found in the supplementary material. Raw files have been deposited in the National Center for Biotechnology Information Gene Expression Omnibus (GSE228010). Source data are provided with this paper.

## Code availability

The data were analysed by using publicly available code.
