# Supplementary material for: Pan-KRAS inhibitor disables oncogenic signalling and tumour growth
Source: Nature. 2023 May 31;619(7968):160–6. doi: 10.1038/s41586-023-06123-3 (PMC10322706; doi:10.1038/s41586-023-06123-3)

nature portfolio

https://doi.org/10.1038/s41586-023-06123-3

# Supplementary information

# Pan-KRAS inhibitor disables oncogenic signalling and tumour growth

In the format provided by the authors and unedited

## Supplementary Discussion

***Drug binding pocket.*** One of the main challenges in the therapeutic targeting of KRAS has been the apparent absence of deep pockets enabling high affinity drug-binding[33,41-46]. Previous efforts have identified chemical moieties that bind to KRAS but almost invariably these led to low potency compounds with limited ability to inactivate KRAS in cells or compounds that lack appropriate target selectivity. The exception has been the recent development of covalent KRAS G12C-selective inhibitors[1-4,22,23]. The latter form a covalent bond with the mutant cysteine at the G12 position, to occupy a previously unappreciated pocket. Recent studies show that covalent tethering can be applied to engage select non-G12C KRAS mutants (such as G12S or G12R)[47,48]. It remains to be seen if these early approaches will yield compounds that potently and selectively inhibit tumor growth. Nevertheless, covalent drugs can only inhibit specific KRAS mutants, thus limiting their therapeutic utility. Here we identify compounds that bind to KRAS with high affinity in the absence of covalent tethering interactions, an approach that paved the way for indiscriminate inhibition of a broad range of cancer-causing KRAS mutations by a single inhibitor. Drug-binding occurred predominantly in the GDP-bound state of KRAS and was optimized through a key interaction between the basic nitrogen of the pyrrolidine ring in the inhibitor and the E62 residue in KRAS. Together the data argue that despite the lack of apparent drug-binding pockets, the surface of KRAS is highly malleable and a binding interface that is conserved across mutants may be induced (or selected) by appropriately designed ligands in the absence of covalent anchors.

***Evolutionary divergence as a constraint for selectivity.*** It is highly surprising that three G-domain residues that differ between RAS isoforms (H95, P121 and S122 on KRAS) confer selectivity to the KRAS inhibitor. Based on its position at the drug-binding interface, we initially suspected that residue 95 would be the main determinant for selectivity. We were puzzled, however, to find that while a KRAS mimetic substitution at the 95th residue sensitized NRAS to the effect of treatment, it had only a minor effect on HRAS. This led us to focus on residues 121 and 122, which distinguish HRAS from NRAS and KRAS. Since these residues reside far from the drug binding pocket, we sought to understand the molecular basis by which they contribute to drug selectivity. In most of the published crystal structures of HRAS, the Switch II motif assumes a closed orientation folding towards the α3 helix. If occurring under physiologic conditions, this would hinder drug binding. We thus considered the possibility that residues 121/122 indirectly modulate the binding pocket. The crystal structure of HRAS A121P/A122S (data not shown)

revealed a Switch II in an open conformation, resembling the conformation of Switch II in KRAS. This suggests either that the orientation of Switch II in HRAS is subject to variation across crystallization conditions or that residues 121 and 122 indirectly module drug binding. To address the latter, we measured the direct interaction of the drug with HRAS variants using ITC. The drug had a low affinity for HRAS WT (Kd: 2.2 µM), a high affinity for HRAS Q95H/A121P/A122S (Kd: 29 nM) and a low affinity for HRAS A121P/A122S (Kd: 2.35 µM). Therefore, the allosteric effect of residues 121/122 does not appear to occur by modulating the drug binding pocket.

The saturation mutagenesis experiments showed that mutations in the G4 motif attenuated the effect of drugs targeting the GDP-bound state with little effect on drugs targeting the GTP-bound state, suggesting a specific role in modulating inactive state selective inhibition. Of the G4 motif residues, K117 stood out given its proximity to residues P121 and S122, as well as its interaction with S122 via N85 in the co-crystal structure of drug bound KRAS and its ability to enhance nucleotide exchange. These observations suggest that the P121/S122-N85-K117 network modulates inactive state selective inhibition through the regulation of the KRAS nucleotide cycle in cells.

***Mechanism of inhibition and implications for KRAS oncoprotein activation.*** The data indicate that the pan KRAS inhibitor works by preferentially binding to the inactive state to prevent nucleotide exchange. Several lines of evidence support this conclusion.

a) While BI-2865 was able to bind to the active state, it did so with a low affinity and little impact on inhibition. The difference in affinity (Kd) ranged between 60- to 140-fold across mutants (favoring the inactive state). More importantly, the inhibitory effect of drug binding to the active state (as determined by displacing RBD from purified GMPPNP-loaded KRAS mutants) had an $IC_{50}$ of ~5 µM. By comparison, the inhibitory effect on the inactive state (as determined by suppressing SOS1 or EDTA nucleotide exchange) had an $IC_{50}$ of ~5 nM (i.e., a 3 log orders of magnitude difference). The cellular inhibition data show that the KRASi suppresses KRAS-GTP levels with a mean $IC_{50}$ of ~12 nM in KRAS mutant models. This value approximates the $IC_{50}$ for biochemical inhibition of nucleotide exchange by inactive state binding and cannot be explained by active state selective inhibition (which was apparent at much higher concentrations).

b) Introducing the A59G mutation alongside KRAS mutants almost completely attenuated inhibition, strongly suggesting that binding to the active state has little effect on cellular inhibition.

Some inhibition (~25%) was apparent at concentrations > 5 µM, which suggest that active state selective inhibition caused by BI-2865 was minor. By comparison, MRTX1133 (an inhibitor that was recently shown to bind both active and inactive forms of G12D) showed more potent displacement of RBD from G12D-GMPPNP and a more potent inhibition of G12D/A59G in cells (data not shown).

c) The lack of significant inhibition of mutants such as KRAS G12R independently supports the predominantly inactive state selective inhibitory mechanism. As noted in more detail below, G12R has been previously reported to lack meaningful GTP hydrolysis capacity. In agreement with the observations above, some activity was again noted at high BI-2865 concentrations, which reflects the relatively minor inhibitory contribution by active state binding.

d) BI-2865 did not increase GTP hydrolysis either under intrinsic conditions or in the presence of NF1. At higher concentrations (10 µM) BI-2865 attenuated NF1-assisted hydrolysis, an observation that is likely caused by displacement of the GAP from binding to the ground state of KRAS (similar to the RBD:KRAS-GMPPNP interaction from above).

e) The saturation mutagenesis screen showed that almost all variants that perturb the interface between RAS-GDP and SOScat were depleted during treatment. Also, the combination of BI-2865 and a SOS1 or a SHP2 inhibitor led to more inhibition of KRAS-GTP levels, a phenomenon that is not expected if BI-2865 was predominantly an active state selective inhibitor. Similarly, were the pan KRASi to preferentially target the active state, then there would be no difference in potency between WT and mutant KRAS, which was not the case from the data presented here.

Together the data suggest that the cellular effects of the pan KRASi are predominantly caused by inactive state selective inhibition. The data also highlight the importance of distinguishing the ability to bind to a particular state from the capacity to achieve inhibition. Taken in isolation, the ability of a compound to binds to the active state, does not suggest that it will inhibit the function of KRAS. The latter effect requires additional experimental evidence. The orthogonal lines of evidence presented here suggest that binding to the active state by BI-2865 has at best a minor inhibitory effect; one that is apparent only at concentrations greater than 5-10 µM. Nevertheless, further chemical optimization of BI-2865 (or similar scaffolds) may give rise to a higher binding affinity for KRAS-GTP, which in turn enables the generation of small molecule, active state selective, pan KRAS inhibitors.

Historically, KRAS mutants (especially G12 or G13 mutants) were thought to be deficient in their ability to hydrolyze GTP and exist in a persistently active state, by virtue of their insensitivity to arginine finger dependent GAPs, such as NF1. However, GTP hydrolysis is required for inactive state selective inhibition because the drug targets the GDP bound conformation, i.e., the product of the hydrolysis reaction. The data presented here suggests that a broad spectrum of KRAS oncoproteins cycle between an active and inactive state in cancer cells and depend on nucleotide exchange for their activation. This is in agreement with recent studies suggesting that KRAS mutants, such as KRAS G12C or KRAS G13D, undergo GAP-assisted GTP hydrolysis in biochemical studies[15,49] and others showing that some KRAS mutants can be indirectly inactivated by suppressing nucleotide exchange or growth factor receptor signaling[50,51].

Inhibition of various KRAS mutants by the pan KRASi, suggests that inactive state selective inhibition is not only effective for KRAS G12C alone, but it can be rather utilized to target a broader range of KRAS mutants in cancer. Such inhibitory mode does not require covalent engagement of the target, as initially suspected by us and others, and it can also be achieved by reversible inhibitors.

***Q61H/K/L/R and G12R mutants.*** Q61 mutants are thought to have a completely impaired GTPase activity, by virtue of not being able to coordinate the water molecule involved in the nucleophilic attack. Based on this, the *a priori* expectation was that none of the Q61 mutants would respond to treatment with an inactive state selective pan KRAS inhibitor. Our data, however, show that the pan KRASi can suppress the activation of KRAS Q61H in cells with either endogenous or exogenous expression of this mutant (which is the most common substitution at the 61 residue). By comparison, little inhibition was observed in cells expressing the much less frequent KRAS Q61K/L/R mutations. Therefore, at least some KRAS Q61 mutants undergo sufficient nucleotide cycling to be susceptible to inhibition by an inactive state selective KRASi.

As noted above, the KRASi did not appear to have a significant inhibitory effect in models harboring a KRAS G12R mutation. The inhibitor, however, bound to GDP-loaded KRAS G12R with a similar $k_{on}$ (1.34E7 $M^{-1}s^{-1}$) and $k_{off}$ (0.03 $s^{-1}$), as those observed for sensitive KRAS variants (data not shown). This suggests that the bulky substitution at the G12 position doesn't preclude the drug from binding. Moreover, KRAS G12R is known to have a severely impaired GTP-hydrolysis rate[52,53]. Therefore, the insensitivity of KRAS G12R further supports the inactive state

selective inhibitory mechanism reported here.

***Implications for cancer therapy.*** Pan KRAS inhibitors stand to impact the clinical outcomes of patients with various types of cancers driven by mutant KRAS, including G12A, G12C, G12D, G12F, G12V, G12S, G13C, G13D, Q61H, K117N and A146T/V. These comprise at least 98% of KRAS mutations found in cancer. KRAS G12D and KRAS G12V are the two most frequently mutated KRAS alleles across all cancers, and the two most common KRAS mutants found in colorectal and pancreatic cancer[51]. Even though they occur less frequently, KRAS G13C/D and A146T/V mutants are common in colorectal cancer[54]. Pan KRAS drugs would also be of prime interest for the treatment of heterogeneous cancers driven by multiple KRAS co-alterations, which occur at an appreciable frequency in treatment-naïve patients or following treatment with covalent G12C inhibitors[40,51]. The inhibitory mechanism described above, coupled with the results of the saturation mutagenesis screen and serum deprivation experiments, suggest that the efficacy of pan KRAS inhibitors could be further enhanced by co-targeting SOS1 or SHP2 to reduce the KRAS-GTP loading of inhibitor-sensitive KRAS mutant alleles.

Drug-free crystal structures of active state KRAS suggest that the Q61 and E62 residues are sub-optimally positioned for drug binding. Nevertheless, binding to the active state could be ‘forced’ with high drug concentrations. The structure of active state KRAS bound to the drug revealed a nearly identical pocket as that observed in the GDP-bound state (data not shown). This suggests that pan KRAS inhibitors could be further optimized to increase their affinity for the active state, providing an avenue to develop small molecule active state selective KRAS inhibitors.

**Synthetic Methods**

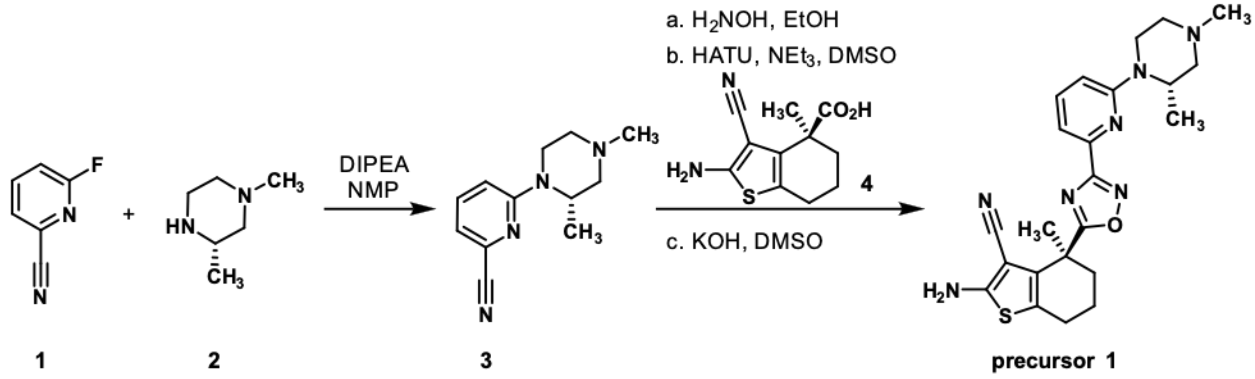

6-[(2S)-2,4-dimethylpiperazin-1-yl]pyridine-2-carbonitrile (**3**):

2-cyano-6-fluoropyridine (150 mg, 1.23 mmol) and (S)-1,3-dimethyl-piperazine (281 mg, 2.46 mmol) were dissolved in NMP (3 ml) and treated with N,N-diisopropylethylamine (0.53 ml, 3.07 mmol). The reaction mixture was heated to 80 °C and stirred overnight. The crude mixture was diluted with MeCN and water and subjected to RP-HPLC which resulted in 181 mg of **3** (68 %yield).

$^1$H NMR (400 MHz, DMSO-$d_6$) δ 7.74 – 7.64 (m, 1H), 7.16 (d, *J* = 7.1 Hz, 1H), 7.12 (d, *J* = 8.9 Hz, 1H), 4.44 (br d, *J* = 4.3 Hz, 1H), 4.02 (br d, *J* = 12.9 Hz, 1H), 3.02 (td, *J* = 12.6, 3.4 Hz, 1H), 2.89 – 2.80 (m, 1H), 2.70 (br d, *J* = 11.2 Hz, 1H), 2.19 (s, 3H), 2.09 (dd, *J* = 11.3, 3.7 Hz, 1H), 1.89 (td, *J* = 11.8, 3.5 Hz, 1H), 1.15 (d, *J* = 6.6 Hz, 3H)

(S)-2-amino-4-(3-(6-((S)-2,4-dimethylpiperazin-1-yl)pyridin-2-yl)-1,2,4-oxadiazol-5-yl)-4-methyl-4,5,6,7-tetrahydrobenzo[b]thiophene-3-carbonitrile (**precursor 1**):

Nitrile 3 (100 mg, 0.46 mmol) was dissolved in EtOH (2.5 ml) and treated with 50% aq. hydroxylamine (57 µl, 0.93 mmol). The reaction mixture was heated to 80 °C for 1 hour. The solvents were removed under reduced pressure and the crude material was subjected to the next reaction. Acid **4** (114 mg, 0.48 mmol) was dissolved in DMSO (1.5 ml), and treated with HATU (184 mg, 0.48 mmol) and triethylamine (0.13 ml, 0.92 mmol) and the reaction mixture was stirred for 15 minutes. The crude hydroxamidine was added and the reaction mixture was stirred overnight. The crude mixture was diluted with MeCN and water and subjected to RP-HPLC which resulted in an acylated hydroxamidine which was not further characterized (78 mg, 36 %). In a next step the material was dissolved in DMSO (1 ml), and treated with powdered KOH (11 mg,

0.20 mmol), and stirred for one hour at ambient temperature. The crude mixture was diluted with MeCN and water and subjected to RP-HPLC which resulted in 33 mg of **precursor 1** (44 %yield).

$^{1}$H NMR (500 MHz, DMSO-d$_6$) δ 7.69 (dd, *J* = 8.7, 7.4 Hz, 1H), 7.27 (d, *J* = 7.3 Hz, 1H), 7.07 (s, 2H), 6.97 (d, *J* = 8.8 Hz, 1H), 4.51 (br s, 1H), 4.14 (br d, *J* = 12.6 Hz, 1H), 3.03 (td, *J* = 12.5, 3.3 Hz, 1H), 2.86 (br d, *J* = 11.0 Hz, 1H), 2.72 (br d, *J* = 11.0 Hz, 1H), 2.61 – 2.53 (m, 2H), 2.20 (s, 3H), 2.12 (dd, *J* = 11.2, 3.6 Hz, 1H), 2.10 – 2.03 (m, 1H), 1.97 – 1.88 (m, 2H), 1.88 – 1.80 (m, 2H), 1.78 (s, 3H), 1.16 (d, *J* = 6.3 Hz, 3H)

$^{13}$C NMR (126 MHz, DMSO-d$_6$) δ 184.17, 168.32, 164.12, 158.39, 144.37, 138.98, 132.06, 119.99, 116.21, 112.68, 109.91, 81.94, 60.17, 55.32, 47.18, 46.56, 39.13, 37.27, 25.26, 24.07, 19.93, 14.35 (one carbon under DMSO peak).

HRMS (ESI) m/z: [M + H]+ Calcd for C23H27N7OS 450.20706; Found 450.20768

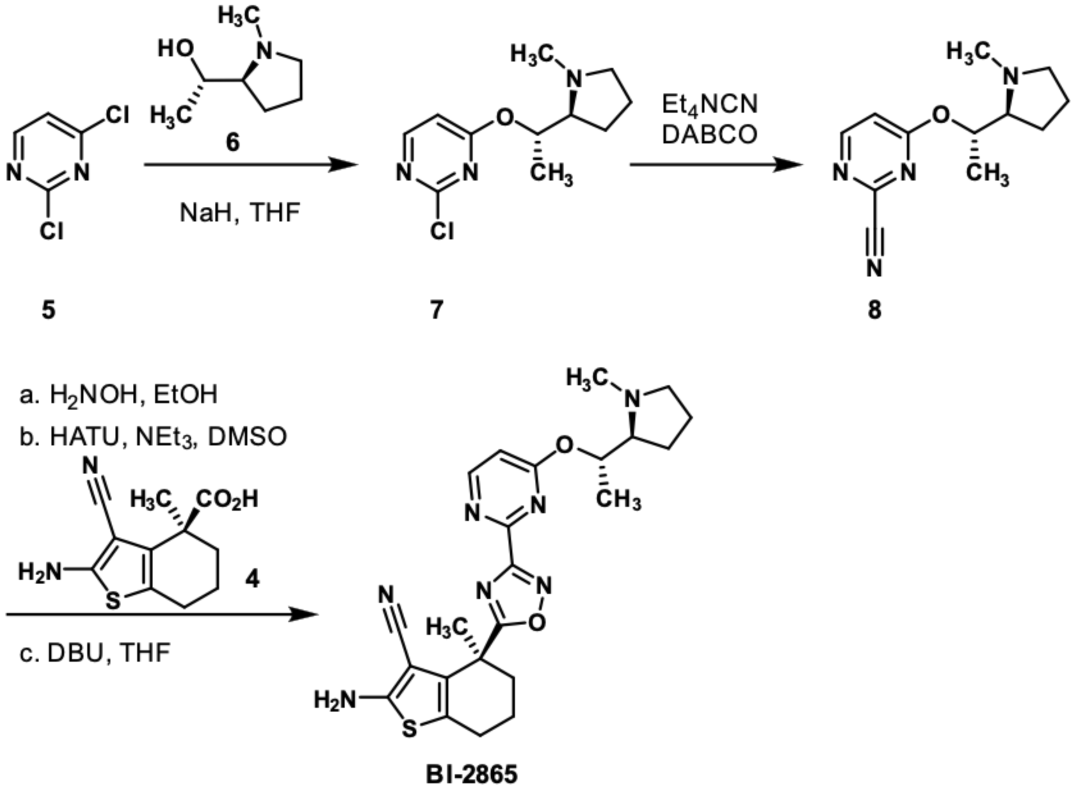


<u>2-Chloro-4-[(S)-1-((S)-1-methyl-pyrrolidin-2-yl)-ethoxy]-pyrimidine (**7**):</u>

A 0 °C cold solution of (S)-1-((S)-1-Methyl-pyrrolidin-2-yl)-ethanol **6** (15.6 g, 120.8 mmol) in THF (150 ml) was treated with sodium hydride (4,83 g, 120,823 mmol) and the reaction mixture was stirred at 0°C for 15 min. To the above reaction mixture 2,4-Dichloro-pyrimidine **5** (15.0 g, 100,686 mmol) was added and the reaction was stirred at rt for 3 hr. The reaction mixture was diluted with ice cooled water (75 ml) and extracted with EtOAc (3 x 50 ml), and the combined organic layers were dried over on anhydrous $Na_2SO_4$ and concentrated under reduced pressure. Purification of the crude material by FCC (EtOAc/hexane) provided the desired product **7** (9.00 g, 37% yield).

$^1$H NMR (500 MHz, CHLOROFORM-d) δ 8.26 (d, *J* = 5.8 Hz, 1H), 6.62 (d, *J* = 5.8 Hz, 1H), 5.31 (quin, *J* = 6.2 Hz, 1H), 3.10 (br t, *J* = 6.6 Hz, 1H), 2.65 – 2.57 (m, 1H), 2.46 (s, 3H), 2.36 – 2.26 (m, 1H), 1.94 – 1.84 (m, 1H), 1.83 – 1.65 (m, 3H), 1.33 (d, *J* = 6.4 Hz, 3H).

<u>4-((S)-1-((S)-1-methylpyrrolidin-2-yl)ethoxy)pyrimidine-2-carbonitrile (**8**):</u>

To a stirred solution of 2-Chloro-4-[(S)-1-((S)-1-methyl-pyrrolidin-2-yl)-ethoxy]-pyrimidine **7** (100,000 mg, 0.41 mmol) was dissolved in dry acetonitrile (3 ml) and treated with tetraethylammonium cyanide (129 mg, 0,50 mmol) and 1,4-Diazabicyclo[2.2. 2]octane (51 mg; 0.46 mmol) at 0°C under a nitrogen atmosphere. The reaction mixture was stirred for 8 h at ambient temperature. The reaction mixture was quenched with water and extracted with EtOAc (1x 5ml and 2x 20 mL) . The combined organic layers were washed with brine dried and concentrated under reduced pressure. Purification of the crude material by FCC (MeOH/DCM 5:95) provided the desired product **8** (60 mg; 47% yield).

$^1$H NMR (500 MHz, CHLOROFORM-d) δ 8.46 (d, *J* = 5.8 Hz, 1H), 6.86 (d, *J* = 6.0 Hz, 1H), 5.33 (quin, *J* = 6.2 Hz, 1H), 3.10 – 3.05 (m, 1H), 2.60 (dt, *J* = 9.1, 5.7 Hz, 1H), 2.45 (s, 3H), 2.30 (td, *J* = 9.3, 7.0 Hz, 1H), 1.94 – 1.85 (m, 1H), 1.81 – 1.65 (m, 3H), 1.33 (d, *J* = 6.3 Hz, 3H).

<u>(S)-2-amino-4-methyl-4-(3-(4-((S)-1-((S)-1-methylpyrrolidin-2-yl)ethoxy)pyrimidin-2-yl)-1,2,4-oxadiazol-5-yl)-4,5,6,7-tetrahydrobenzo[b]thiophene-3-carbonitrile (**BI-2865**):</u>

Nitrile **8** (379 mg, 1.63 mmol) was dissolved in EtOH (3 ml) and treated with 50% aq. hydroxylamine (200 µl, 3.26 mmol). The reaction mixture was heated to 60 °C for two hours. The solvents were removed under reduced pressure and the crude hydroxamidine was subjected to the next reaction. Acid **4** (385 mg, 1.63 mmol) and HATU (743 mg, 1,95 mmol) were dissolved in DMSO (2 ml) and treated with triethylamine (0.56 ml, 4.07 mmol). The mixture was stirred at ambient temperature for 15 min before it was added to the crude hydroxamidine. The reaction

mixture was stirred overnight. After filtration the crude mixture was subjected to RP-HPLC chromatography with resulted in 248 mg (32% yield) of an acylated hydroxamidine which was not further characterized. The material was dissolved in THF (3 ml) and treated with DBU (0.15 ml, 1.03 mmol) and heated to 60 °C overnight. The solvent was evaporated under reduced pressure and the crude product was subjected to RP-HPLC which resulted in 158 mg of **BI-2865** (66 %yield).

$^{1}$H NMR (600 MHz, DMSO-$d_6$) δ 8.68 (d, *J* = 5.7 Hz, 1H), 7.08 (br s, 2H), 7.08 – 7.05 (m, 1H), 5.37 (quin, *J* = 6.0 Hz, 1H), 2.95 (br t, *J* = 7.5 Hz, 1H), 2.70 – 2.62 (m, 1H), 2.55 (br t, *J* = 5.9 Hz, 2H), 2.39 (s, 3H), 2.24 – 2.15 (m, 1H), 2.12 – 2.05 (m, 1H), 1.98 – 1.92 (m, 1H), 1.80 (s, 5H), 1.79 – 1.69 (m, 2H), 1.69 – 1.58 (m, 2H), 1.28 (d, *J* = 6.4 Hz, 3H)

$^{13}$C NMR (151 MHz, DMSO-$d_6$) δ 185.08, 169.18, 167.67, 164.18, 159.04, 155.32, 131.91, 120.15, 116.29, 110.27, 81.86, 74.96, 66.68, 57.62, 42.31, 39.23, 37.47, 26.11, 25.02, 24.08, 24.02, 19.92, 15.01

HRMS (ESI) m/z: $[M + H]^+$ Calcd for C23H27N7O2S 466.20197; Found 466.20271.

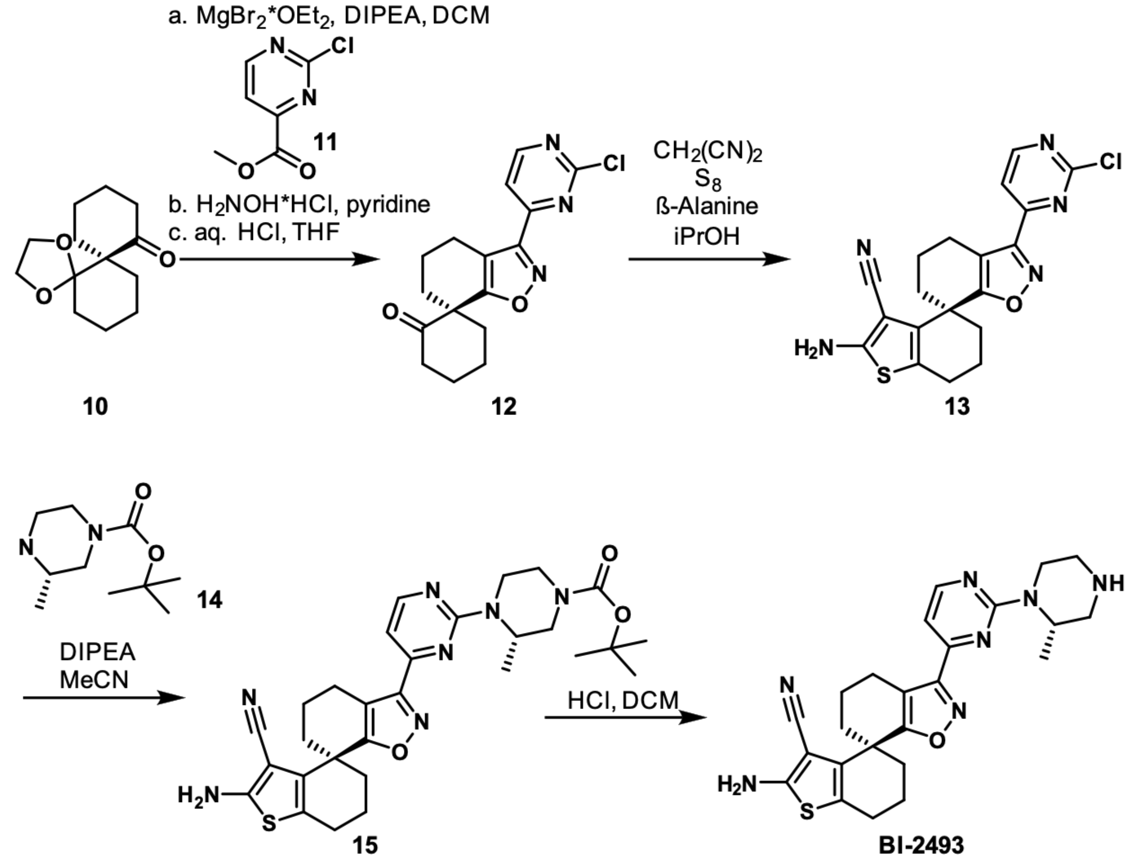


(7S)-3-(2-chloropyrimidin-4-yl)-5,6-dihydro-4H-spiro[1,2-benzoxazole-7,1'-cyclohexan]-6'-one (**12**):

Enantiopure spiro ketone **10** was obtained from the previously described racemate[55] via chiral separation (SFC using a Lux Cellulose-4 column (250x30mm, 5µm), 30°C column temperature, 90% $CO_2$, 10% MeCN as cosolvent) with enantiomer **10** eluting as the second peak after the other enantiomer. Spiro ketone **10** (6.62 g, 28.1 mmol) and magnesium bromide diethyl etherate (8.02 g, 30.7 mmol) were dissolved in dry DCM (50 mL) and stirred for 5 min at rt. DIPEA (7.5 mL, 43.0 mmol) was added, after 5 min followed by ester **11** (5.08 g, 28.3 mmol). The reaction was stirred for 16 h at rt. After complete conversion of the starting material, 1 M HCl was added, and the mixture was stirred for 30 min. The mixture was extracted with DCM/ water, and the organic phase was concentrated under reduced pressure and purified by FCC chromatography (EtOAc/c-hexane 0:100-10:90), yielding 3.96 g (39% yield) of (6S)-8-(2-chloropyrimidine-4-carbonyl)-1,4-

dioxadispiro[4.0.56.45]pentadecan-7-one as a mixture of 3 tautomers which were not further characterized. In the next step (6S)-8-(2-chloropyrimidine-4-carbonyl)-1,4-dioxadispiro[4.0.56.45]pentadecan-7-one (1.02 g, 2.78 mmol) was dissolved in pyridine (86 mL) and hydroxylamine hydrochloride (238 mg, 3.43 mmol) was added. The reaction was stirred overnight at rt. After complete conversion the mixture was acidified with 1 M HCl and extracted with DCM. The combined organic phases were concentrated under reduced pressure and purified by basic RP-HPLC, yielding 335 mg (32% yield) of (6S)-8-[(Z)-(2-chloropyrimidin-4-yl)(hydroxyimino)methyl]-1,4-dioxadispiro[4.0.56.45] -pentadecan-7-one as a mixture of 3 tautomers which were not further characterized. In the next step (6S)-8-[(Z)-(2-chloropyrimidin-4-yl)(hydroxyimino)methyl]-1,4-dioxadispiro[4.0.56.45] -pentadecan-7-one (335 mg, 0.88 mmol) was dissolved in THF (4.0 mL) and aq. HCl (4 M, 4.0 ml, 16.0 mmol) and stirred for 2 d at rt. After complete conversion, the mixture was concentrated under reduced pressure. The precipitate was collected by filtration and dried under reduced pressure yielding 231mg (83% yield) of isoxazole **12.**

$^{1}$H NMR (600 MHz, DMSO-$d_6$) δ 8.93 (d, J = 5.4 Hz, 1H), 8.05 (d, J = 5.0 Hz, 1H), 2.84 – 2.74 (m, 2H), 2.66 (ddd, J = 16.6, 9.0, 5.7 Hz, 1H), 2.47 – 2.41 (m, 1H), 2.36 – 2.30 (m, 1H), 2.30 – 2.23 (m, 1H), 2.07 – 2.01 (m, 1H), 2.01 – 1.94 (m, 1H), 1.93 – 1.74 (m, 5H), 1.55 – 1.44 (m, 1H)

(4S)-2-amino-3'-(2-chloropyrimidin-4-yl)-5',6,6',7-tetrahydro-4'H,5H-spiro[1-benzothiophene-4,7'-[1,2]benzoxazole]-3-carbonitrile (**13**):

To a solution of **12** (1.00 g, 2.90 mmol) and mol. sieves (3Å) in anhydrous isopropanol (16 mL) under an argon atmosphere were added malononitrile (383 mg, 5.52 mmol), sulfur (179 mg, 5.51 mmol) and ß-Alanine (500 mg, 5.61 mmol). The reaction mixture was stirred at 90°C overnight. After complete conversion, the reaction mixture was cooled to rt, filtered, and extracted with DCM and water. The organic phases were combined and concentrated under reduced pressure. The residue was dissolved in in MeCN and water and purified by basic RP-HPLC yielding 620mg (54% yield) of amino-cyano-thiophene **13**.

$^{1}$H NMR (600 MHz, DMSO-$d_6$) δ 8.92 (d, J = 5.0 Hz, 1H), 8.05 (d, J = 5.0 Hz, 1H), 6.98 (s, 2H), 3.01 (br dd, J = 16.6, 4.6 Hz, 1H), 2.67 – 2.53 (range, 3H), 2.12 – 2.05 (m, 1H), 2.04 – 1.91 (range, 3H), 1.90 – 1.74 (range, 4H)

tert-butyl (3S)-4-{4-[(4S)-2-amino-3-cyano-5',6,6',7-tetrahydro-4'H,5H-spiro[1-benzothiophene-4,7'-[1,2]benzoxazol]-3'-yl]pyrimidin-2-yl}-3-methylpiperazine-1-carboxylate (**15**):

Amino-cyano-thiophene **13** (150 mg, 0.377 mmol), (3S)-3-methyl-1-piperazinecarboxylic acid tert-butyl ester (**14**, 238 mg, 1.13 mmol), and dry DIPEA (197 µL, 1.13 mmol) were dissolved in dry MeCN (3 mL) and stirred for 12 h at 90°C. After complete conversion the mixture was filtered and concentrated under reduced pressure. The crude product was purified by basic RP-HPLC yielding 140mg (66% yield) of **15**.

$^{1}$H NMR (600 MHz, DMSO-$d_6$) δ 8.51 (d, J = 5.1 Hz, 1H), 7.16 (d, J = 5.1 Hz, 1H), 6.92 (s, 2H), 4.80 (br d, J = 2.0 Hz, 1H), 4.39 (br d, J = 13.7 Hz, 1H), 4.06 – 3.95 (m, 1H), 3.84 (br s, 2H), 3.16 (br d, J = 9.4 Hz, 1H), 3.07 (br dd, J = 16.6, 3.7 Hz, 1H), 2.63 (ddd, J = 16.4, 11.3, 5.4 Hz, 1H), 2.12 – 1.70 (m, 9H), 1.44 (s, 9H), 1.12 (d, J = 6.6 Hz, 3H)

(4S)-2-amino-3'-{2-[(2S)-2-methylpiperazin-1-yl]pyrimidin-4-yl}-5',6,6',7-tetrahydro-4'H,5H-spiro[1-benzothiophene-4,7'-[1,2]benzoxazole]-3-carbonitrile (**BI-2493**)

**15** (140 mg, 0.25 mmol) was dissolved in DCM (2.0 mL), HCl in dioxane (4M, 0.75 mL, 2.99 mmol) was added and the reaction was stirred for 2 h at rt. After complete conversion, the reaction was concentrated under reduced pressure, diluted with small amounts of DMF and water, basified with DIPEA and purified by RP-HPLC yielding 46mg (40%) of **BI-2493**.

$^{1}$H NMR (600 MHz, DMSO-$d_6$) δ 8.46 (d, J = 5.0 Hz, 1H), 7.08 (d, J = 5.0 Hz, 1H), 6.94 (s, 2H), 4.75 – 4.62 (m, 1H), 4.37 – 4.28 (m, 1H), 3.10 – 2.92 (m, 3H), 2.84 – 2.73 (m, 2H), 2.65 – 2.52 (m, 4H), 2.09 – 1.89 (m, 4H), 1.88 – 1.72 (m, 4H), 1.20 (d, J = 6.6 Hz, 3H)

$^{13}$C NMR (151 MHz, DMSO-$d_6$) δ 172.39, 163.56, 161.15, 159.15, 158.11, 156.91, 132.84, 119.68, 115.11, 113.98, 106.14, 81.48, 50.08, 46.26, 45.63, 38.21, 33.40, 32.67, 23.77, 21.11, 19.58, 19.32, 13.80

## Supplementary References


41 Ostrem, J. M. & Shokat, K. M. Direct small-molecule inhibitors of KRAS: from structural insights to mechanism-based design. *Nat Rev Drug Discov* **15**, 771-785, doi:10.1038/nrd.2016.139 (2016).

42 Westover, K. D., Janne, P. A. & Gray, N. S. Progress on Covalent Inhibition of KRAS(G12C). *Cancer Discov* **6**, 233-234, doi:10.1158/2159-8290.CD-16-0092 (2016).

43 Papke, B. & Der, C. J. Drugging RAS: Know the enemy. *Science* **355**, 1158-1163, doi:10.1126/science.aam7622 (2017).

44 Xiong, Y. *et al.* Covalent Guanosine Mimetic Inhibitors of G12C KRAS. *ACS Med Chem Lett* **8**, 61-66, doi:10.1021/acsmedchemlett.6b00373 (2017).

45 Teng, K. W. *et al.* Selective and noncovalent targeting of RAS mutants for inhibition and degradation. *Nat Commun* **12**, 2656, doi:10.1038/s41467-021-22969-5 (2021).

46 Vasta, J. D. *et al.* KRAS is vulnerable to reversible switch-II pocket engagement in cells. *Nat Chem Biol* **18**, 596-604, doi:10.1038/s41589-022-00985-w (2022).

47 Zhang, Z., Guiley, K. Z. & Shokat, K. M. Chemical acylation of an acquired serine suppresses oncogenic signaling of K-Ras(G12S). *Nat Chem Biol*, doi:10.1038/s41589-022-01065-9 (2022).

48 Zhang, Z., Morstein, J., Ecker, A. K., Guiley, K. Z. & Shokat, K. M. Chemoselective Covalent Modification of K-Ras(G12R) with a Small Molecule Electrophile. *J Am Chem Soc*, doi:10.1021/jacs.2c05377 (2022).

49 Rabara, D. *et al.* KRAS G13D sensitivity to neurofibromin-mediated GTP hydrolysis. *Proc Natl Acad Sci U S A* **116**, 22122-22131, doi:10.1073/pnas.1908353116 (2019).

50 Nichols, R. J. *et al.* RAS nucleotide cycling underlies the SHP2 phosphatase dependence of mutant BRAF-, NF1- and RAS-driven cancers. *Nat Cell Biol* **20**, 1064-1073, doi:10.1038/s41556-018-0169-1 (2018).

51 Hofmann, M. H. *et al.* Abstract PL06-01: Discovery of BI-3406: A potent and selective SOS1::KRAS inhibitor opens a new approach for treating KRAS-driven tumors. *Molecular Cancer Therapeutics* **18**, PL06-01-PL06-01, doi:10.1158/1535-7163.Targ-19-pl06-01 (2019).

52 Munoz-Maldonado, C., Zimmer, Y. & Medova, M. A Comparative Analysis of Individual RAS Mutations in Cancer Biology. *Front Oncol* **9**, 1088, doi:10.3389/fonc.2019.01088 (2019).

53 Hobbs, G. A. *et al.* Atypical KRAS(G12R) Mutant Is Impaired in PI3K Signaling and Macropinocytosis in Pancreatic Cancer. *Cancer Discov* **10**, 104-123, doi:10.1158/2159-8290.CD-19-1006 (2020).

54 Poulin, E. J. *et al.* Tissue-Specific Oncogenic Activity of KRAS(A146T). *Cancer Discov* **9**, 738-755, doi:10.1158/2159-8290.CD-18-1220 (2019).

55 Markó, I. E. *et al*. Efficient and convergent stereocontrolled spiroannulation of ketones. Tetrahedron Lett 44, 3333-3336, doi:10.1016/S0040-4039(03)00573-2 (2003).

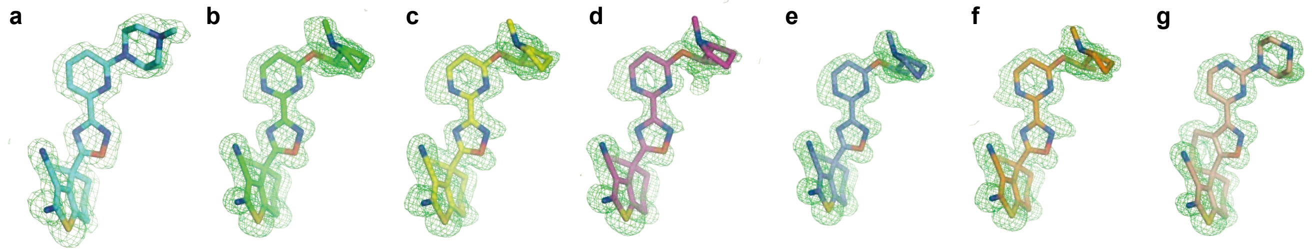

**Supplementary Figure 1.** $F_o - F_c$ electron density maps of the respective crystal structures at a level of 3.0 sigma. Omit maps obtained by a simulated annealing protocol. **a,** precursor 1 in complex with KRAS WT. **b**-**f**, BI-2865 in complex with KRAS WT (b), G12C (c), G12D (d), G12V (e) or G13D (f). **g**, BI-2493 in complex with KRAS WT.

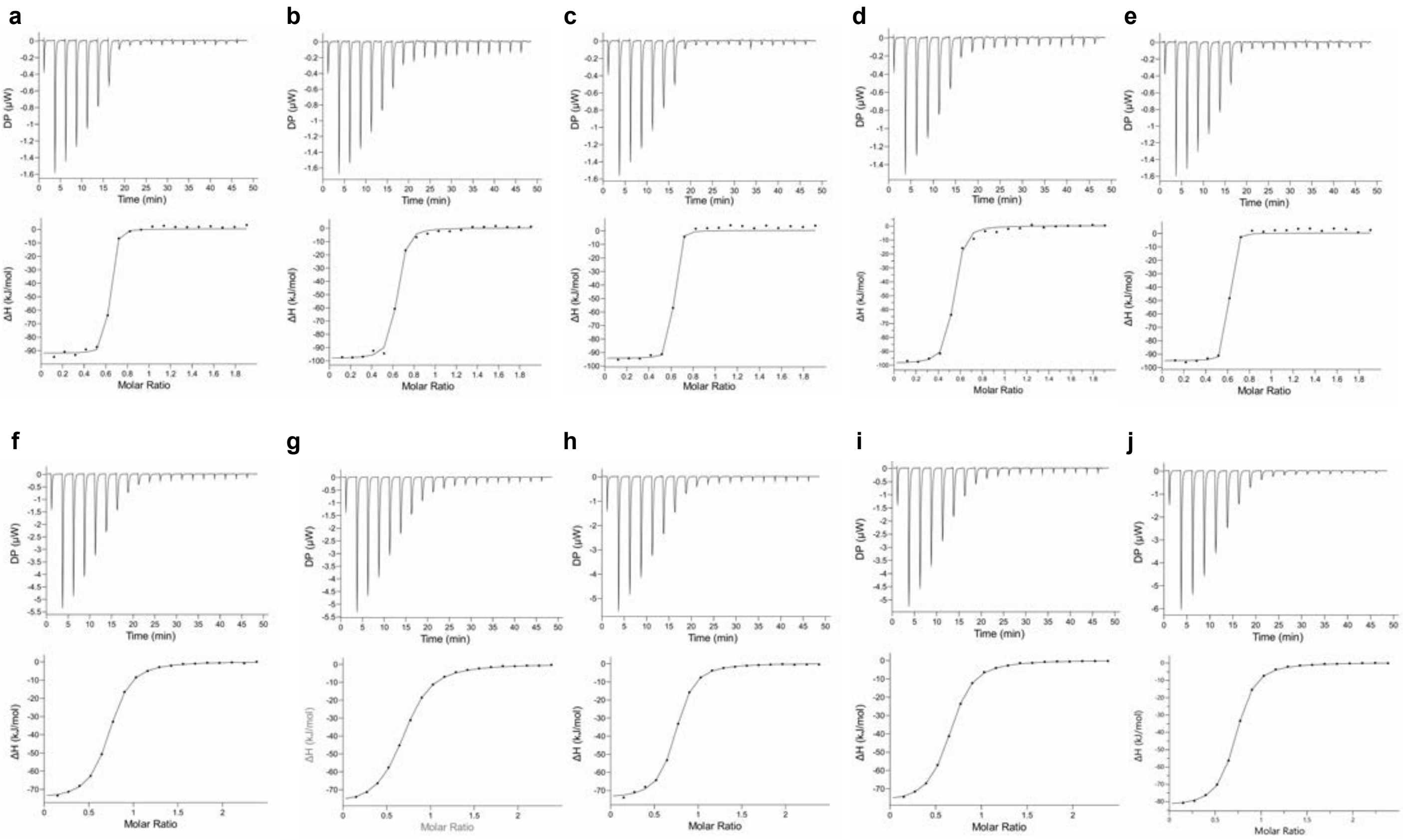


**Supplementary Figure 2. a-e**, KRAS WT (a), G12C (b), G12D (c), G12V (d) and G13D (e) were loaded with GDP and subjected to ITC analysis to determine the interaction with the KRASi. **f-j**, As in A-E but the KRAS variants were loaded with the GTP analogue GCP.

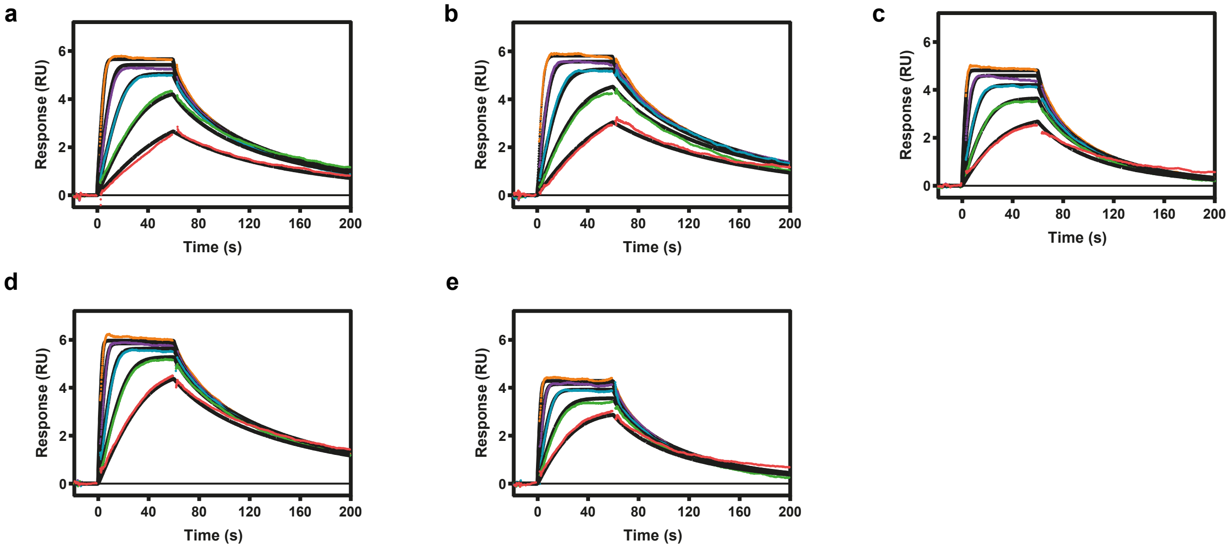


**Supplementary Figure 3. A-E**, SPR sensor-grams and fits for the interaction between BI-2865 and KRAS WT (A), G12C (B), G12D (C), G12V (D) and G13D (E) loaded with the GDP.

**Figure 2a**

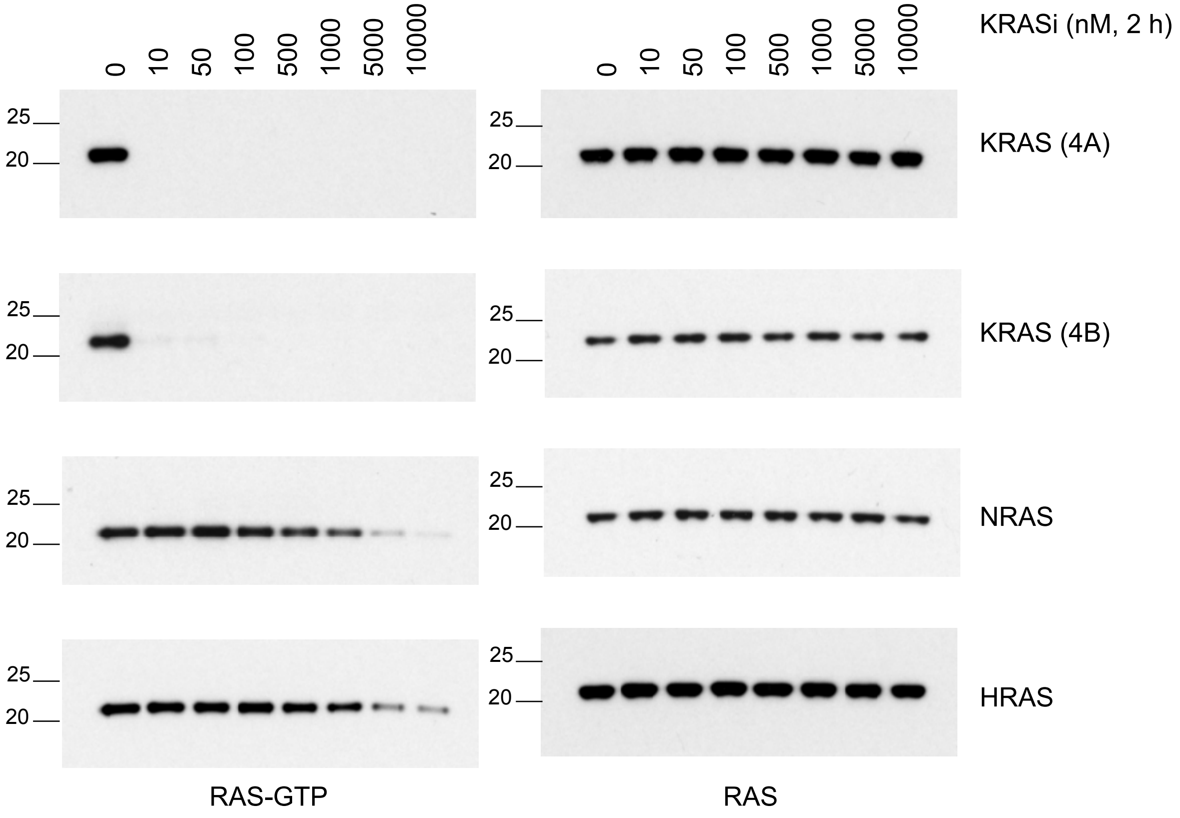


**Extended Data Figure 2i**

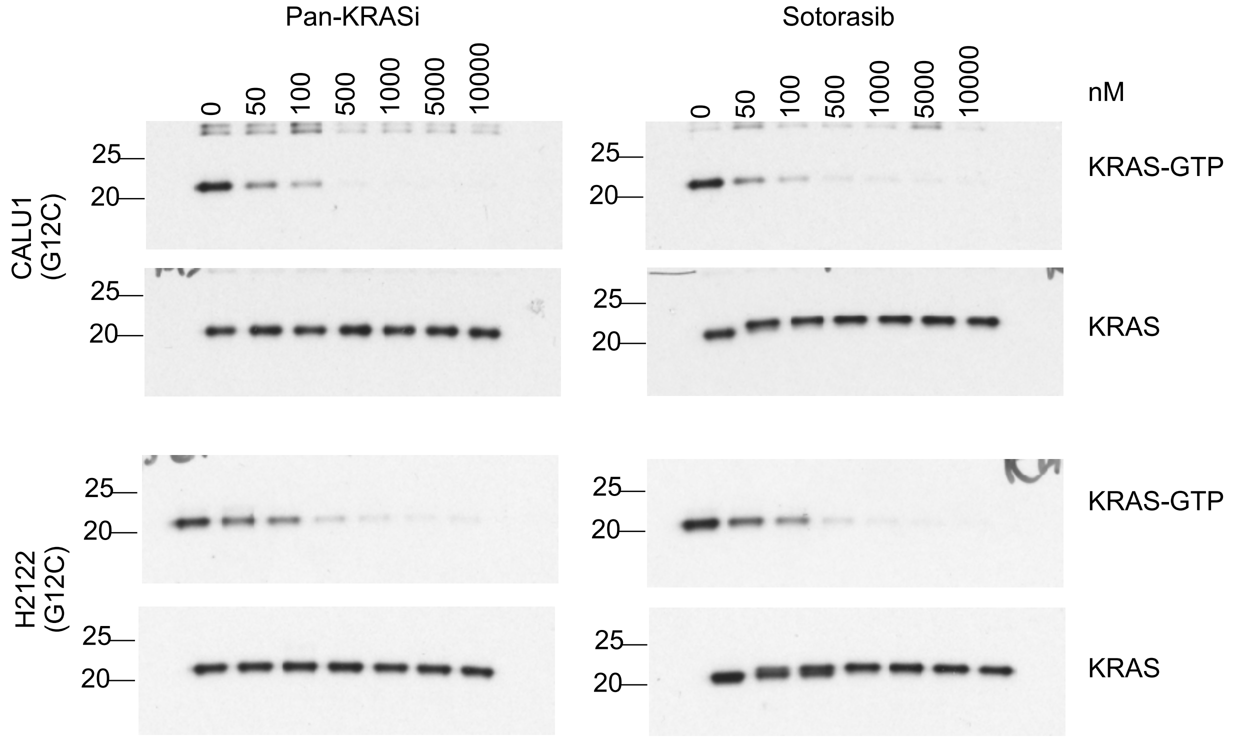


**Supplementary Figure 4.** Images of western blots. Please note loading controls were not always run on the same gel. Membranes were cropped prior to primary antibody exposure.

**Extended Data Figure 2j**

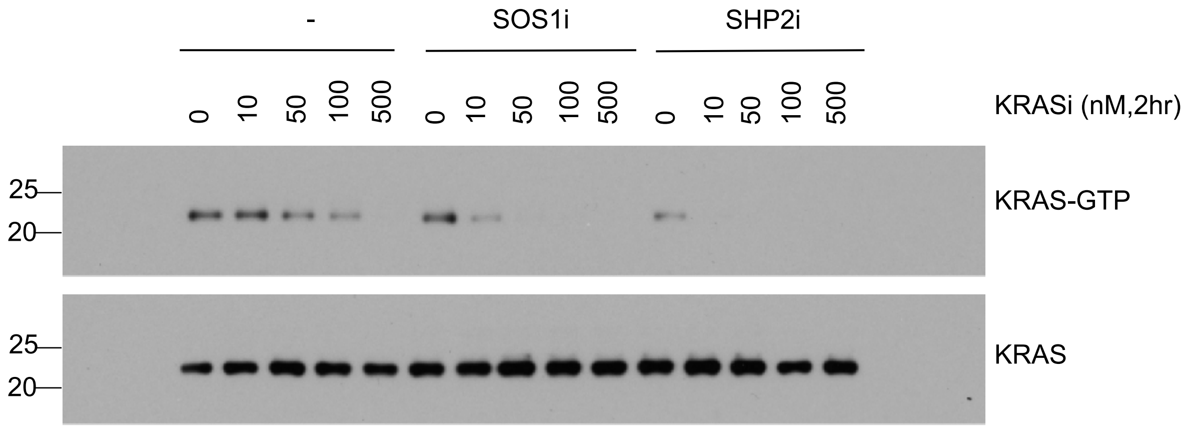


**Extended Data Figure 3a**

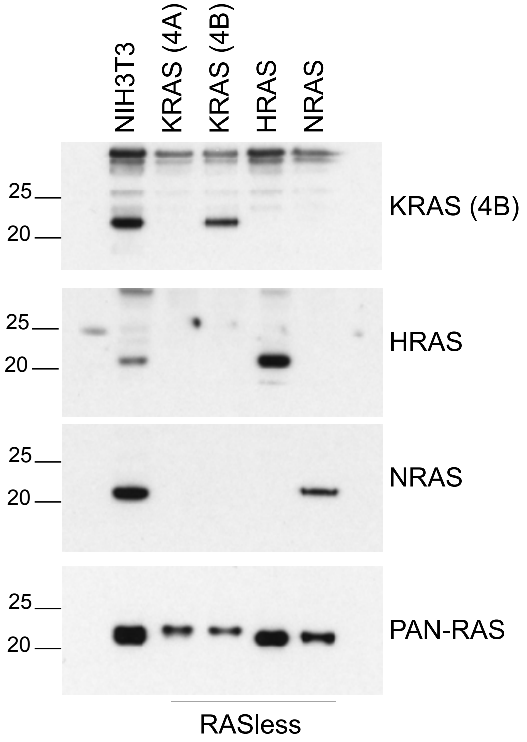

**Extended Data Figure 3b**

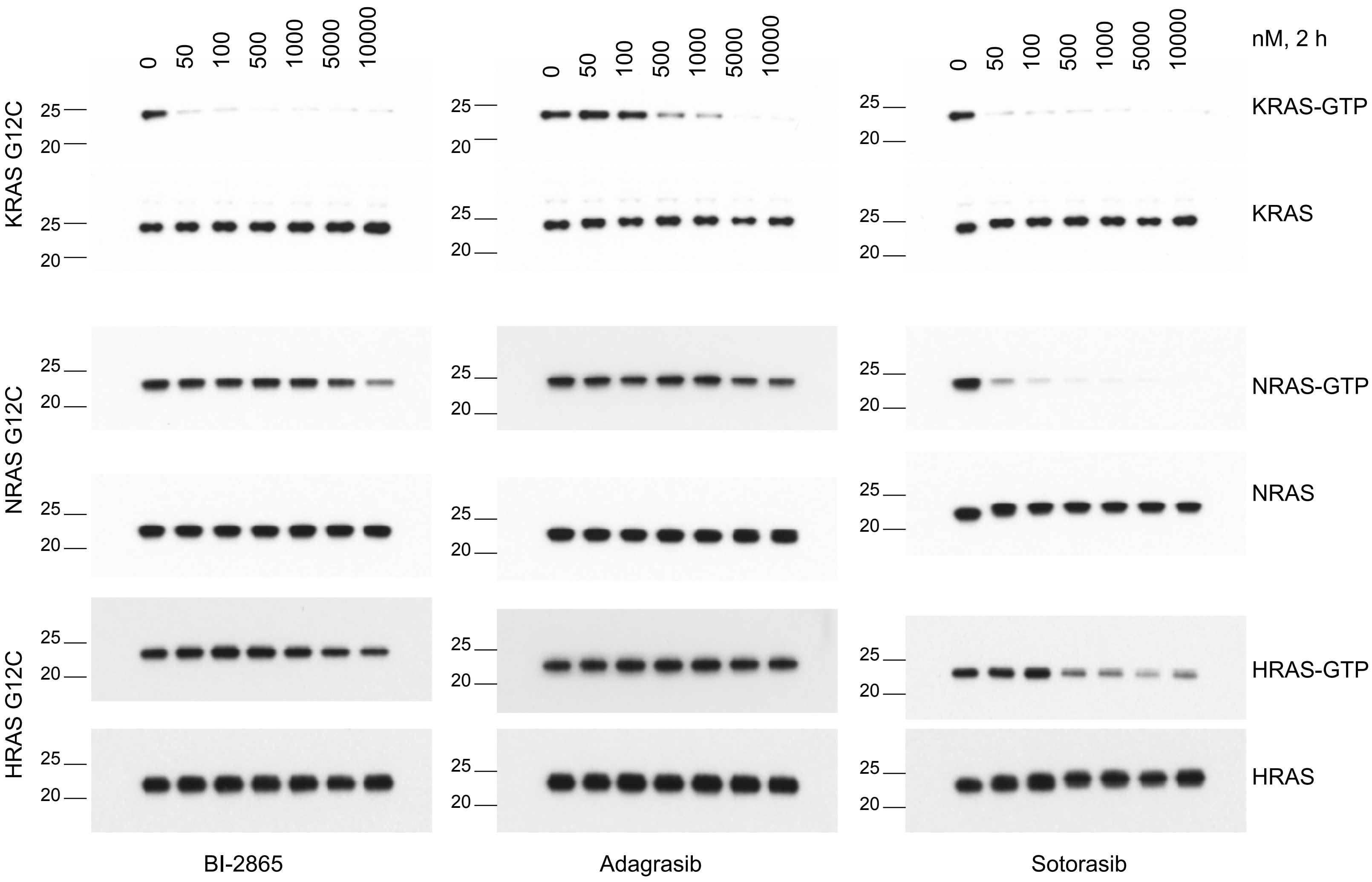

**Extended Data Figure 3c**

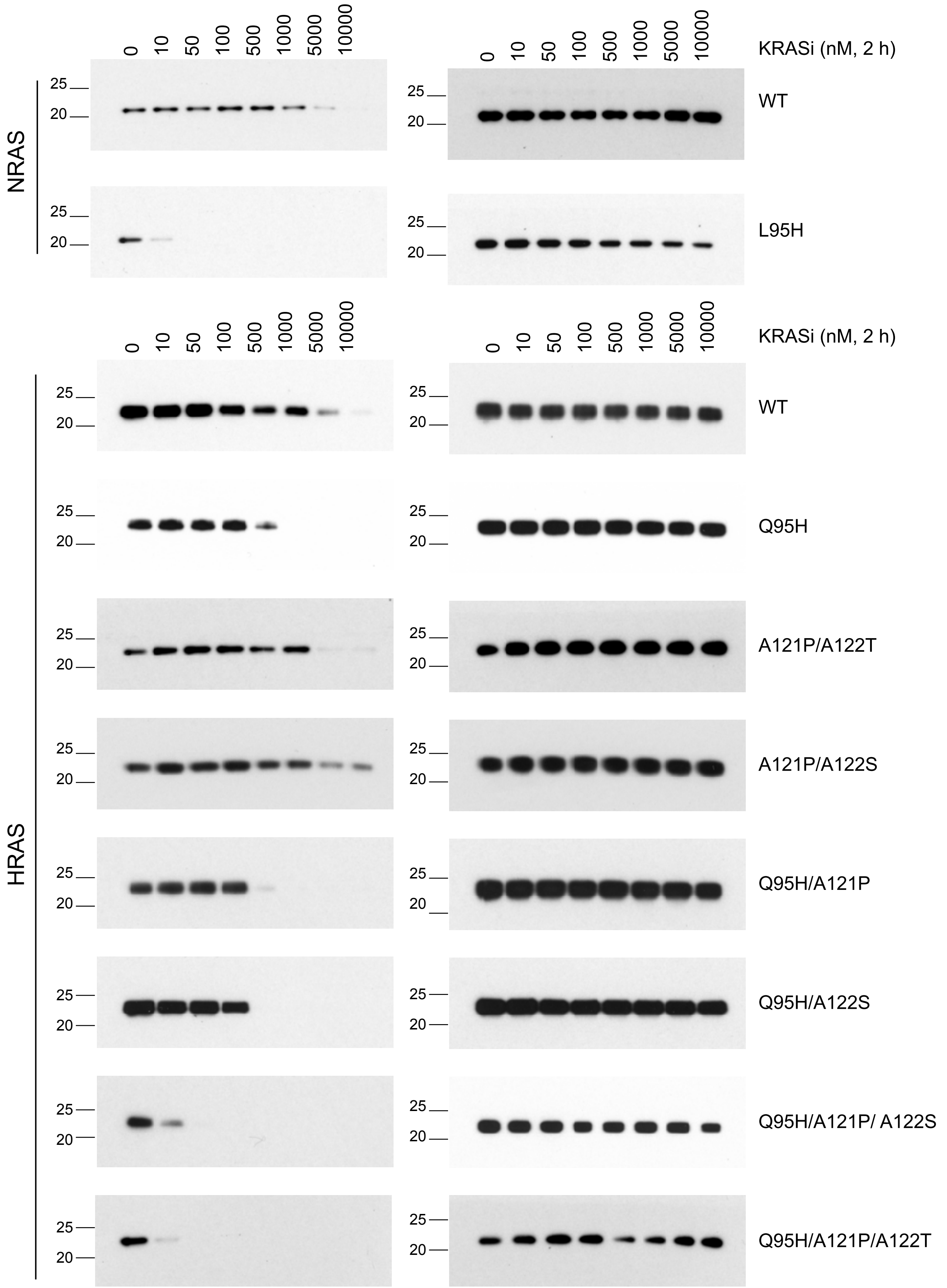

**Extended Data Figure 3c**

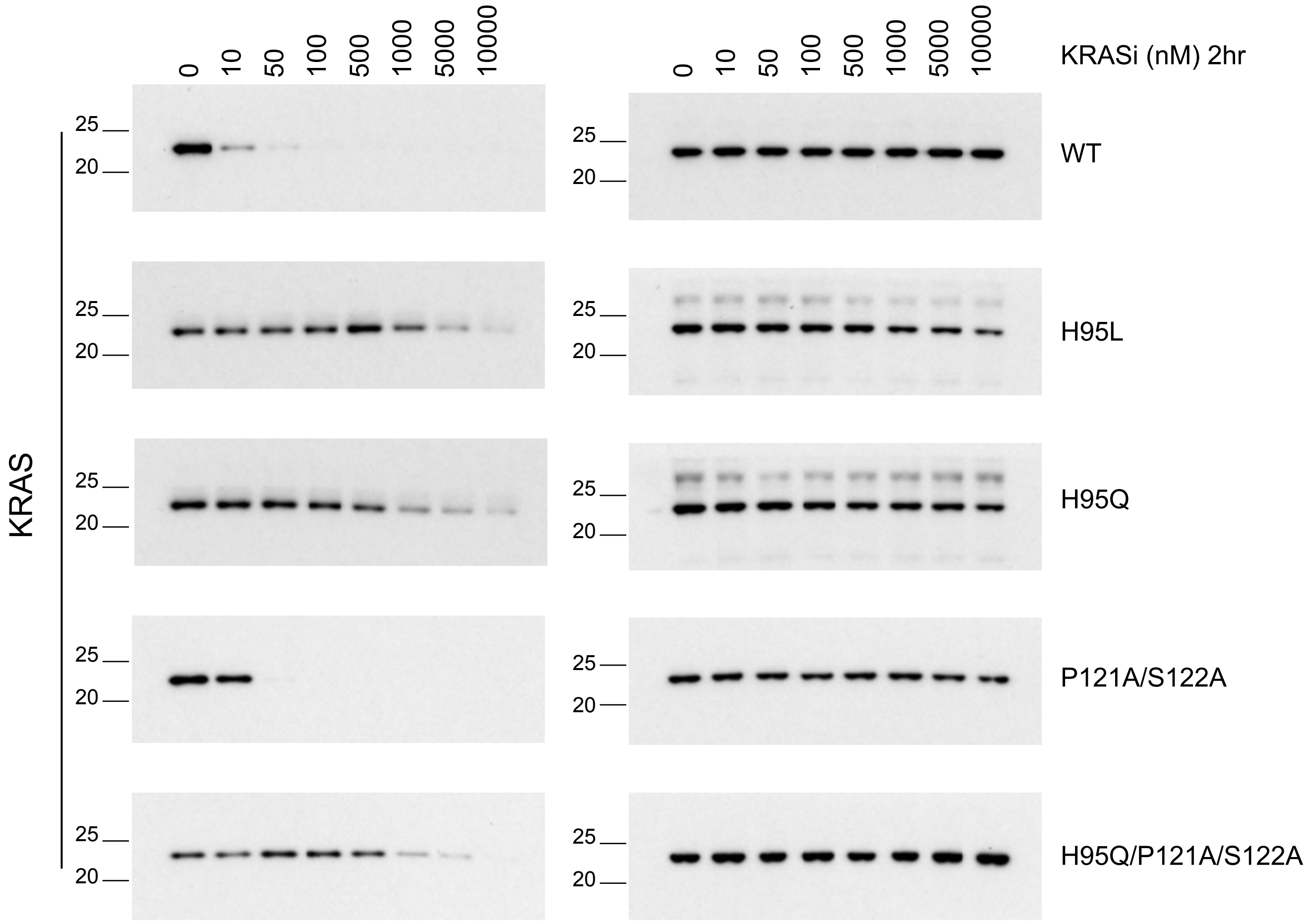

**Extended Data Figure 3d**

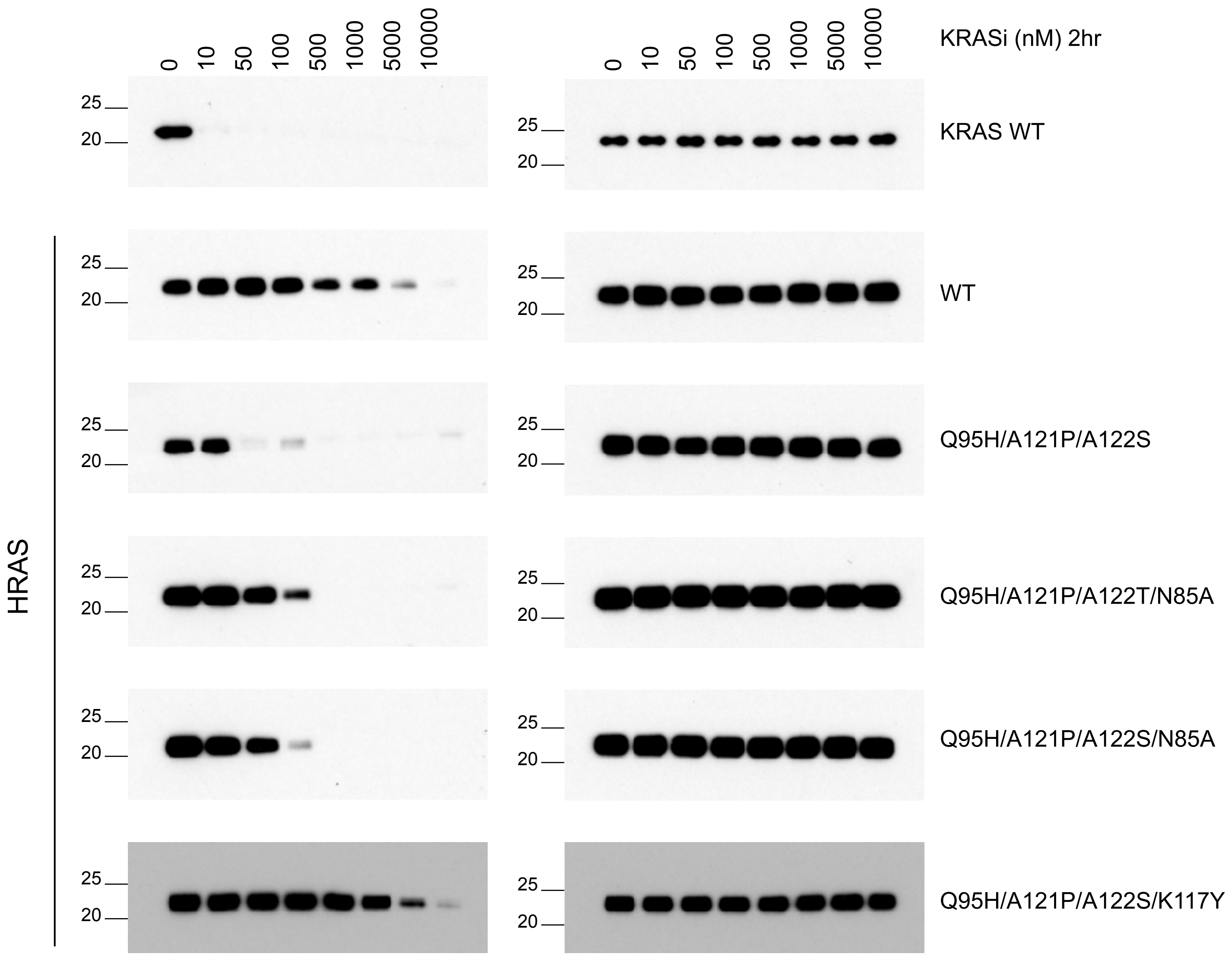

**Extended Data Figure 4e**

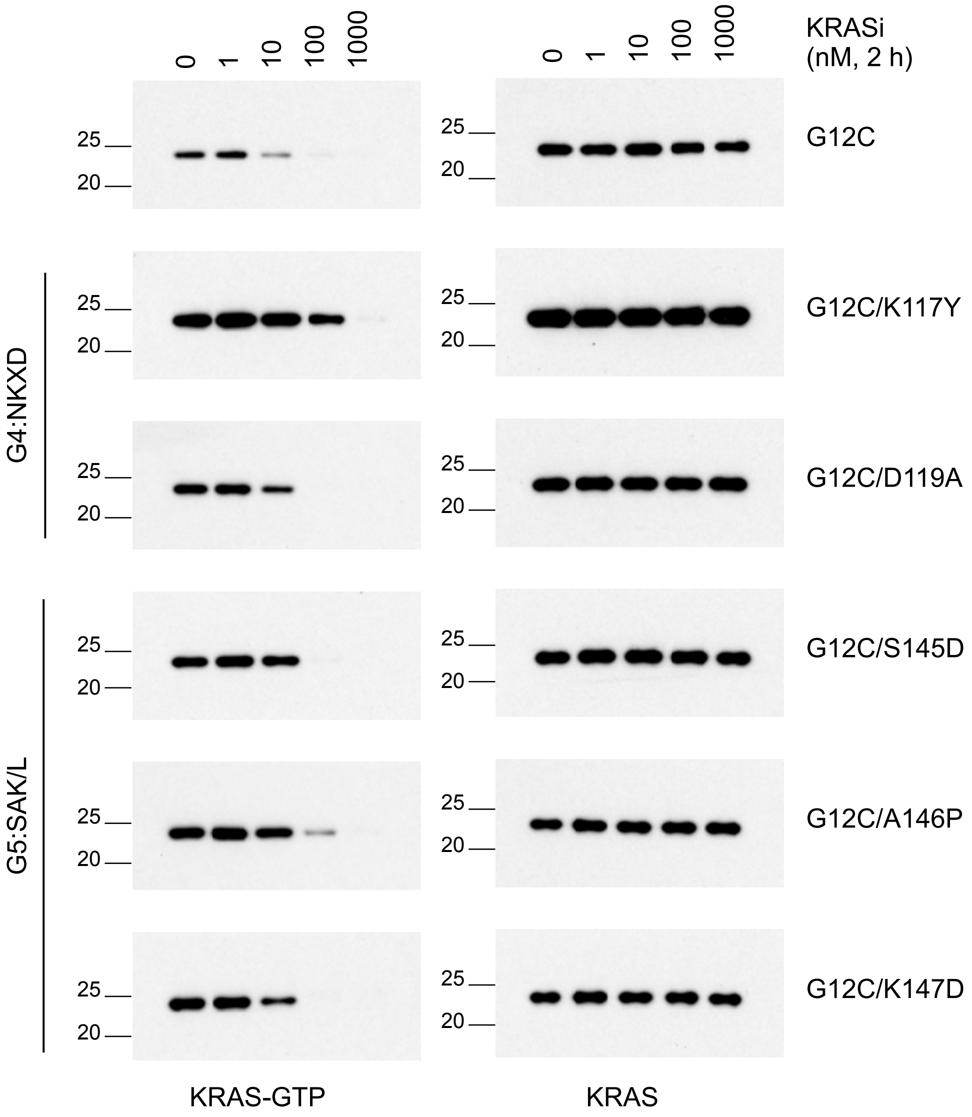

**Extended Data Figure 4f**

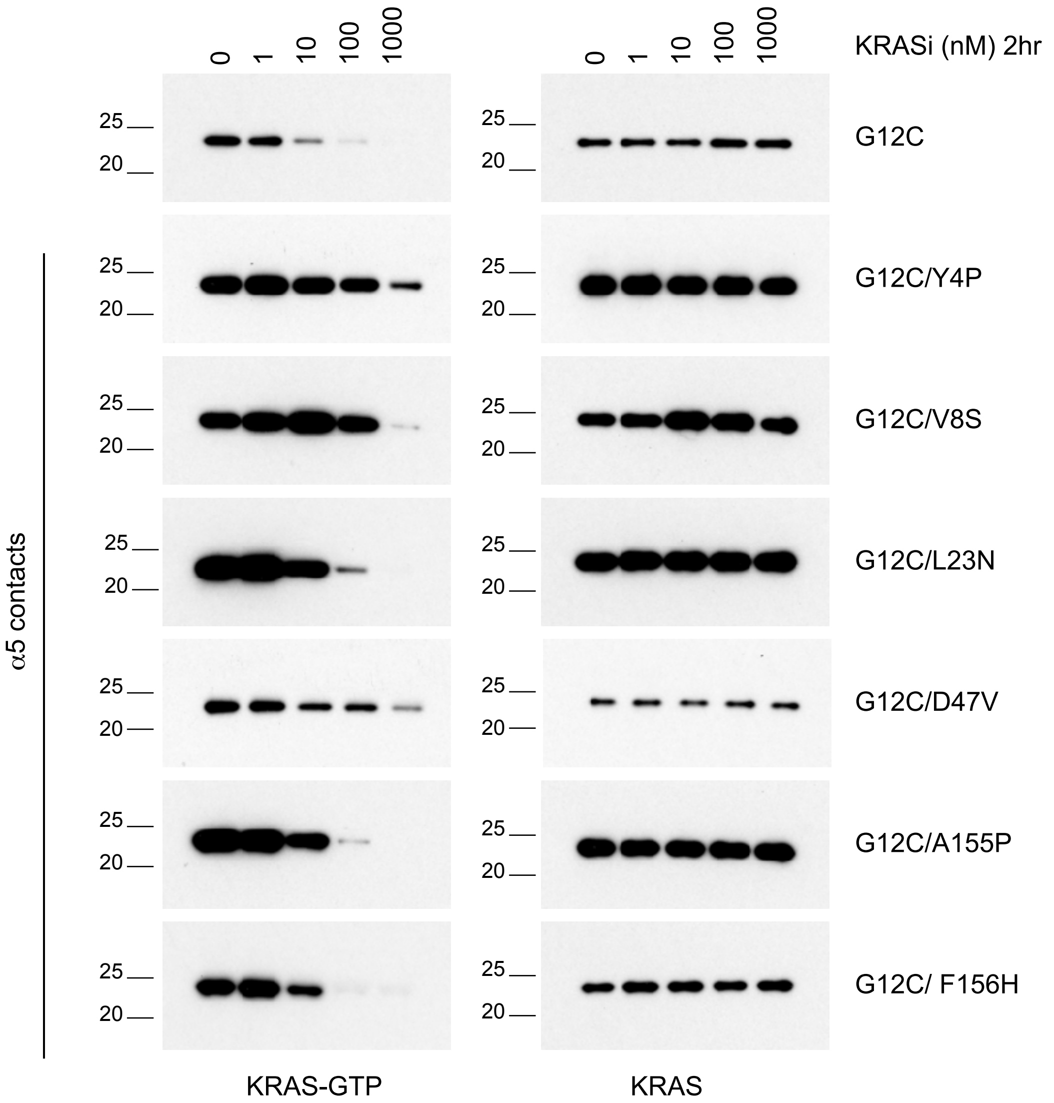

**Extended Data Figure 5**

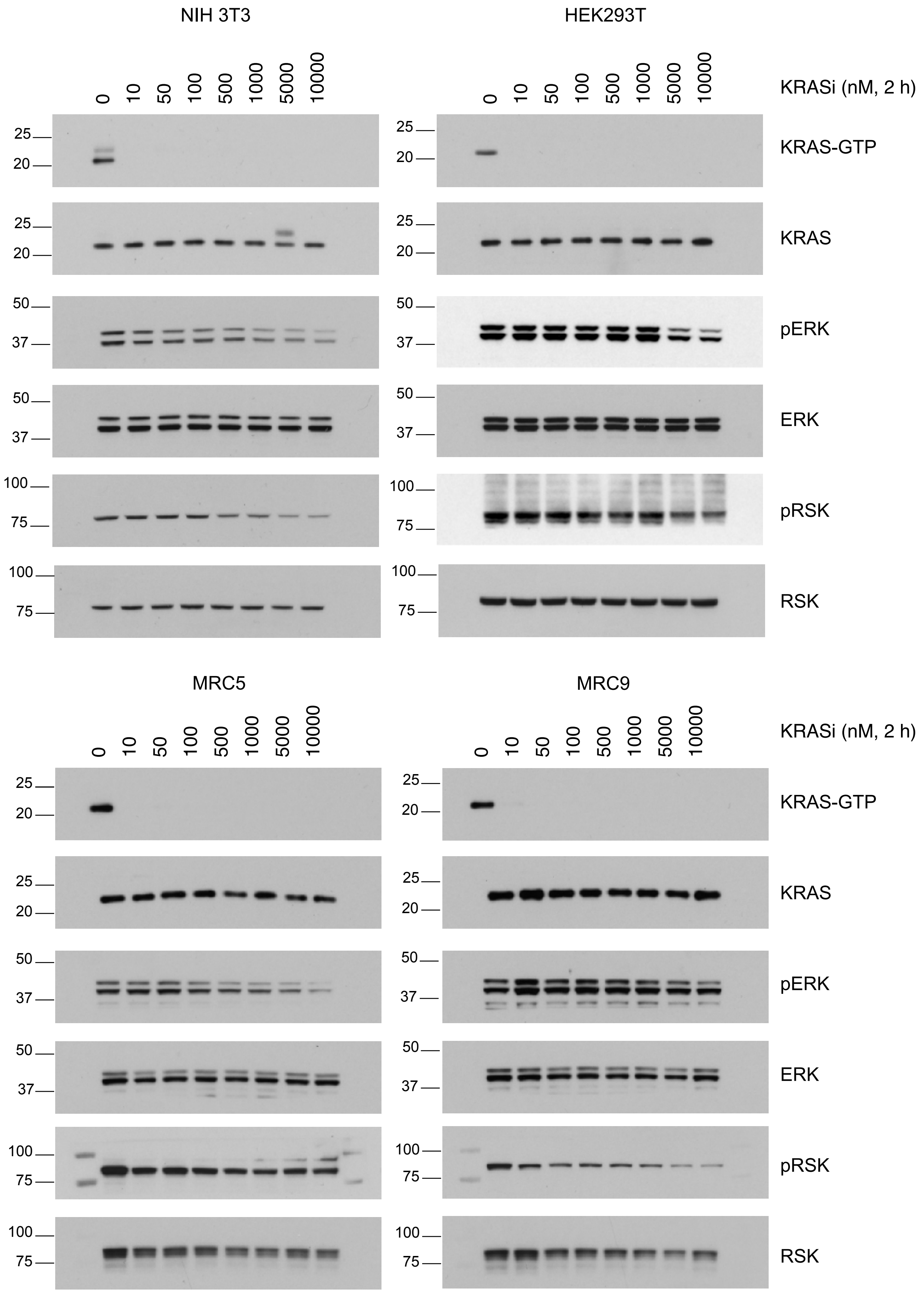

**Extended Data Figure 5**

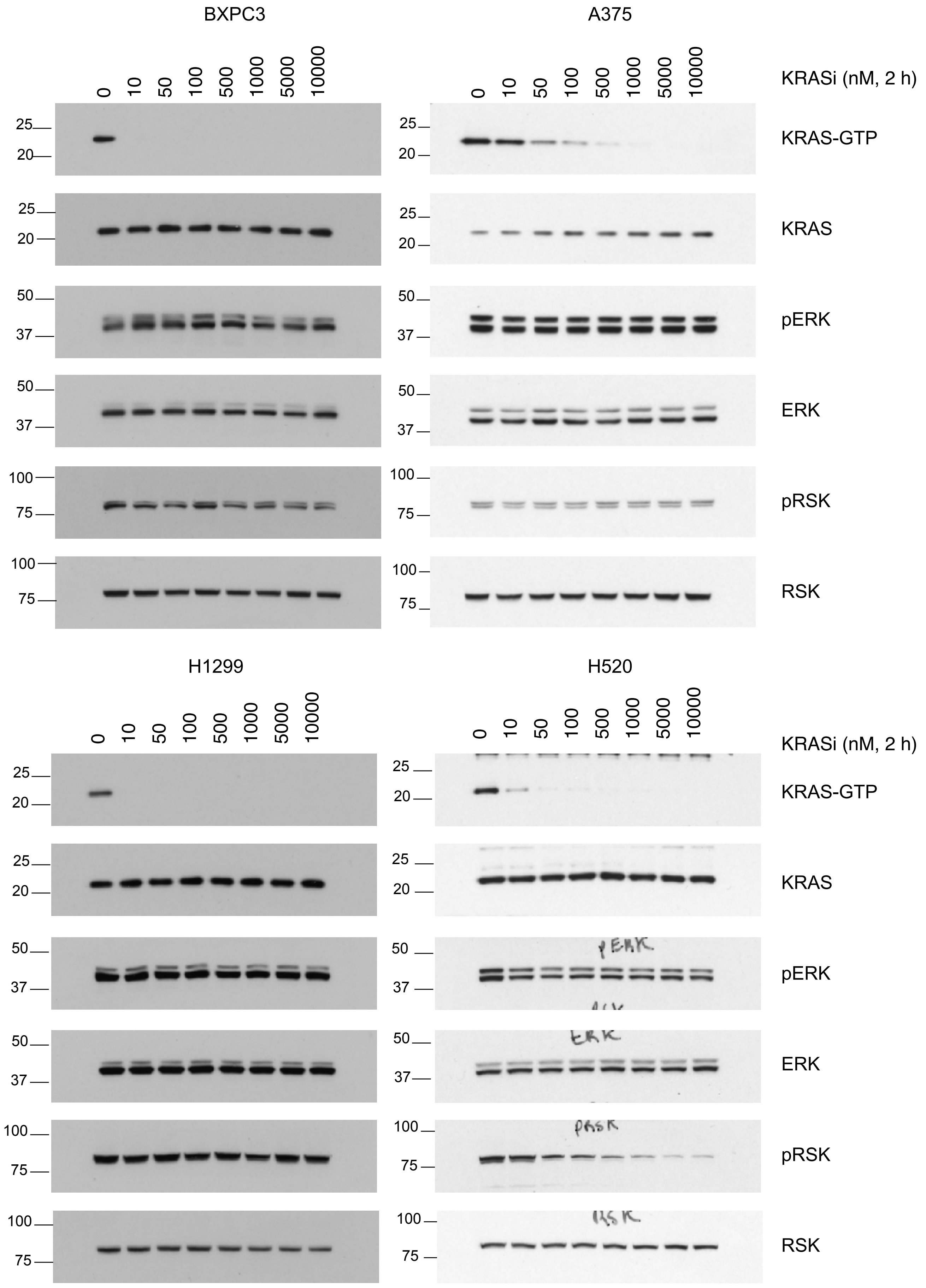

**Extended Data Figure 5**

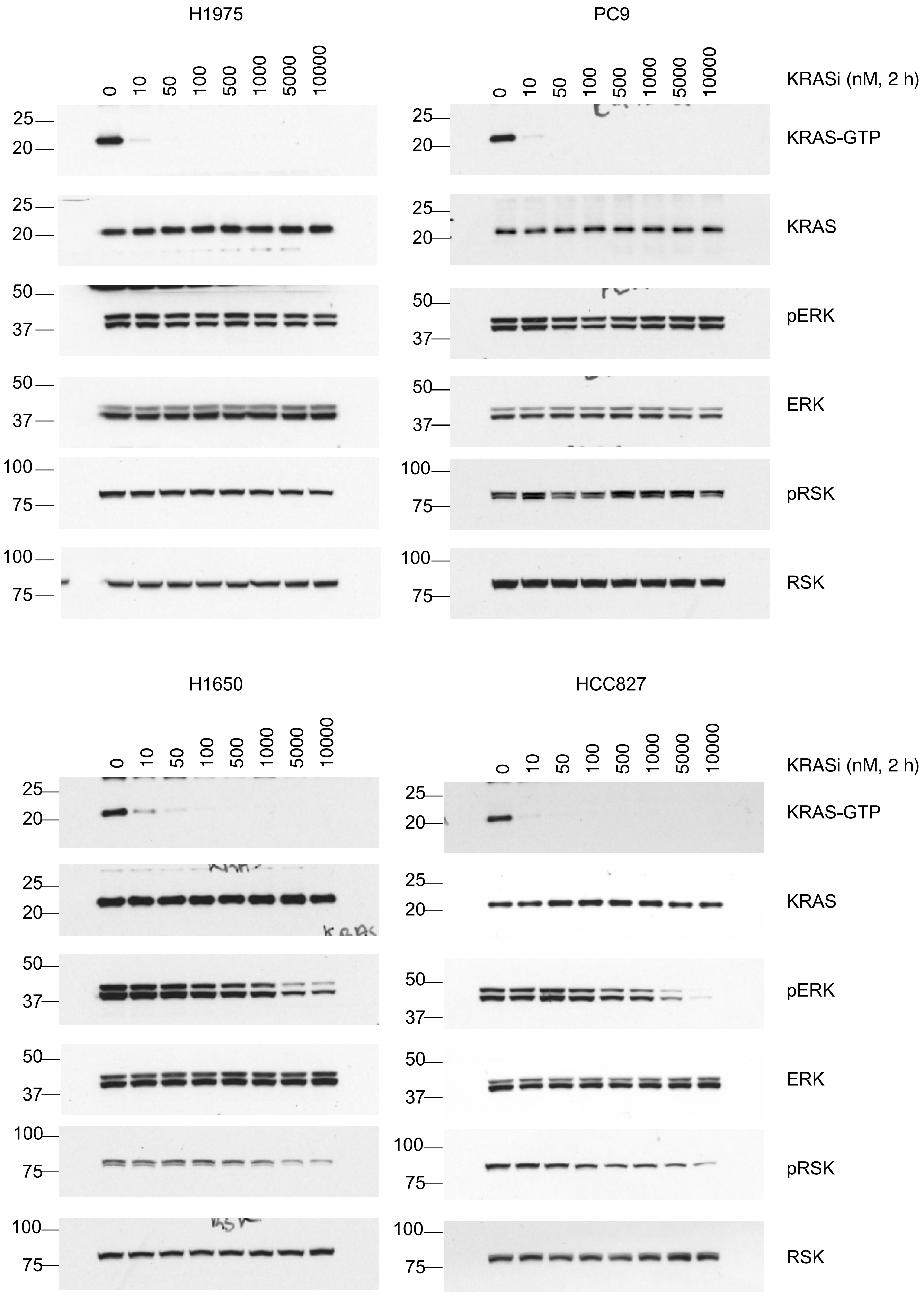

**Extended Data Figure 5**

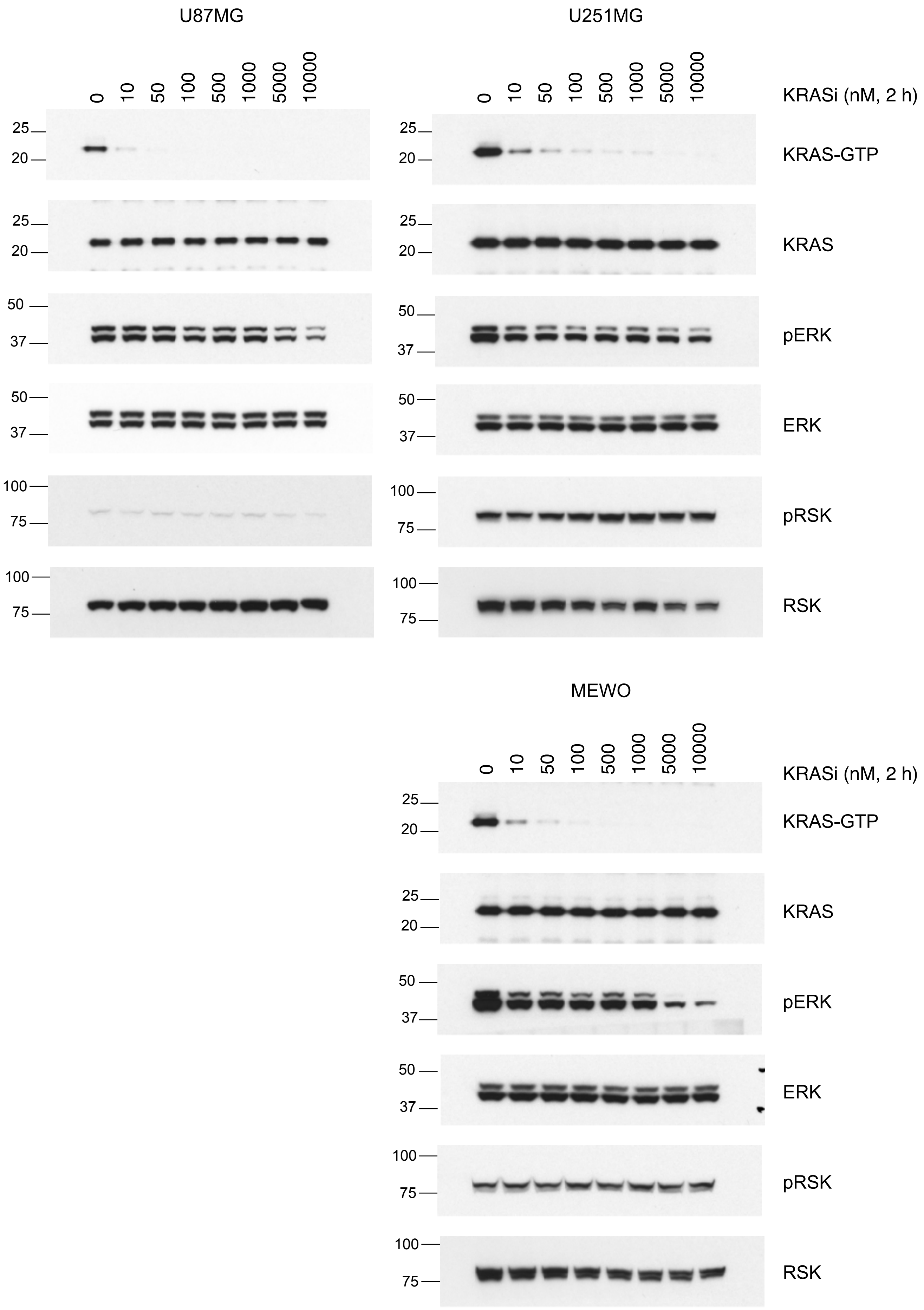

**Extended Data Figure 5**

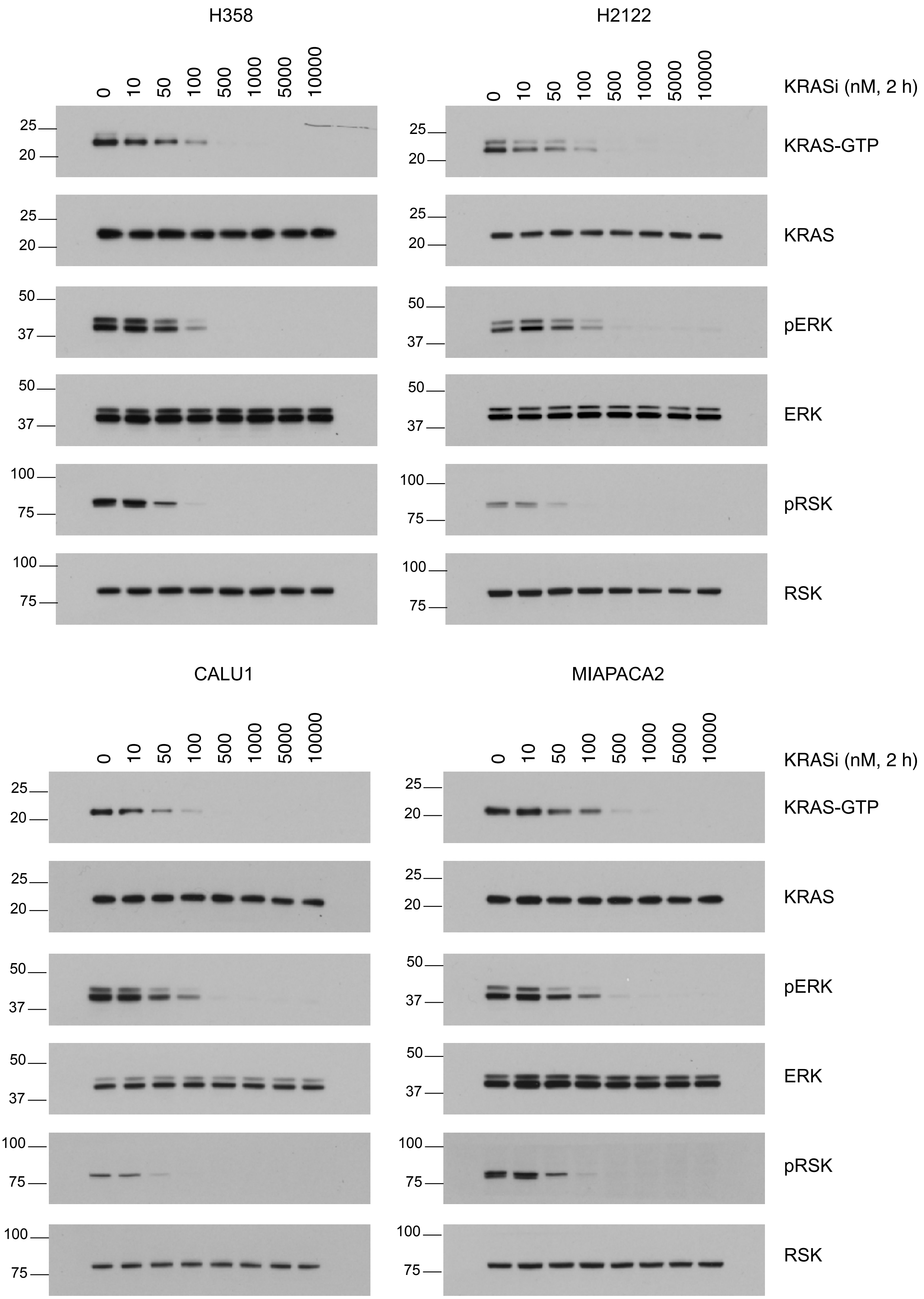

**Extended Data Figure 5**

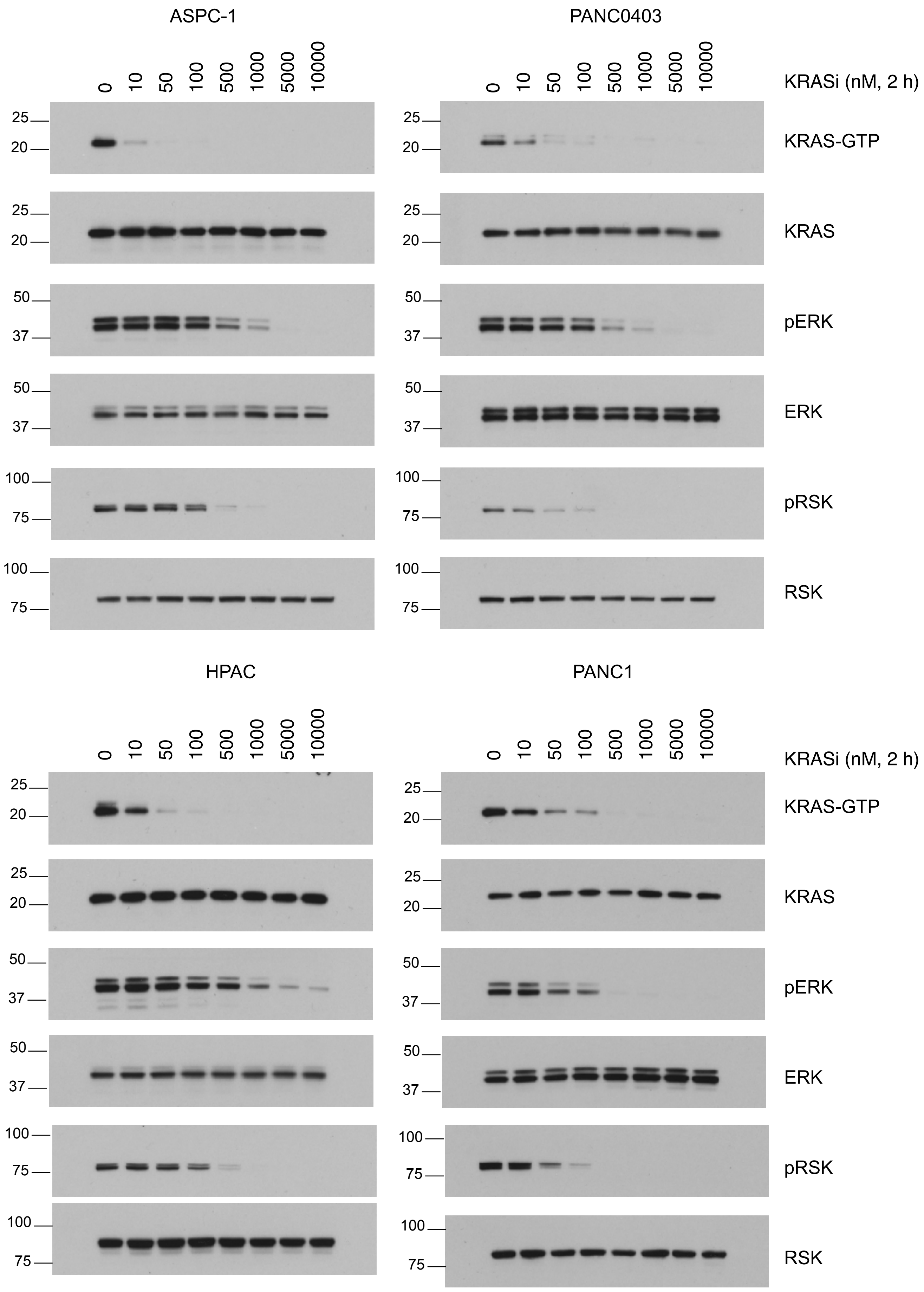

**Extended Data Figure 5**

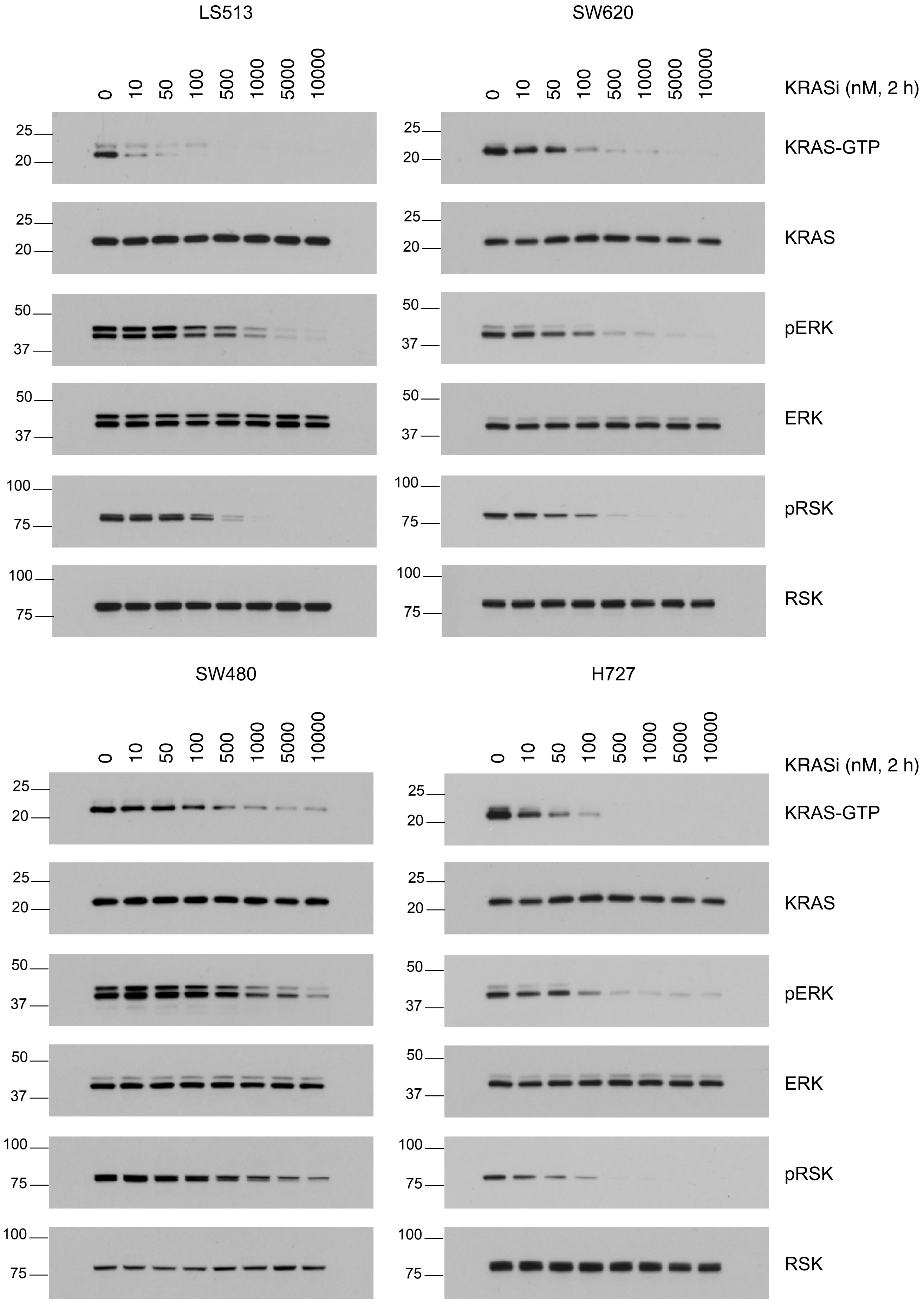

## Extended Data Figure 5

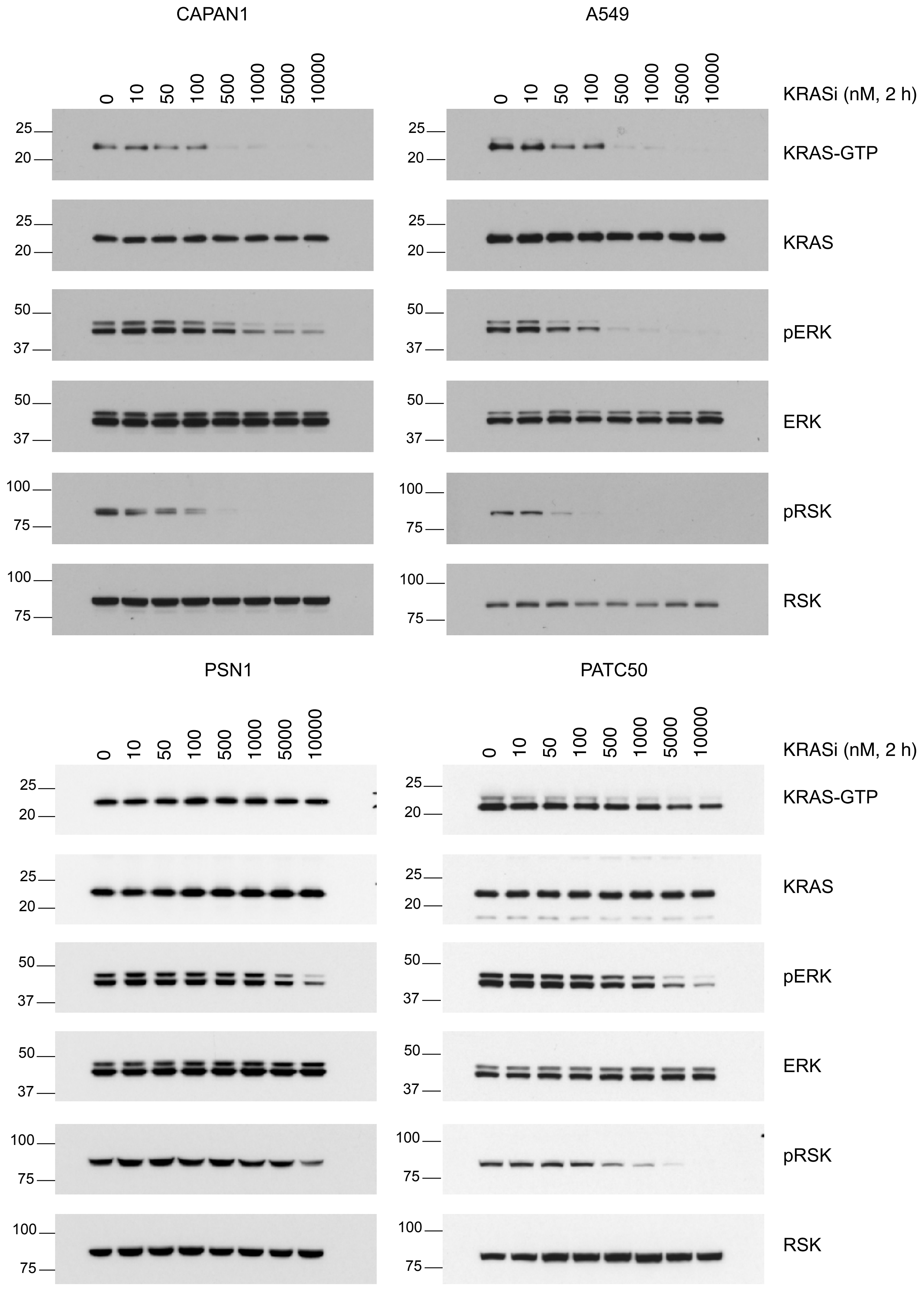

**Extended Data Figure 5**

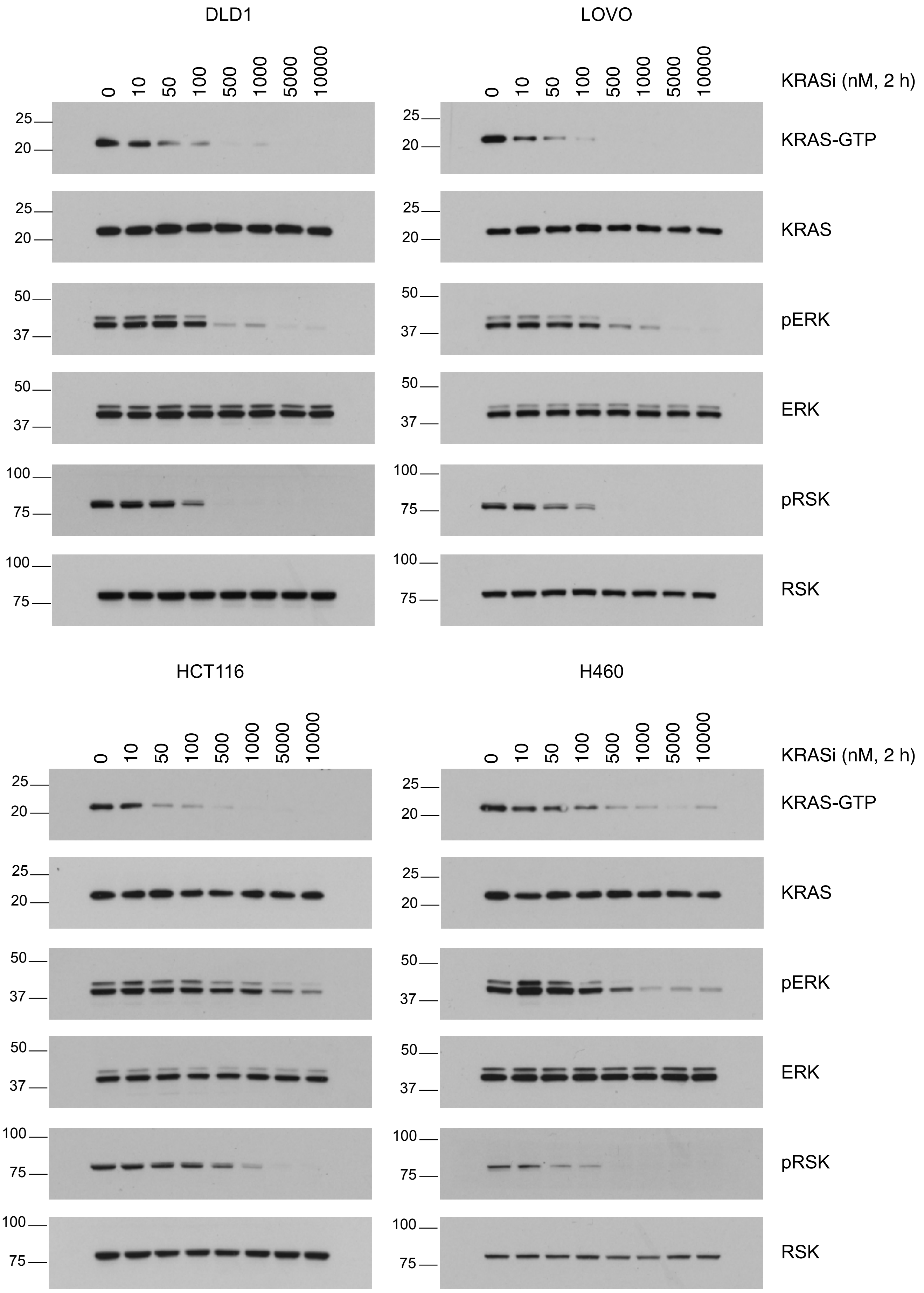

Extended Data Figure 5

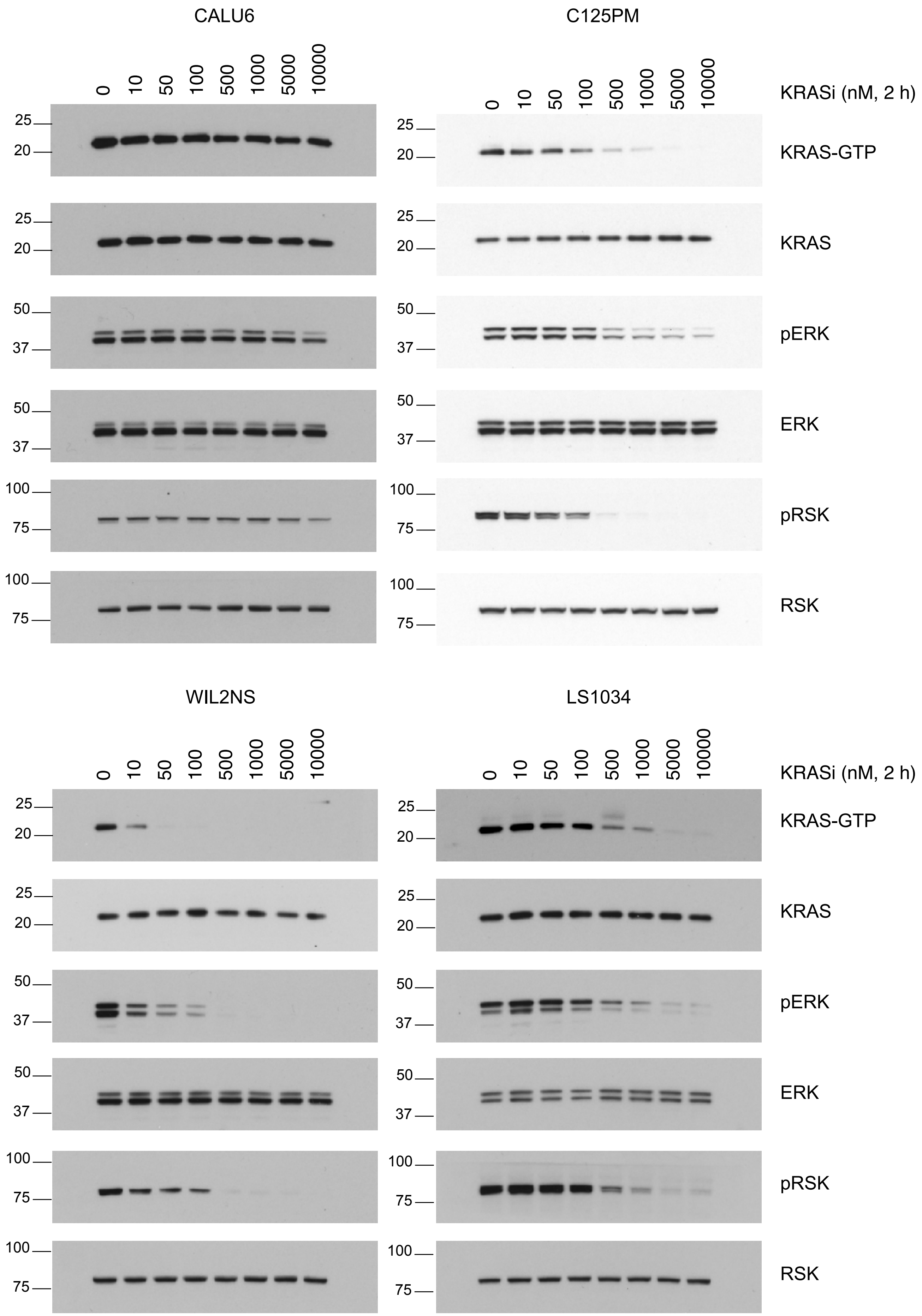

**Extended Data Figure 6a**

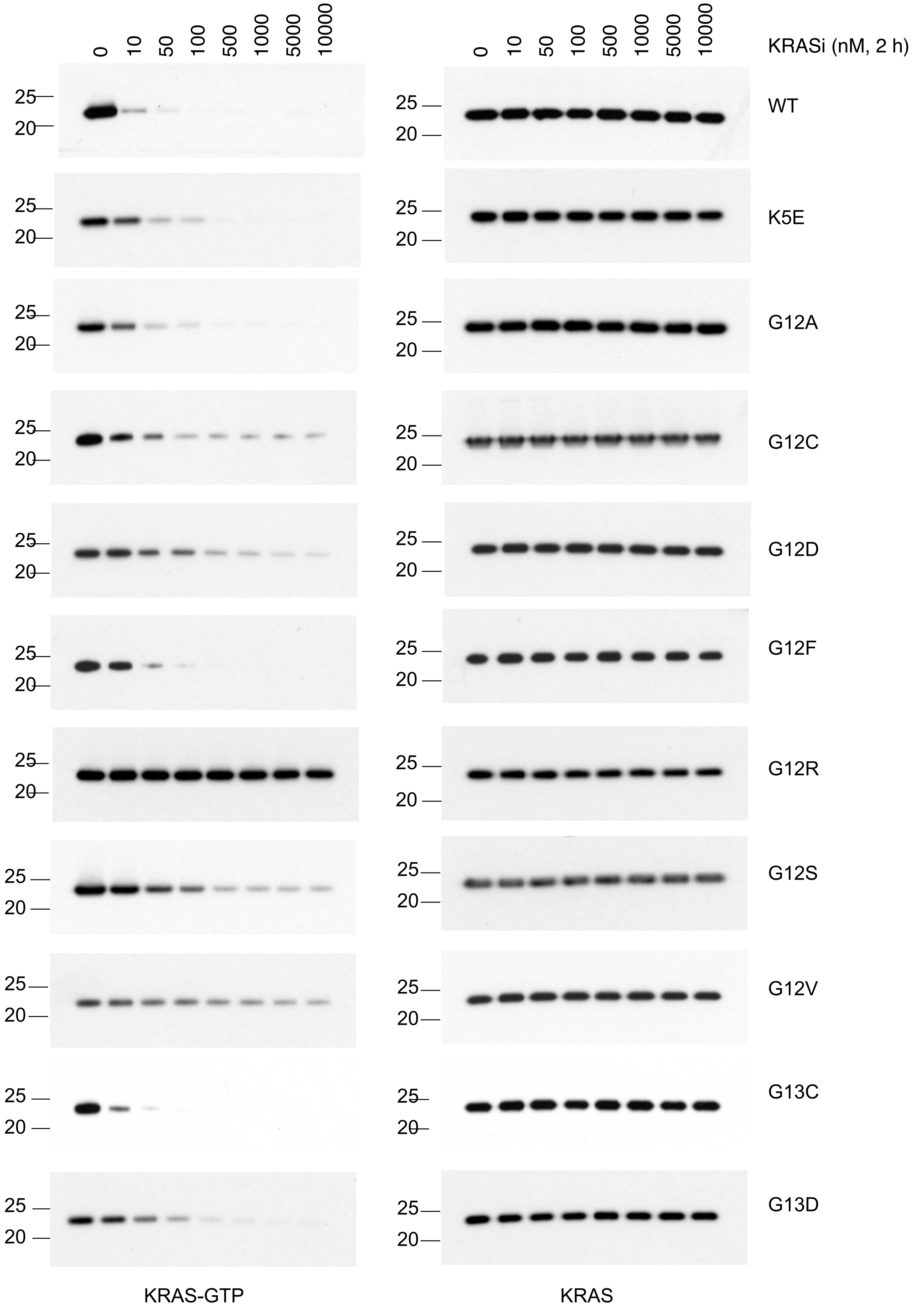

**Extended Data Figure 6a**

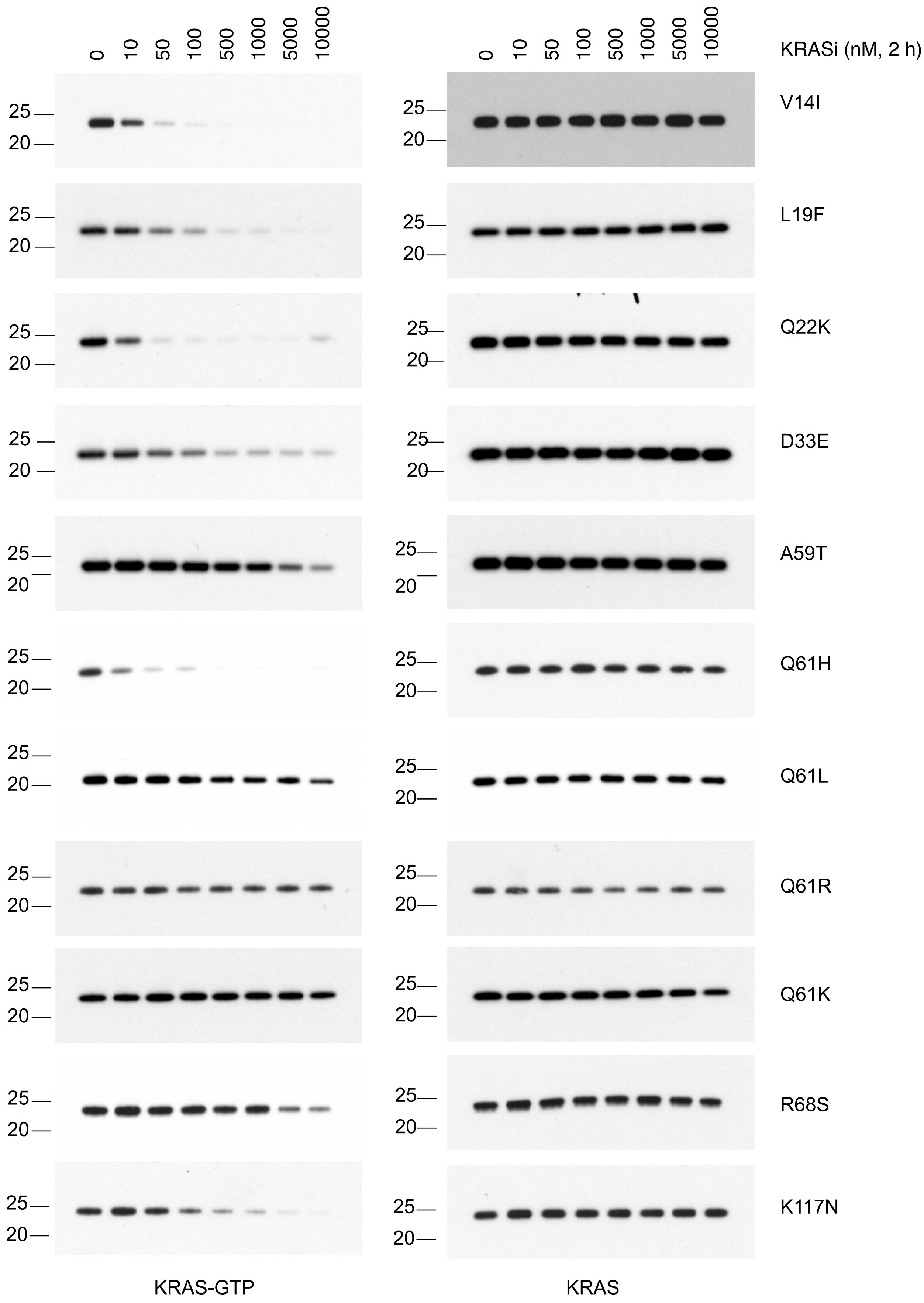

**Extended Data Figure 6a**

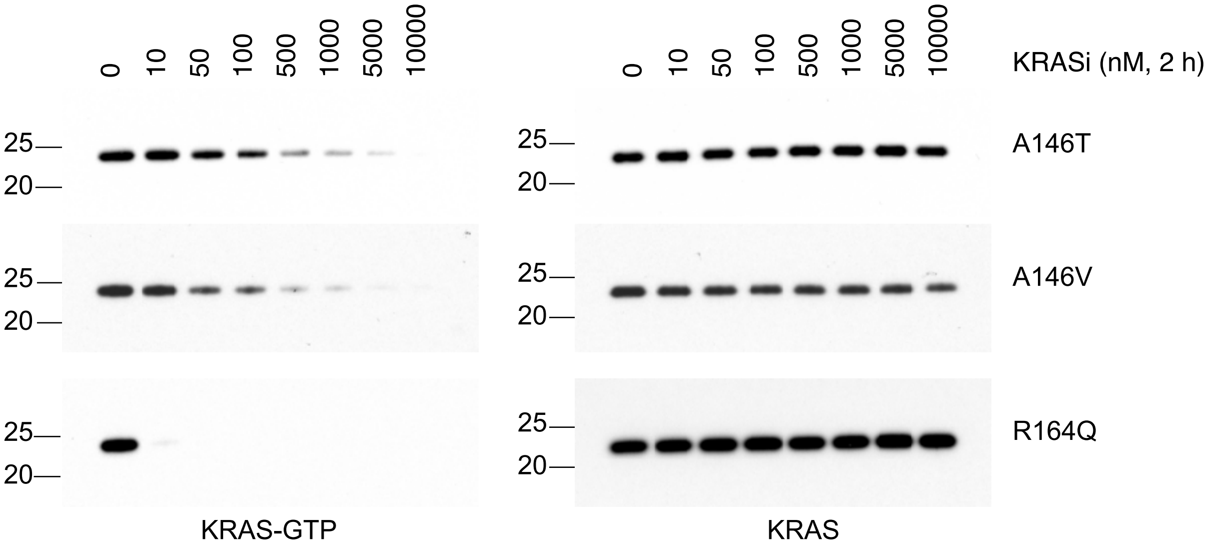


**Extended Data Figure 6b**

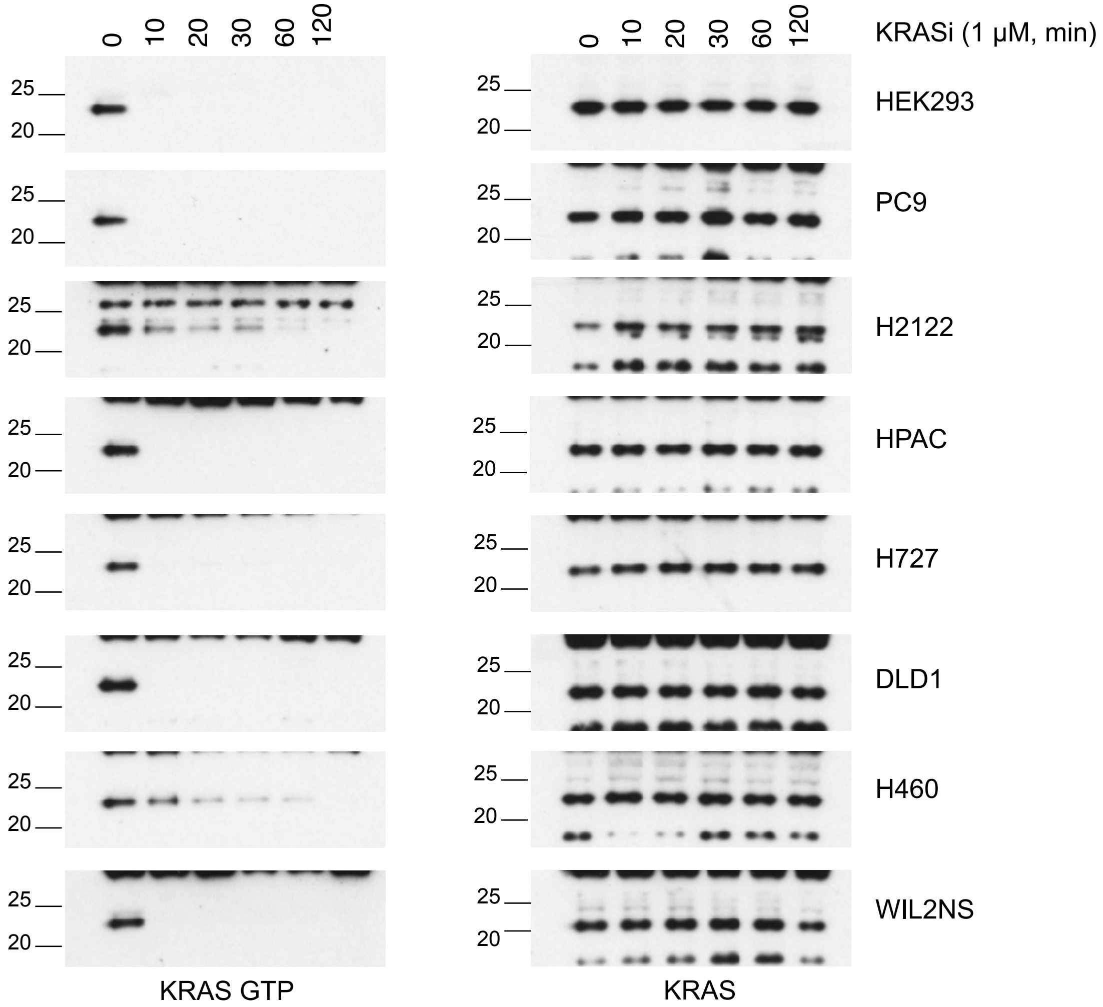

**Extended Data Figure 6c**

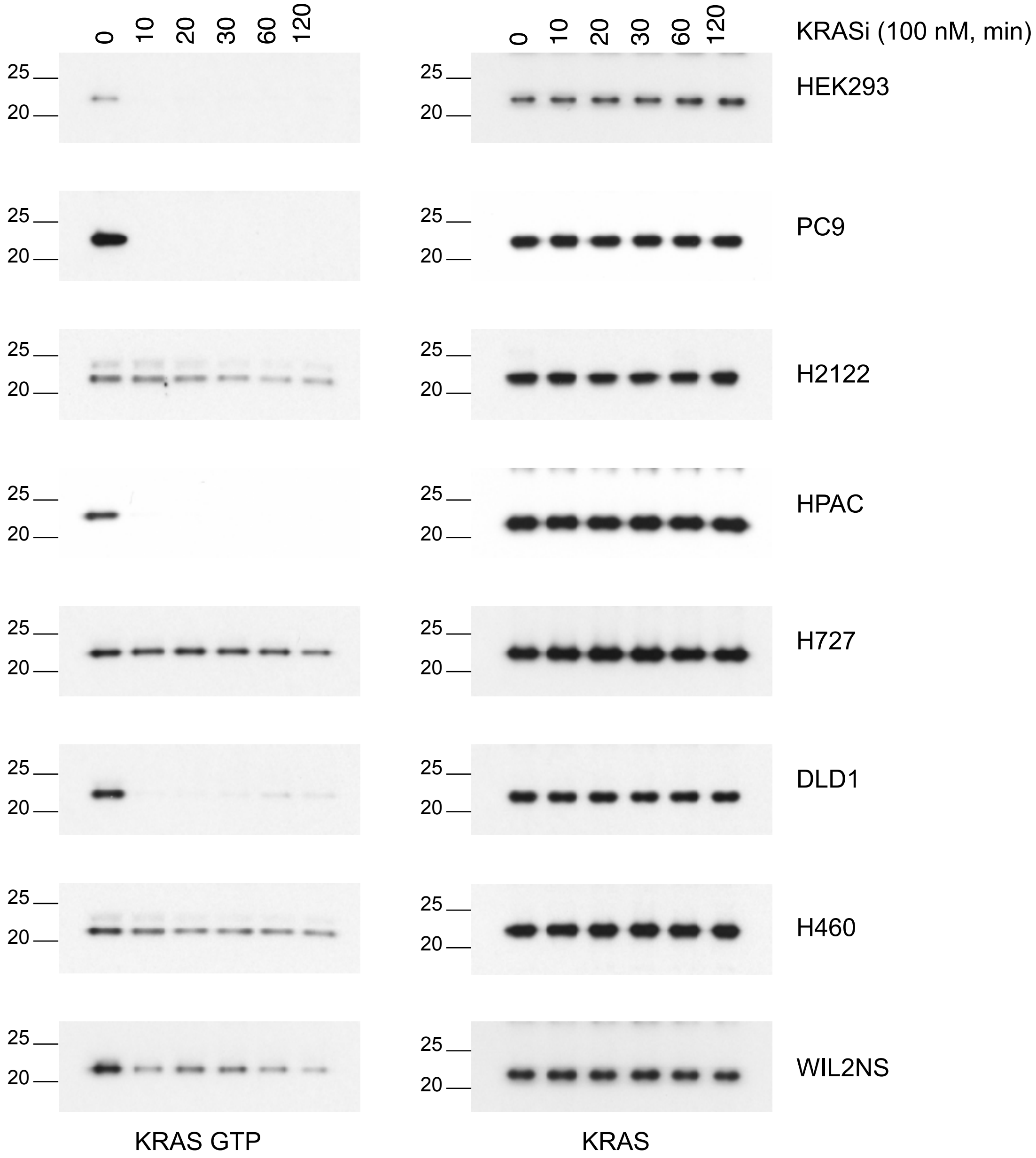

**Extended Data Figure 6d**

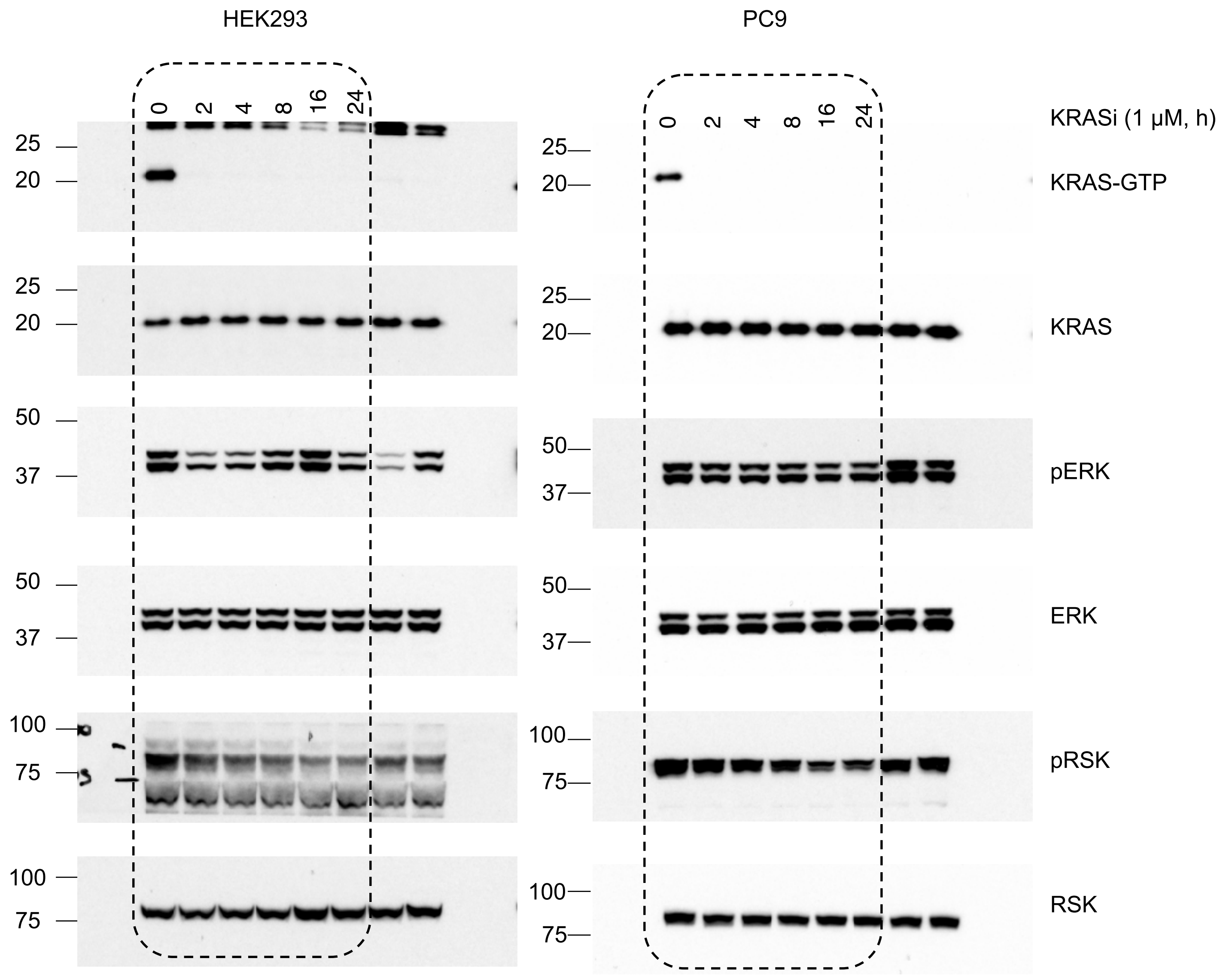

**Extended Data Figure 6d**

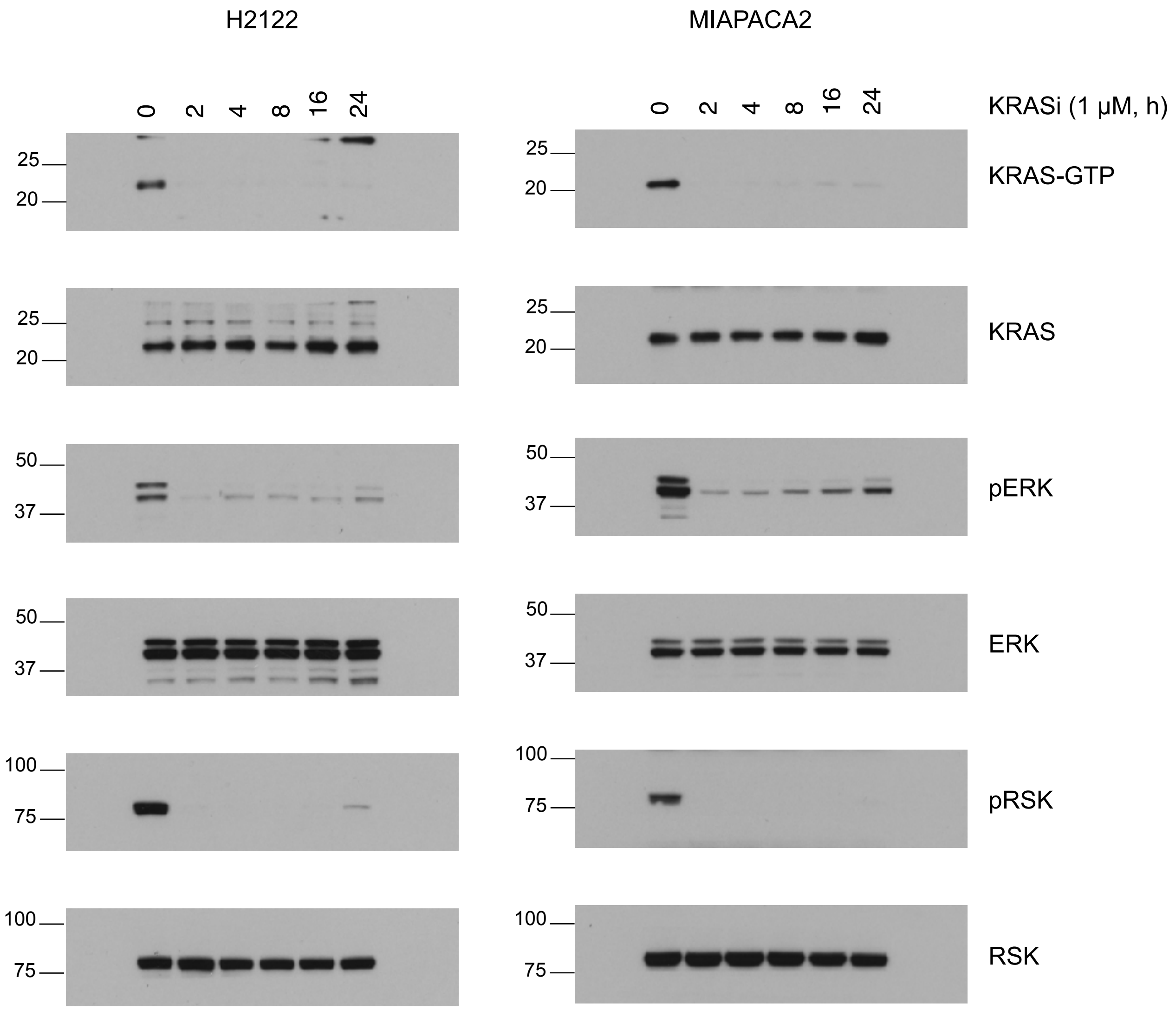

**Extended Data Figure 6d**

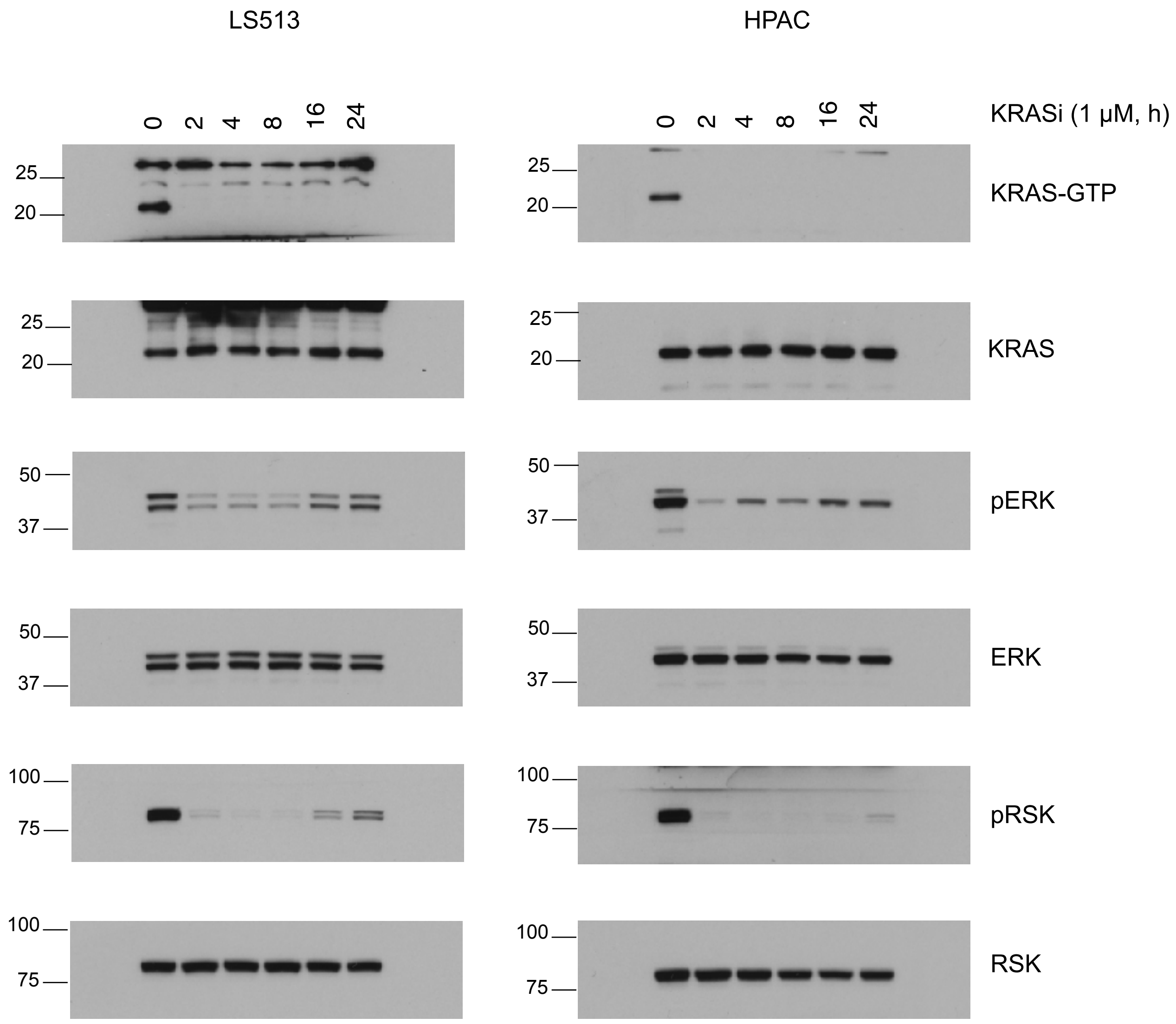

**Extended Data Figure 6d**

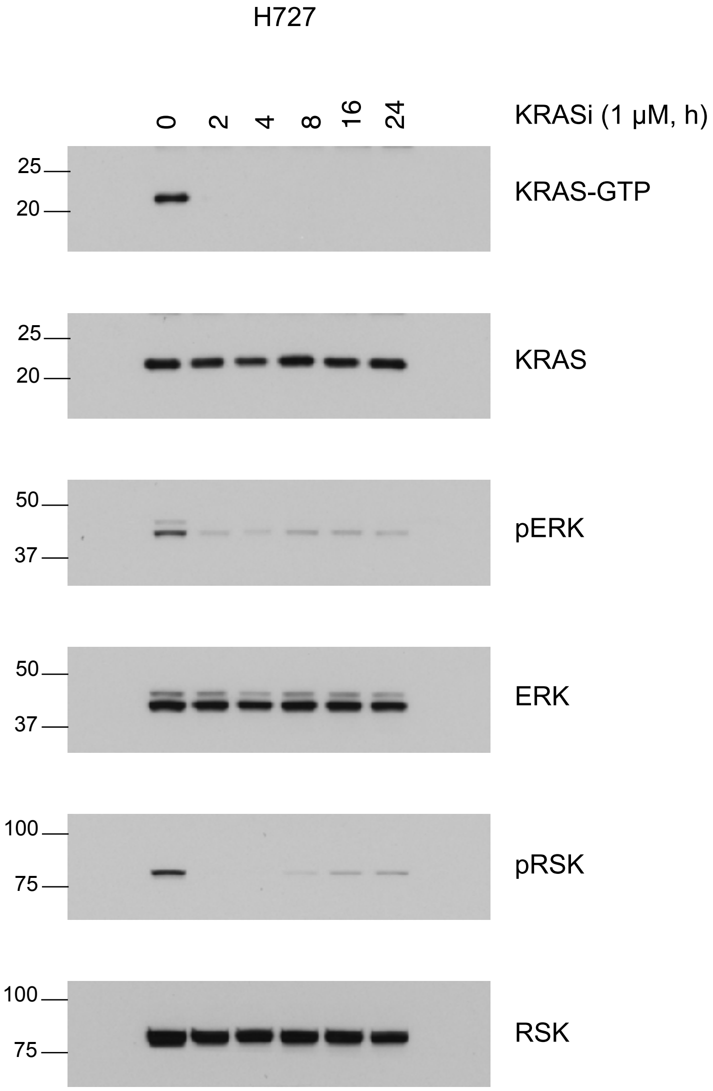

**Extended Data Figure 7a**

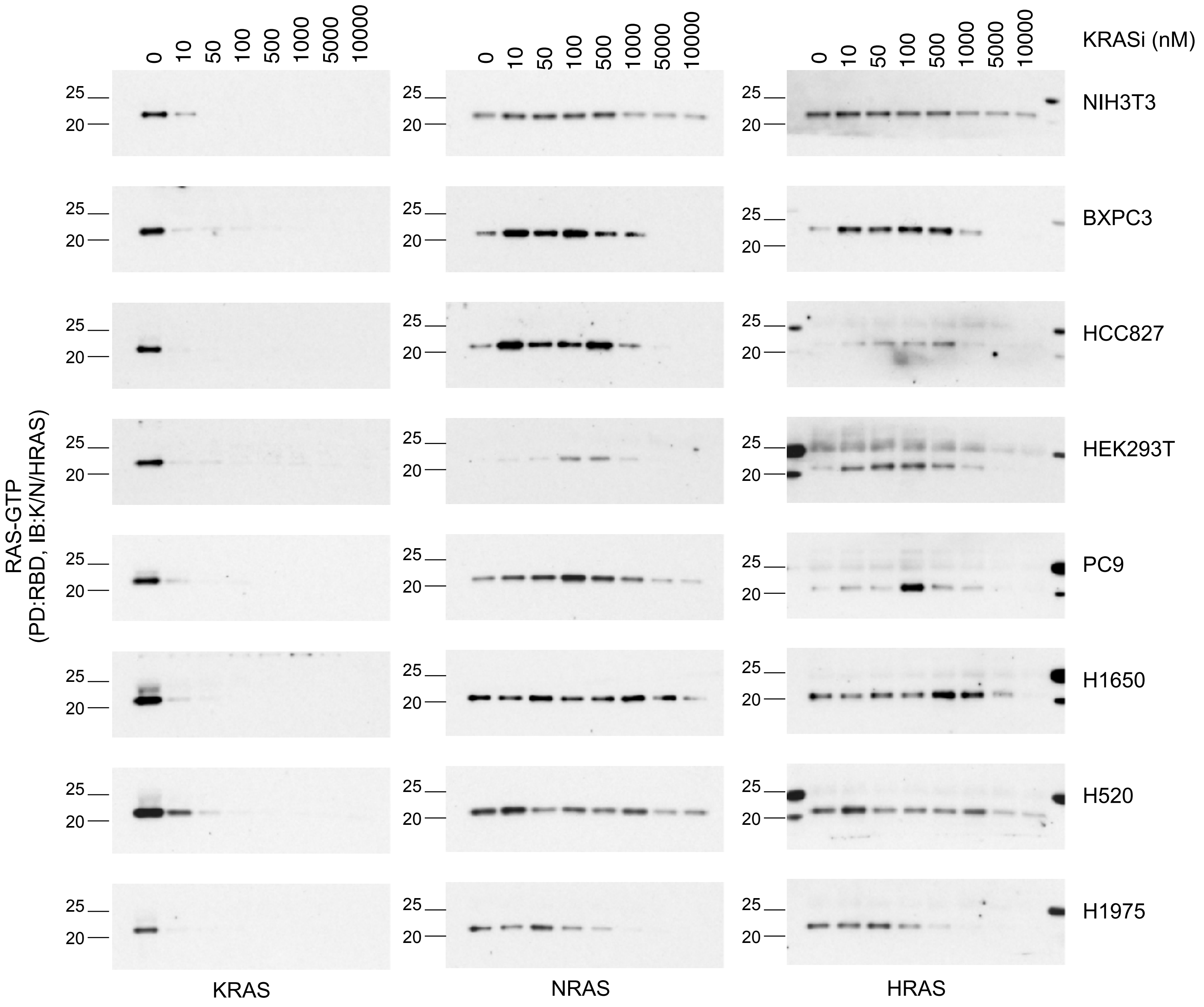

**Extended Data Figure 7a**

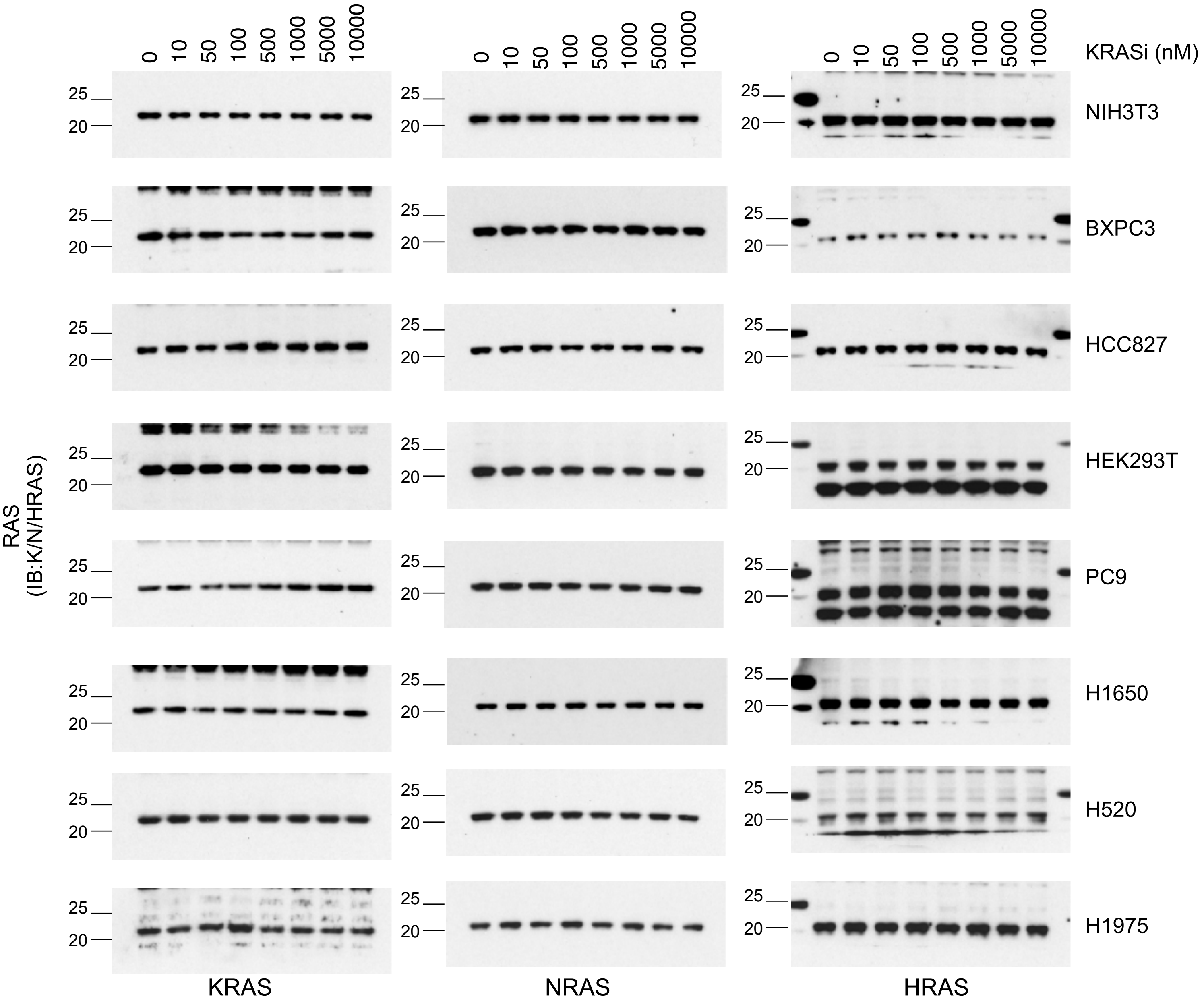

**Extended Data Figure 7c**

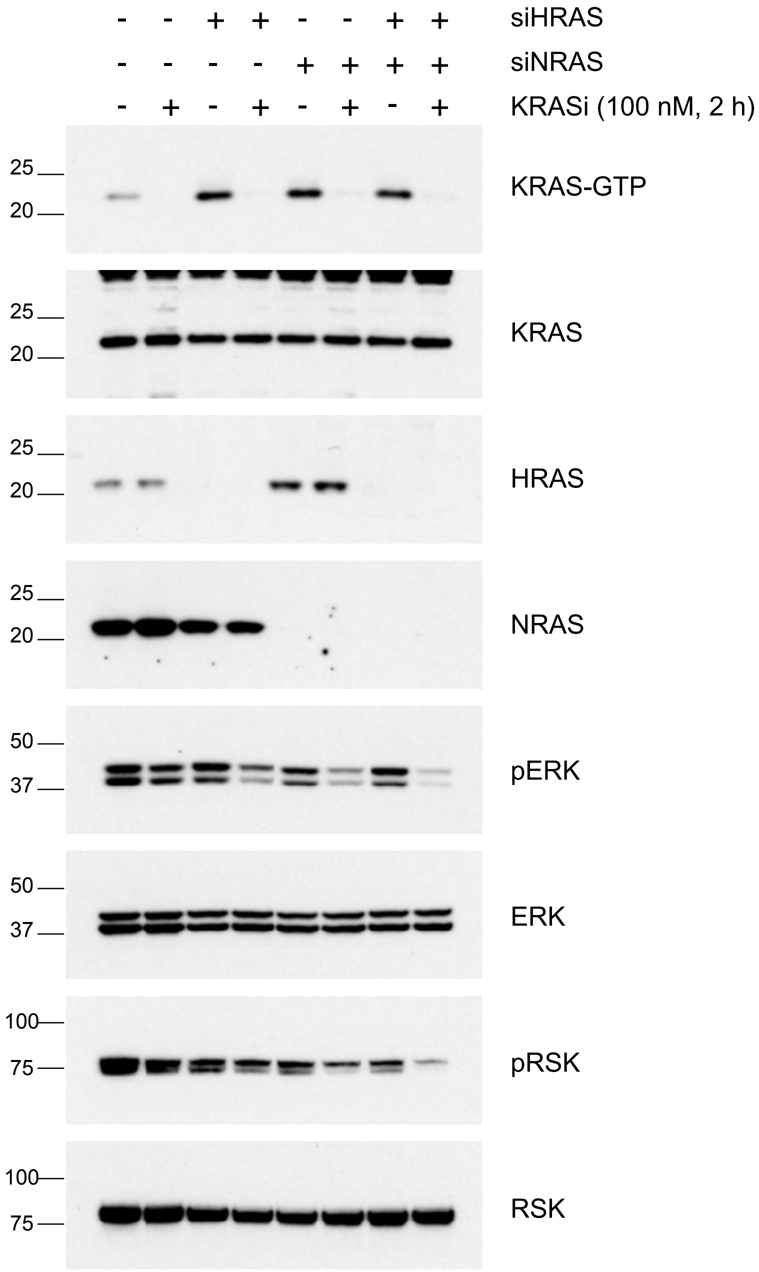

**Extended Data Figure 9g**

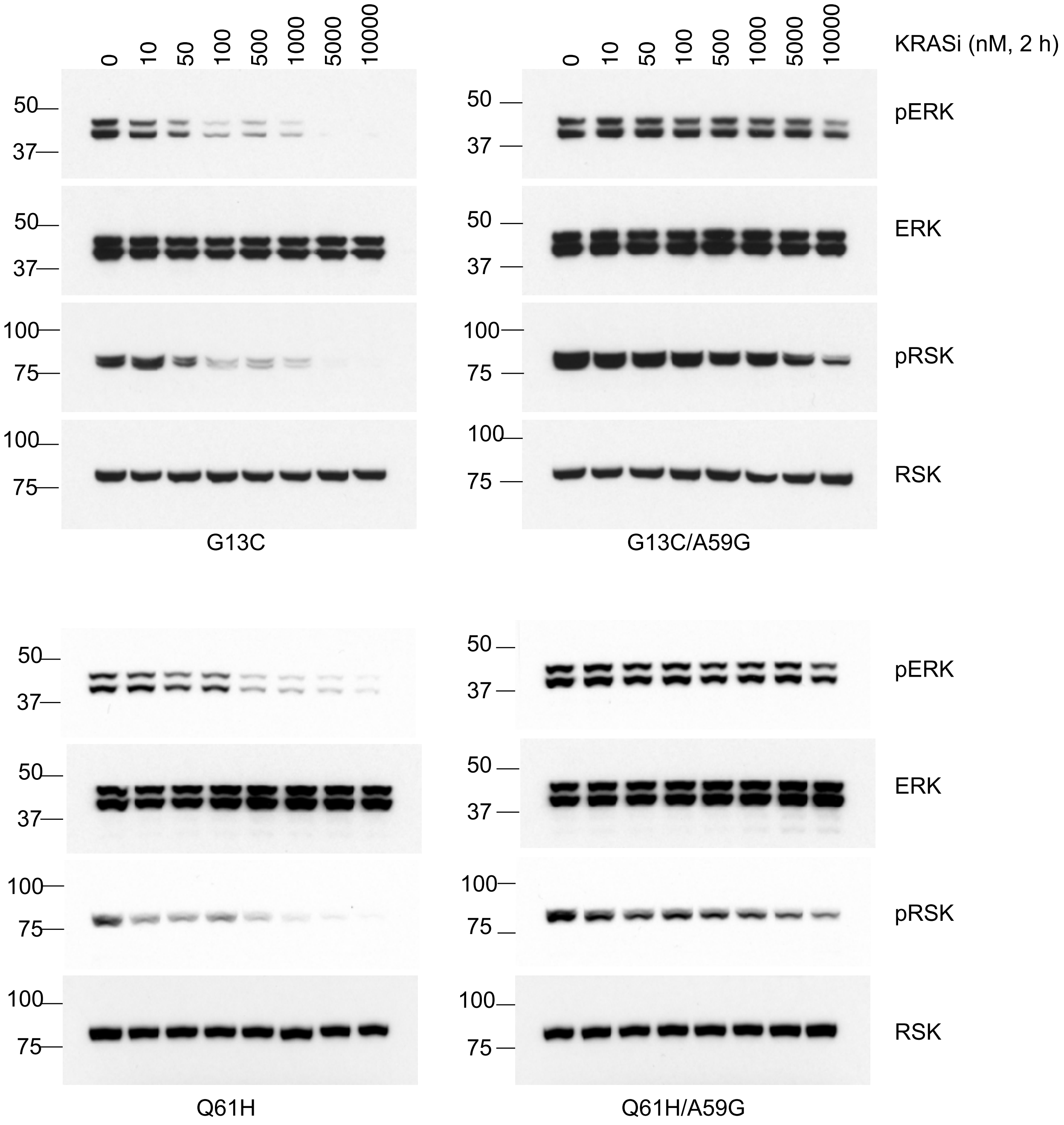

**Extended Data Figure 9h**

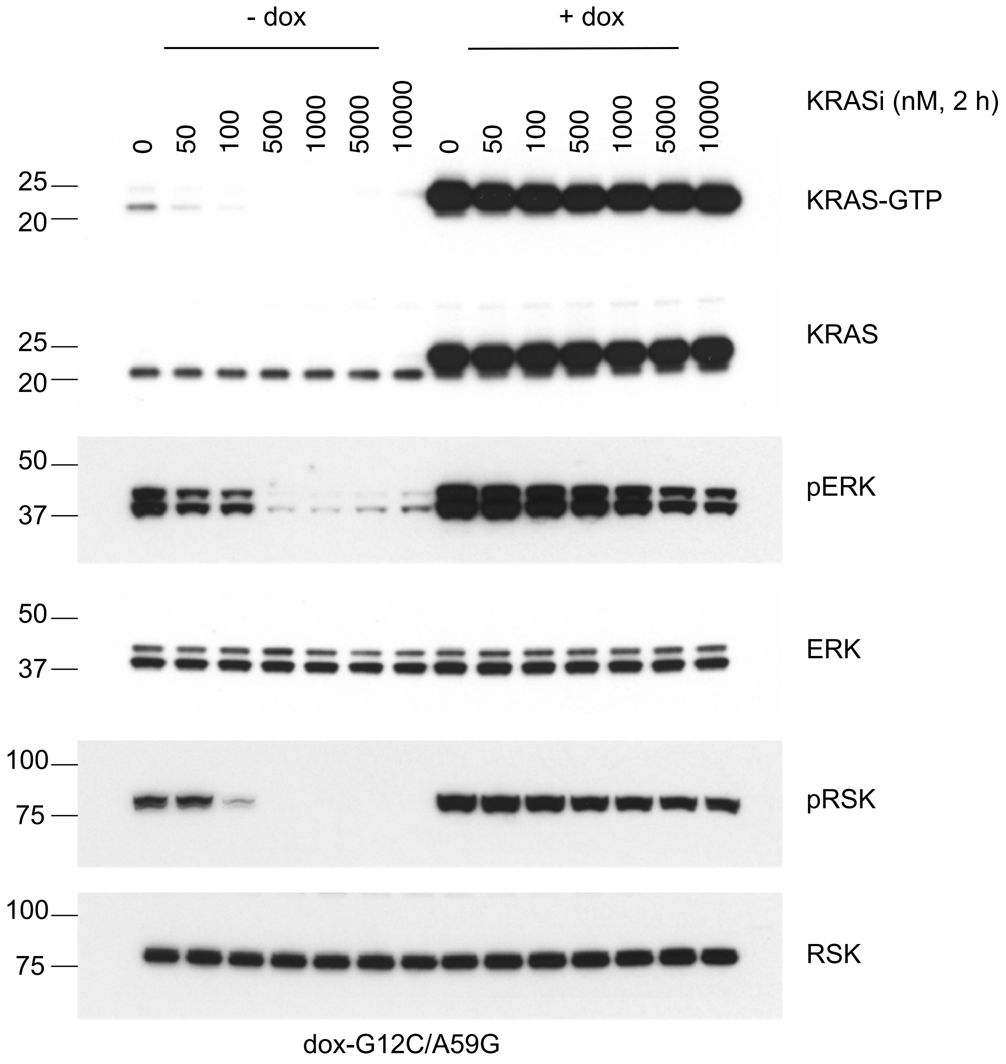


**Extended Data Figure 10c**

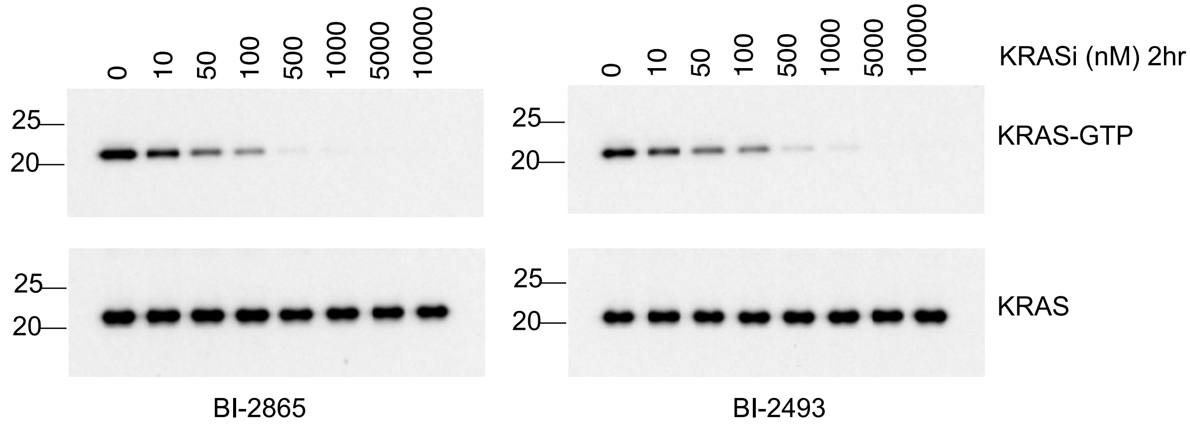

**Extended Data Figure 10d**

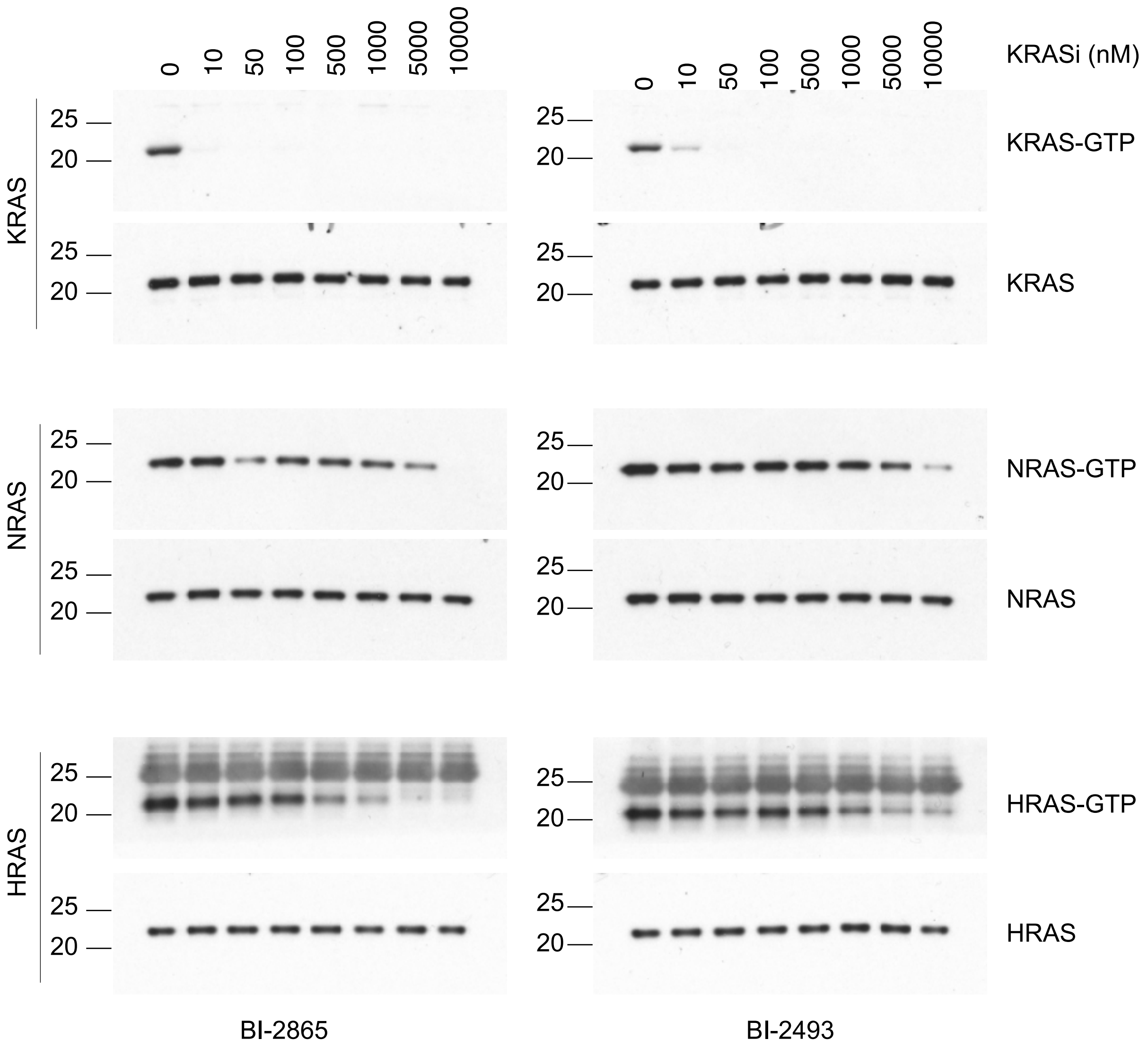

Supplement: Supplementary file 1 — This file contains Supplementary Discussion, Synthetic Methods, Figs. 1–4 and Refs. [file 41586_2023_6123_MOESM1_ESM.pdf]
