# Supplementary material for: Pan-KRAS inhibitor disables oncogenic signalling and tumour growth
Source: Nature. 2023 May 31;619(7968):160–6. doi: 10.1038/s41586-023-06123-3 (PMC10322706; doi:10.1038/s41586-023-06123-3)
Supplement: Supplementary file 2 — Reporting Summary [file 41586_2023_6123_MOESM2_ESM.pdf]

nature portfolio

Corresponding author(s): Piro Lito, MD, PhD

Last updated by author(s): Apr 4, 2023

Reporting Summary

Nature Portfolio wishes to improve the reproducibility of the work that we publish. This form provides structure for consistency and transparency in reporting. For further information on Nature Portfolio policies, see our Editorial Policies and the Editorial Policy Checklist.

Statistics

For all statistical analyses, confirm that the following items are present in the figure legend, table legend, main text, or Methods section.

n/a	Confirmed	
☐	☒	The exact sample size (n) for each experimental group/condition, given as a discrete number and unit of measurement
☐	☒	A statement on whether measurements were taken from distinct samples or whether the same sample was measured repeatedly
☐	☒	The statistical test(s) used AND whether they are one- or two-sided<br>*Only common tests should be described solely by name; describe more complex techniques in the Methods section.*
☒	☐	A description of all covariates tested
☐	☒	A description of any assumptions or corrections, such as tests of normality and adjustment for multiple comparisons
☐	☒	A full description of the statistical parameters including central tendency (e.g. means) or other basic estimates (e.g. regression coefficient) AND variation (e.g. standard deviation) or associated estimates of uncertainty (e.g. confidence intervals)
☐	☒	For null hypothesis testing, the test statistic (e.g. F, t, r) with confidence intervals, effect sizes, degrees of freedom and P value noted<br>*Give P values as exact values whenever suitable.*
☒	☐	For Bayesian analysis, information on the choice of priors and Markov chain Monte Carlo settings
☒	☐	For hierarchical and complex designs, identification of the appropriate level for tests and full reporting of outcomes
☒	☐	Estimates of effect sizes (e.g. Cohen's d, Pearson's r), indicating how they were calculated

Our web collection on statistics for biologists contains articles on many of the points above.

Software and code

Policy information about availability of computer code

Data collection	For Saturation mutagenesis, PCR products were subjected to Hiseq analysis at 2x150 bp.<br>Bulk sequencing was carried out on HiSeq in a 2X 150bp paired end run with an average of 30 million paired reads per sample.<br>The diffraction data was collected at X06SA beamline of the Swiss Light Source (Paul Scherrer Institute).
Data analysis	For saturation mutagenesis, count files were generated by using the ORFcall software and the raw read counts of each treatment groups were analyzed using edgeR to determine changes.<br>For bulk sequencing results were aligned to GRCH38 using HISAT2 and transcripts were counted using HTSeq in Python. The count data matrix was then processed by using limma and edgeR in R/Bioconductor.<br>The diffracted images were processed with autoPROC34 and all structures were solved by molecular replacement using a previously solved structure. Model building and refinement was performed with standard protocols using CCP4, COOT, autoBUSTER v.2.11.2 (http://www.globalphasing.com) and Phenix35,36.<br>For westernblot analysis, ImageJ was used.

For manuscripts utilizing custom algorithms or software that are central to the research but not yet described in published literature, software must be made available to editors and reviewers. We strongly encourage code deposition in a community repository (e.g. GitHub). See the Nature Portfolio guidelines for submitting code & software for further information.

Data

Policy information about availability of data

All manuscripts must include a data availability statement. This statement should provide the following information, where applicable:

- Accession codes, unique identifiers, or web links for publicly available datasets
- A description of any restrictions on data availability
- For clinical datasets or third party data, please ensure that the statement adheres to our policy

The data availability statement has been updated (in text and reporting forms). The data has been deposited in appropriate repositories.
The pan KRAS inhibitors ("GDP-KRAS inhibitors") are available as part of a collaborative program via Boehringer Ingelheim´s open innovation portal opnMe.com: https://opnme.com/collaborate-now/GDP-KRAS-inhibitor-bi-2493.
The co-crystal structures have been deposited in the PDB with accession codes: 8AZR, 8AZV, 8AZX, 8AZY, 8AZZ and 8B00.
The RNAseq results have been deposited in NCBI Geo with accession number: GSE228010
All other data are available upon request.

Human research participants

Policy information about studies involving human research participants and Sex and Gender in Research.

Reporting on sex and gender	No human subjects were used in this study.
Population characteristics	No human subjects were used in this study.
Recruitment	No human subjects were used in this study.
Ethics oversight	No human subjects were used in this study.

Note that full information on the approval of the study protocol must also be provided in the manuscript.

Field-specific reporting

Please select the one below that is the best fit for your research. If you are not sure, read the appropriate sections before making your selection.

☒ Life sciences ☐ Behavioural & social sciences ☐ Ecological, evolutionary & environmental sciences

For a reference copy of the document with all sections, see nature.com/documents/nr-reporting-summary-flat.pdf

Life sciences study design

All studies must disclose on these points even when the disclosure is negative.

Sample size	Varies across experiments. Please see methods section for more details.
Data exclusions	All data were used in statistical testing, unless otherwise specified in the text.
Replication	The experimental replicates are stated in the figure legends. All stated replicates indicate biological replicates.
Randomization	In vivo tumor growth experiments: mice were treated in a random manner with either vehicle or drug
Blinding	Tumor growth measurements were carried out in a blinded manner by a technician who was not aware of the goals of the experiment.

Reporting for specific materials, systems and methods

We require information from authors about some types of materials, experimental systems and methods used in many studies. Here, indicate whether each material, system or method listed is relevant to your study. If you are not sure if a list item applies to your research, read the appropriate section before selecting a response.

Materials & experimental systems

n/a	Involved in the study	
☐	☒	Antibodies
☐	☒	Eukaryotic cell lines
☒	☐	Palaeontology and archaeology
☐	☒	Animals and other organisms
☒	☐	Clinical data
☒	☐	Dual use research of concern

Methods

n/a	Involved in the study	
☒	☐	ChIP-seq
☒	☐	Flow cytometry
☒	☐	MRI-based neuroimaging

Antibodies

Antibodies used

Primary (1:1000 diultion, otherwise stated): Pan-Ras (1862335, Thermo), KRAS (WH0003845M1, sigma), HRAS(18295-1-AP, Proteintech. 1:300 diultion), NRAS (SC-31, santa cruz, 1:300 diultion)), FLAG M2(F1084, sigma,) HA (2367S, CST), GST (SC-138, santa cruz,) HIS tag (2365S, CST), ERK (4696S, CST), pERK (9101L, CST), pRSK(8753S, CST), RSK1/RSK2/RSK3(9355S, CST)
Secondary (1:3000 dilution): Mouse IgG HRP (7076S, CST), Rabbit IgG HRP (A4914, sigma)

Validation

We have validated the antibodies for pan-RAS,KRAS,HRAS and NRAS in this study by using siRNA targeting specific forms of RAS as well as using a 'RASless' cell lines that express only 1 form of RAS. All other antibodies have been validated in our previous publications. (Xue et al., Nature 2020, Zhao et al., Nature 2021 and Li et al., Science 2021)

Eukaryotic cell lines

Policy information about cell lines and Sex and Gender in Research

Cell line source(s)

ATCC: NIH/3T3 HEK283T, MRC5, MRC9, BXPC3, A375, H1299, H520, H1975, PC9, H1650, HCC827, U87MG, MEWO, H358, H2122, CALU1, MIAPACA2, ASPC1, PANC0403, HPAC, PANC1, LS513, SW620, SW480, H727, CAPAN1, A549, PSN1, PATC50, DLD1, LOVO, HCT116, H460, CALU6, WIL2NS, LS1034
Sigma: PC9, C125pm. Kerafast:U251MG, Accegen :BA/F3

Authentication

Cell lines were directly obtained from the vendor before use and studies were carried out before 20 passages upon purchase. Also cell lines were authenticated by MSK IMPACT sequencing to confirm the KRAS mutations. Cell lines bearing KRAS G12C mutation were also confirmed by testing with KRAS G12C specific inhibitors.

Mycoplasma contamination

The cell line used in the study were tested negative for mycoplasma.

Commonly misidentified lines
(See ICLAC register)

No commonly misidentified cell lines were used in the study.

Animals and other research organisms

Policy information about studies involving animals; ARRIVE guidelines recommended for reporting animal research, and Sex and Gender in Research

Laboratory animals

Athymic or NSG mice were used as indicated in the methods.

Wild animals

No wild animals were used in the study.

Reporting on sex

7-8 week old female nude mice were used for the study.

Field-collected samples

No field collected samples were used in the study.

Ethics oversight

Mice were housed according to the internal institutional and Austrian governmental and European Union guidelines (Austrian Animal Protection Laws, ETS-123) at BI or according to the Institutional Animal Care and Use Committee (IACUC) guidelines at MSKCC. All animal studies were approved by the internal ethics and the local governmental committee.

Note that full information on the approval of the study protocol must also be provided in the manuscript.
